# Public goods games on any population structure

**DOI:** 10.1126/sciadv.aeb1263

**Published:** 2026-03-06

**Authors:** Chaoqian Wang, Qi Su

**Affiliations:** ^1^ School of Mathematics and Statistics, Nanjing University of Science and Technology, Nanjing 210094, China.; ^2^ School of Automation and Intelligent Sensing, Shanghai Jiao Tong University, Shanghai, China.; ^3^ Key Laboratory of System Control and Information Processing, Ministry of Education of China, Shanghai, China.; ^4^ Shanghai Key Laboratory of Perception and Control in Industrial Network Systems, Shanghai, China.

## Abstract

Understanding the emergence of cooperation in social networks has advanced through pairwise interactions, but the corresponding theory for group-based public goods games (PGGs) remains less explored. Here, we provide theoretical conditions under which cooperation thrives in PGGs on arbitrary population structures, which are accurate under weak selection. We find that a class of networks that would otherwise fail to produce cooperation, such as star graphs, are particularly conducive to cooperation in PGGs. More generally, PGGs can support cooperation on almost all networks, which is robust across all kinds of model details. This fundamental advantage of PGGs derives from self-reciprocity realized by group separations and from clustering through second-order interactions. We also apply PGGs to empirical networks, which shows that PGGs could be a promising interaction mode for the emergence of cooperation in real-world systems.

## Teaser

A mathematical theory shows when cooperation emerges in public goods games on any network structure.

## INTRODUCTION

Cooperation is essential for the emergence of higher-level complexity in biological and social systems (*1*–*3*). To answer the question of how individual selfishness leads to selfless cooperation in evolution, many theories have been developed, such as evolutionary games on networks (also known as graphs). When individuals interact locally and adopt strategies with higher payoffs, cooperation may emerge through spatial reciprocity (*4*). Over the past three decades, agent-based simulations on networks have revealed numerous mechanisms that promote cooperation (*5*, *6*). In parallel, the analytical theory of pairwise games, initially on the regular graphs (*7*–*9*), has developed to identify the conditions for the success of cooperation on any population structure (*10*–*12*)—some networks are more conducive to cooperation than others (*13*, *14*).

However, pairwise games are limited in their ability to capture diverse natural and social phenomena, including nonlinear effects (*15*, *16*). Real-world interactions may involve more than two individuals. The evolution of cooperation in these group interactions is naturally described by multiplayer games (*17*). Applying the framework of evolutionary dynamics on graphs, a natural approach is to let individuals form groups with their neighbors and play multiplayer games within these groups. In this way, individuals participate not only in the groups they initiate but also in those initiated by their neighbors. Individuals then use the average or accumulated payoffs from these groups as the basis for strategy updates (*18*). This principle can be applied to any multiplayer game (*19*), including the public goods game (PGG) (*20*) and others (*21*, *22*).

The PGG is among the most widely studied multiplayer games, originating from the tragedy of the commons (*23*). Players choose whether to contribute to the common pool. All contributions are multiplied by a synergy factor and then evenly distributed among all players. From a group perspective, this amplification makes contributions collectively beneficial. However, from an individual perspective, one can still receive an equal share without contributing. This results in higher payoffs for noncontributors and incentivizes individuals not to contribute. Apart from human experiments (*24*–*27*), previous research on PGGs has primarily relies on agent-based simulations (*20*, *28*). Although agent-based simulations allow for great flexibility in studying previously unknown mechanisms, they require intensive computational resources and make it difficult to identify the underlying principles behind the phenomena. Therefore, recent work has attempted to analyze these mechanisms at a theoretical level (*29*–*34*). The feasibility of such efforts depends on the extent to which mathematical theory can support them.

The most advanced analytical theory for PGGs remains limited to regular graphs. There is no efficient algorithm at general selection strengths due to computational complexity (*35*), but a feasible alternative is to analyze the weak selection limit (*36*). Building on this framework, Li *et al.* (*37*, *38*) developed a theory for PGGs in infinite structured populations using pair approximation, although this method cannot capture clustering effects or higher-order interactions. Su *et al.* (*39*, *40*) further addressed this limitation and derived a corresponding theory for PGGs in finite structured populations. Both of these theories were confined to homogeneous networks, where all individuals have the same number of neighbors. General results for heterogeneous networks remains lacking.

Here, we develop the analytical theory for the evolution of cooperation in PGGs on any population structure. We identify the critical synergy factor for the success of cooperation on different networks, which is accurate under weak selection. Using this theory, we examine a large number of networks, finding that a class of structures, especially the star graph, can strongly promote cooperation in PGGs. We also explore PGGs on a series of random networks, including random, small-world, to scale-free, and verify the general effects of local structures, such as clustering, on cooperation. Last, we analyze all small networks and four empirical networks and find that cooperation consistently emerge across various model details in PGGs, whereas in pairwise games, it cannot. These results imply that PGGs may represent a more plausible and effective interaction mechanism in the real world than pairwise games.

## RESULTS

### PGGs on any population structure

In a PGG of G players, each player chooses either cooperation (C) or defection (D). A cooperator pays a cost c and contributes to the common pool, whereas a defector contributes nothing. If there are $g_{C}$ ($0\leq g_{C}\leq G$) cooperators, the total contribution to the pool is $g_{C}c$. These contributions are multiplied by a synergy factor r (r>1) to produce the public goods ${\mathrm{rg}}_{C}c$. This amount is evenly redistributed among all G players, so each receives ${\mathrm{rg}}_{C}c/G$. Therefore, the payoffs for cooperation and defection, π*_C_* and π*_D_*, are

$$\pi_{C}=\frac{{\mathrm{rg}}_{C}c}{G}-c$$ (1a)

$$\pi_{D}=\frac{{\mathrm{rg}}_{C}c}{G}$$ (1b)

At the group level, the synergy factor r>1 makes cooperation collectively beneficial. On the other hand, defectors also receive the public goods produced by cooperators, resulting in higher payoffs for defectors than for cooperators. The social dilemma thus emerges.

In this work, we study the PGG on general unweighted networks. We consider a population of size N, whose node set is denoted by N={1,2,…,N}. Each node represents an agent in the population. The adjacency between agents i and j is represented by $k_{\mathrm{ij}}$: If they are neighbors, $k_{\mathrm{ij}}=1$; otherwise, $k_{\mathrm{ij}}=0$. The number of neighbors of agent i is thus $k_{i}=\sum_{j\in N}k_{\mathrm{ij}}$. For convenience, we denote the neighbor set of agent i as $N_{i}$: If j is i’s neighbor ($k_{\mathrm{ij}}=1$), then $j\in N_{i}$.

**Fig. 1.**
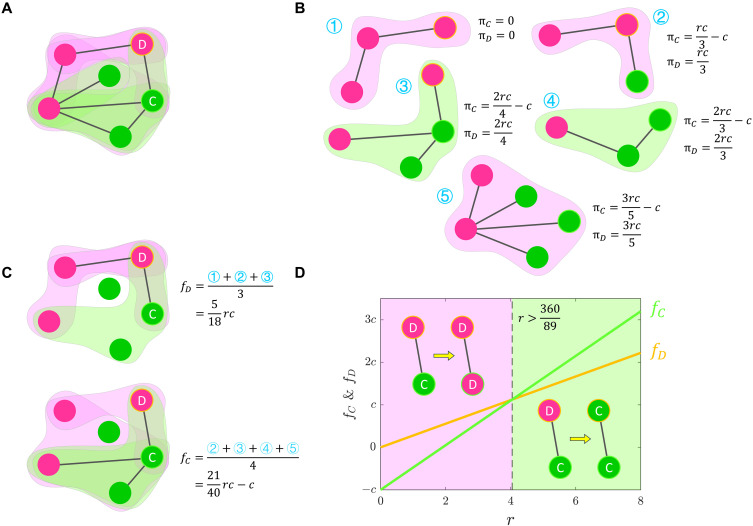
PGG on a general network. (**A**) Each agent organizes a PGG in the group of itself and its neighbors. (**B**) In each PGG, payoffs for cooperation and defection are calculated by Eq. 1. Obviously, $\pi_{D}>\pi_{C}$, defectors always have higher payoffs than cooperators, seemingly driving cooperation toward defection. (**C**) Because each agent organizes a PGG, an agent plays not only the PGG organized by itself but also the PGGs organized by its neighbors. We average the payoffs from these PGGs as an agent’s actual payoff. (**D**) Emergence of spatial reciprocity. For the presented two agents, their actual payoffs $f_{C}>f_{D}$ when r>360/89, driving defection toward cooperation.

On this network, each agent i organizes a group of size $G_{i}=k_{i}+1$, consisting of its neighbors and itself (Fig. 1A). A PGG is played within this group, and payoffs are calculated according to Eq. 1 (Fig. 1B). Also, agent i participates in the $k_{i}$ PGGs organized by its neighbors. Thus, agent i plays $1+k_{i}=G_{i}$ PGGs organized by itself and its neighbors. We take the average payoffs that agent i receives from these $G_{i}$ PGGs as the actual payoff, $f_{i}=\left(1/G_{i}\right)\sum_{j\in G_{i}}\pi_{i}^{\;j}$ (Fig. 1C), where $\pi_{i}^{\;j}$ denotes the payoff that agent i receives in the PGG organized by agent j and $G_{i}=\left\{i\right\}\cup N_{i}$ denotes the group organized by agent i. These actual payoffs $f_{i}$ determine strategy evolution (Fig. 1D).

In each elementary step, a random focal agent i is chosen to update its strategy. The actual payoff $f_{i}$ is calculated and mapped to fitness, $F_{i}=\text{exp}\left(\delta f_{i}\right)$ (*14*, *19*). Here, 0<δ≪1 is a weak selection strength, meaning that game payoffs contribute only a small perturbation to baseline fitness (*7*, *10*). The weak selection assumption reflects the idea that many other factors dominate the overall fitness of individuals. The actual payoffs and fitnesses of agent i’s neighbors are also calculated for comparison. Commonly used update rules, such as pairwise comparison (PC) (*41*), death-birth (DB) (*7*), and birth-death (BD) (*42*), vary in details but follow the same principle that strategies with higher payoffs have an advantage to propagate. We present the PC rule here as an example and study other update rules in Supplementary Note 1. The focal agent i selects a random neighbor $j\in N_{i}$ and adopts the strategy of agent j with probability

$$W_{i\leftarrow j}=\frac{1}{1+\text{exp}\left[-\delta \left(f_{j}-f_{i}\right)\right]}$$ (2)

Otherwise, agent i keeps the current strategy. The probability in Eq. 2 can also be interpreted as $W_{i\leftarrow j}=F_{j}/\left(F_{i}+F_{j}\right)$, with the probability of keeping the strategy understood as $F_{i}/\left(F_{i}+F_{j}\right)$.

The process then proceeds to the next elementary step, in which a different random focal agent i is chosen to update its strategy, and this process iterates (Methods). We track the fraction of cooperators $\rho_{C}$ in the population, which changes over time and may reach steady states after sufficiently many steps.

### Conditions for the evolution of cooperation

Although the cooperation fraction could fluctuate in nonequilibrium states for a very long time (*18*), it is expected to reach a fixation state eventually under weak selection, where all individuals choose to use the same strategies. There are only two fixation states: full cooperation ($\rho_{C}=1$) and full defection ($\rho_{C}=0$).

In a fully defective population, a mutant cooperator introduced at a particular node has a certain probability of spreading and eventually taking over the entire population. On heterogeneous networks, this probability generally depends on the node at which the mutant first appears. We define the fixation probability of a cooperator as the average takeover probability when the initial cooperator is placed uniformly at random. In other words, it is the likelihood that a single randomly positioned cooperator can convert a population of defectors into one of cooperators. Under neutral selection, i.e., when δ=0 so that payoffs from game interactions have no influence on the evolutionary process, the fixation probability of a cooperator is 1/N (*11*). Natural selection is said to favor the evolution of cooperation if the fixation probability of a cooperator exceeds this neutral benchmark.

We find that evolution favors cooperation in PGGs once the synergy factor r exceeds a critical threshold $r^{⋆}$. The critical synergy factor $r^{⋆}$ depends on the network and model details. Under the PC update rule, the condition for the success of cooperation on any network is

$$r>\frac{\tau^{\left(1\right)}}{{ϒ}^{\left(1\right)}}$$ (3)

Here, $\tau^{\left(n\right)}=\sum_{i,j\in N}k_{i}p_{\mathrm{ij}}^{\left(n\right)}\tau_{\mathrm{ij}}$ and ${ϒ}^{\left(n\right)}=\sum_{i,j\in N}k_{i}p_{\mathrm{ij}}^{\left(n\right)}{ϒ}_{\mathrm{ij}}$ can be obtained for a given network. First, $p_{\mathrm{ij}}^{\left(n\right)}$ is the probability of arriving at node j after an n-step random walks starting from node i. Second, $\tau_{\mathrm{ij}}$ [the coalescence times of ancestral random walks (*10*, *43*–*46*)] between nodes i and j are obtained by solving the following linear equations, with $\tau_{\mathrm{ji}}\equiv \tau_{\mathrm{ij}}$

$$\left\{\begin{array}{c} \tau_{\mathrm{ij}}=1+\frac{1}{2k_{i}}{\sum_{l\in N_{i}}}_{\mathrm{jl}}+\frac{1}{2k_{j}}\sum_{l\in N_{j}}\tau_{\mathrm{il}},\;\text{if}\;j\neq i\\ \tau_{\mathrm{ij}}=0,\;\text{if}\;j=i \end{array}\right.$$ (4)

Third, ${ϒ}_{\mathrm{ij}}$ can be calculated from the coalescence times $\tau_{\mathrm{ij}}$

$${ϒ}_{\mathrm{ij}}=\frac{1}{G_{i}}\left[\frac{\tau_{\mathrm{ij}}+\sum_{l\in N_{i}}\left(\tau_{\mathrm{jl}}-\tau_{\mathrm{il}}\right)}{G_{i}}+\sum_{l\in N_{i}}\frac{\left(\tau_{\mathrm{jl}}-\tau_{\mathrm{il}}\right)+\sum_{\ell \in N_{l}}\left(\tau_{j\ell }-\tau_{i\ell }\right)}{G_{l}}\right]$$ (5)

Note that ${ϒ}_{\mathrm{ii}}=0$ because $\tau_{\mathrm{ii}}=0$. However, ${ϒ}_{\mathrm{ij}}\neq {ϒ}_{\mathrm{ji}}$ in general because of differences in group sizes on heterogeneous networks.

We are thus able to determine the condition for cooperation in PGGs on all networks. Similar to Eq. 3, we also identify the condition under the DB rule (at each elementary step, a random individual i dies, and neighbors $j\in N_{i}$ compete for the vacant site proportionally to their fitness $F_{j}/\sum_{l\in N_{i}}F_{l}$), which is $r>\tau^{\left(2\right)}/{ϒ}^{\left(2\right)}$. See Supplementary Note 1 for the conditions under the BD rule and full theoretical derivations for other model details.

### Synthetic networks

We start the discussion from synthetic networks, which are uniquely determined by their network parameters. The conditions for the success of cooperation in PGGs can be expressed in terms of these network parameters.

**Fig. 2.**
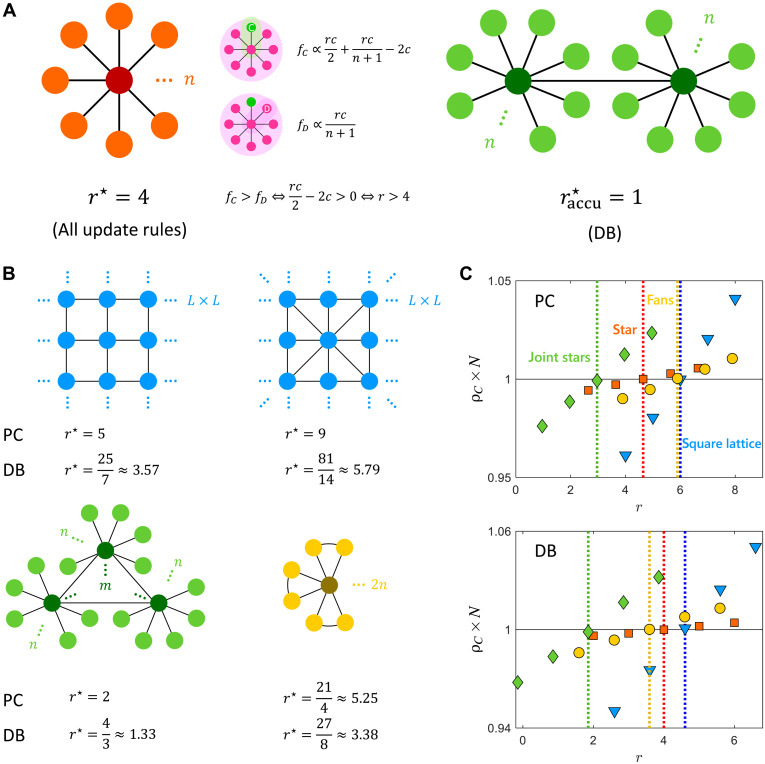
Cooperation conditions for PGGs on homogeneous and heterogeneous networks. (**A**) Star graphs support cooperation in PGGs as simple as when r>4, and the essence of which is 2×2: There are two groups for a leaf, with two players in its own group. When using accumulated payoffs, connecting the hubs of two or more star graphs leads to $r_{\text{accu}}^{⋆}=1$ under the DB rule, which maximally supports cooperation. (**B**) Comparison with other networks, including square lattices with von Neumann (left) and Moore (right) neighborhoods, joint stars with any number of hubs, and ceiling fans. The $r^{⋆}$ values reported here are for infinite population size (see Supplementary Note 2 for finite size). (**C**) Agent-based simulations confirm the theoretical predictions on L=5 square lattice with von Neumann neighborhood, n=9 star, m=3, n=9 joint stars, and n=9 ceiling fans. The dots represent the average cooperation fraction in the steady states (Methods), whereas the dashed lines are theoretical $r^{⋆}$ over which $\rho_{C}>1/N$.

A simple example is the star graph, composed of one hub and n leaves (Fig. 2A). We find that star graphs consistently promote cooperation in PGGs for r>4 under all update rules (and in the infinite population limit n→∞). This differs from the previous conclusion in pairwise donation games (DGs), where cooperation cannot emerge on star graphs (*10*). In this case, the so-called graph surgery, such as connecting the hubs of two stars, was a way to rescue cooperation in pairwise games. Here, if we further connect two hubs, we get a super structure to support cooperation in PGGs: With a variation of model details (accumulated instead of average payoffs), the condition for the success of cooperation is r>1; that is, cooperation is maximally favored.

The intuition of $r^{⋆}=4$ for star graphs can be interpreted as 2×2, two groups for a leaf times two players in the group organized by the leaf. We explain this under the DB update rule, for which $r^{⋆}\equiv 4$ holds independently of the population size. Because the focal agent’s payoff does not influence strategy updates (only neighbors compete for the focal vacant position), a focal leaf always takes the strategy of the hub neighbor. On the other hand, when the hub updates, the competition happens among all leaves and is independent of the hub. Therefore, the hub’s payoff does no work, and we only need to discuss the competition among all leaves. A leaf participates in two PGGs, organized by itself and the hub neighbor. In the PGG organized by itself, the payoff is $r\left(1+x_{H}\right)c/2-c$ if cooperating or ${\mathrm{rx}}_{H}c/2$ if defecting ($x_{H}=1$ if the hub cooperates and $x_{H}=0$ if it defects), where the overlapping term ${\mathrm{rx}}_{H}c/2$ can be eliminated. In the PGG organized by the hub, the payoff is ${\mathrm{rg}}_{C}c/\left(n+1\right)-c$ or ${\mathrm{rg}}_{C}c/(n+1)$, where the overlapping term ${\mathrm{rg}}_{C}c/\left(n+1\right)$ can be eliminated. In this way, a cooperator leaf has a higher payoff than a defector leaf if and only if (rc/2−2c)/2>0 or r/2−2>0. That is, $r>r^{⋆}=2\times 2=4$.

On the L×L square lattices with periodic boundary conditions of different neighborhood sizes (G=5 and G=9), our results agree with previous findings on regular networks (*31*, *40*) (Fig. 2B). Observing these results, we notice that star graphs can be even more conducive to cooperation than regular graphs as the critical synergy factor rises with group size on regular graphs but remains constant on star graphs. For example, the square lattice of G=9 has $r^{⋆}\approx 5.79$ under DB, which is worse than the constant $r^{⋆}=4$ of the star graph.

Figure 2B also shows results on more heterogeneous networks. For m fully connected hubs with n leaves on each, we have $r^{⋆}=4m/\left(2m-1\right)$ (PC update) and $r^{⋆}=\left(12m-4\right)/\left(9m-7\right)$ (DB update) for large n, which are $r^{⋆}=2$ and $r^{⋆}=4/3$ for large m. As another simple extension, for ceiling fans with n fans (each has two leaves), we have $r^{⋆}=21/4$ for PC update and $r^{⋆}=27/8$ for DB update. The DB update promotes cooperation on ceiling fans, whereas the PC rule inhibits it (compared to the original star graph). This is because the DB rule can use the clustering coefficient to promote cooperation in PGGs, which will be explained in the next section. Our predictions for these synthetic networks are also validated by agent-based simulations under different update rules (Fig. 2C).

### General roles of local structures

**Fig. 3.**
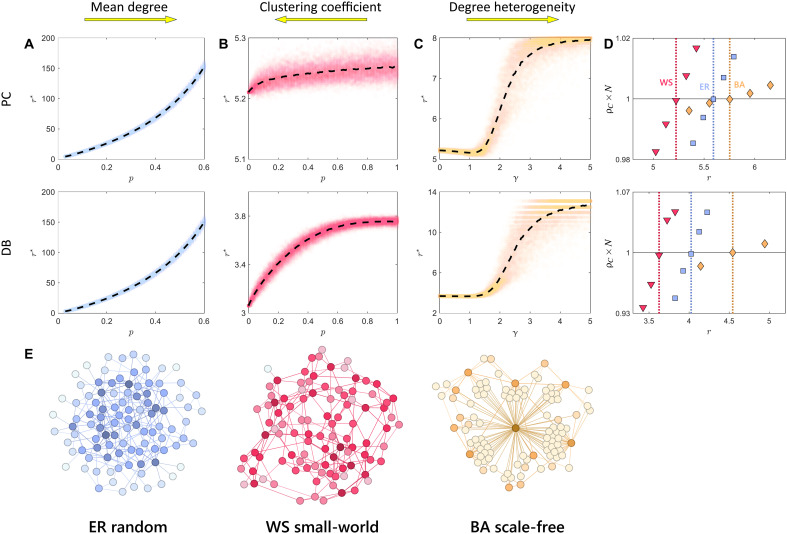
Effects of local structures on cooperation in PGGs. (**A**) Critical synergy factor $r^{⋆}$ as a function of connecting probability p on random graphs (ER) (*47*). The increasing average degree inhibits cooperation. (**B**) $r^{⋆}$ as a function of rewiring probability p on small-world networks (WS) (*48*) with d=2. The increasing clustering coefficient promotes cooperation. (**C**) $r^{⋆}$ as a function of generated degree distribution heterogeneity γ on scale-free networks (BA) (*51*, *52*) with m=2. The increasing degree heterogeneity initially promotes but ultimately inhibits cooperation. In (A) to (C), each point on dashed lines is the average result of 1000 randomly generated networks [totaling 58,000 in (A), 101,000 in (B), and 101,000 in (C)]. All networks are of size N=100 and are connected. (**D**) Agent-based simulations on selected sample networks confirm theoretical predictions. For ER, p=4/99; for WS, d=2, p=0.5; for BA, m=2, γ=2. (**E**) Visualization of the sample networks in agent-based simulations.

Next, we investigate the general roles of local structures on classic random networks, including random (ER), small-world (WS), and scale-free (BA) as shown in Fig. 3 (A to C). These networks are randomly generated by given parameters.

Random networks reflect the effect of average degree on cooperation when heterogeneity is present (Fig. 3A). We use the Erdős-Rényi (ER) algorithm (*47*) to generate random networks, which leads to a Poisson degree distribution. Among N=100 nodes, there can be at most N(N−1)/2 edges. Let the probability of an edge existing be p, then the average degree of the network is approximately 〈k〉≈p(N−1), which increases with p. We find that the critical synergy factor in PGGs increases with p, which indicates that an average degree increase is detrimental to cooperation. This is consistent with previous findings for pairwise games (*7*, *10*).

Small-world networks (WS) reflect the role of clustering coefficients (Fig. 3B). We use the Watts-Strogatz algorithm (*48*) to generate small-world networks, starting from a ring network of N=100 where each node has 2d neighbors within distance d=2 on both sides. Then, each node rewires the other end of each edge with probability p (the same edge cannot rewire twice; no self-loops or duplicate edges). The clustering coefficient, measuring the abundance of triangles, is approximately $\frac{3\left(d-1\right)}{2\left(2d-1\right)}{\left(1-p\right)}^{3}$ (accurate in large populations) (*49*), which decreases as p increases. The critical synergy factor $r^{⋆}$ increases with p, which indicates that high clustering promotes cooperation. This result is consistent with previous findings for regular graphs (*40*) (Supplementary Note 2), which can be understood as an impact of “higher-order interactions” (*50*). Notably, high clustering only notably promotes cooperation under the DB rule. On the one hand, agents interact with second-order neighbors in PGGs (whereas pairwise games only involve first-order neighbors). On the other hand, the essence of DB update is competition with second-order neighbors, whereas the PC rule is with first-order neighbors (*9*). The combination of second-order game interactions and strategy competitions provides enough reach to be influenced by triangles.

Scale-free networks reflect the impact of degree heterogeneity on cooperation (Fig. 3C). We use the algorithm proposed by Krapivsky *et al.* (*51*), which is an extension of the Barabási-Albert (BA) (*52*), to generate scale-free networks. Compared to the original BA model, this algorithm allows for adjusting degree heterogeneity directly. We start with m=2 isolated initial nodes. The remaining N−m=98 nodes then join the existing network one by one. Each new node attaches to m existing nodes [hence the approximate average degree 〈k〉=2m(1−m/N)]. The probability of selecting node i is proportional to $k_{i}^{\gamma }$, where γ is the strength of preferential attachment, determining the degree heterogeneity. When γ=1, we reduce to the standard BA scale-free network. We find that increasing degree heterogeneity γ initially slightly promotes cooperation but ultimately hinders cooperation. In other words, a moderate network heterogeneity is most conducive to cooperation (*53*). Such a nonmonotonic transition in cooperation is due to a complex interplay of multiple factors. For example, increasing degree heterogeneity may also change the clustering coefficient. This is different from some previous studies on pairwise games (*54*, *55*).

The conclusions are more subtle across other model details (fig. S2). The theoretical results on these random networks are also supported by agent-based simulations (Fig. 3D) on selected structures (Fig. 3E). According to these simulations with consistent average degrees, the small-world network (〈k〉=4) is most conducive to cooperation, the random (〈k〉≈4) is secondary, and the scale-free (〈k〉=3.84) is least conducive.

### Fundamental advantages of PGGs

**Fig. 4.**
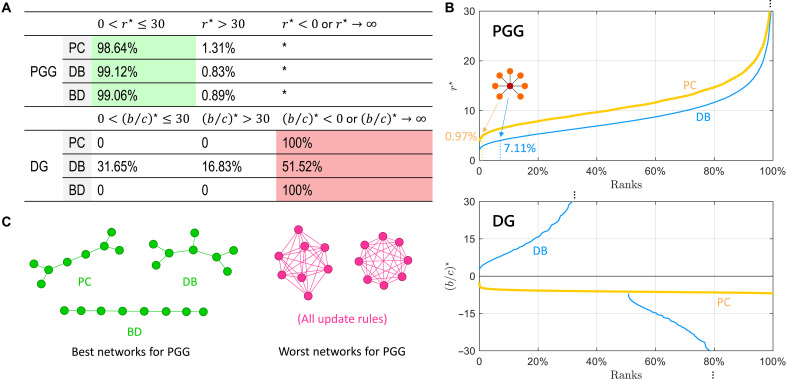
Cooperation emerges consistently in PGGs on almost all networks under different update rules, outperforming pairwise DGs. (**A**) Among all 12,111 networks of sizes 3≤N≤8, the fraction of networks classified by their critical synergy factors. Almost all networks have $0<r^{⋆}\leq 30$ in supporting cooperation. The symbol * means that the only structure that does not support cooperation is the fully connected network. In contrast, for pairwise DGs, cooperation is only possible under DB update, with more than half of the networks not supporting cooperation. (**B**) Ranks of all 11,117 networks of size N=8 in supporting cooperation for PGGs and DGs under PC and DB update rules. The star graph ranks the top 0.97% (PC) and 7.11% (DB). The results in PGGs are consistent under different update rules, whereas in pairwise DGs, they are quite different. (**C**) Best and worst networks of size N=8 for cooperation in PGGs. The best networks differ with update rules. The worst two networks are consistent under all update rules, which are the fully connected (right) and a similar network (left).

We further investigate the conditions for cooperation in PGGs across all small networks. For population sizes 3≤N≤8, there are 2 (N=3), 6 (N=4), 21 (N=5), 112 (N=6), 853 (N=7), and 11,117 (N=8) possible networks, respectively. The critical synergy factors on these 12,111 networks are summarized in Fig. 4A. There are 98.64% (PC), 99.12% (DB), and 99.06% (BD) networks where the critical synergy factors are $0<r^{⋆}\leq 30$. The conditions for the success of cooperation are relaxed on almost all networks under various update rules. There are only a few networks (1.31% for PC, 0.83% for DB, and 0.89% for BD) with strict cooperation conditions $r^{⋆}>30$. The last category is $r^{⋆}<0$ or $r^{⋆}\to \infty$ (we also numerically categorize $r>10^{3}$ as r→∞ here). When $r^{⋆}<0$, the cooperation condition becomes $r<r^{⋆}$ and cooperation is impossible for meaningful r>1. The symbol * means that the only network that does not support cooperation in PGGs is the fully connected network.

In comparison, cooperation cannot thrive well in the DG (*10*), where a cooperator donates b to the other player by paying c (b>c). The studied quantity here is the benefit-to-cost ratio, b/c, which has a critical value $b/c>{\left(b/c\right)}^{⋆}$ over which cooperation is favored. We find the critical values ${\left(b/c\right)}^{⋆}$ fall within unfavorable intervals. Under PC and BD updates, no network can support cooperation [${\left(b/c\right)}^{⋆}<0$ or ${\left(b/c\right)}^{⋆}\to \infty$ for 100%]. Under the DB update, more than half of the networks (51.52%) cannot support cooperation, with only 31.65% of structures having relaxed cooperation conditions. These conclusions on group PGGs and pairwise DGs remain valid if we use accumulated payoffs (figs. S4 and S5).

From these insights, we see a fundamental advantage of PGGs: Cooperation can emerge on all networks (except fully connected) and under various update rules, which outperforms pairwise DGs. In PGGs, self-reciprocity plays a key role in the emergence of cooperation: A cooperator can generate personal benefits rc/G. Once the synergy factor exceeds the group size, r>G, a single cooperator can generate a net benefit independently (rc/G−c>0). To clarify, such self-reciprocity cannot induce cooperation alone. One example is the fully connect graph (rightmost of Fig. 4C), where the critical synergy factor is r→∞, and individuals do not choose cooperation even when r>N. This is because we did not assume individual intelligence that perceives the net benefit of cooperation. Instead, individuals follow the update rule (PC, DB, or others), in which individuals compare their payoff to their neighbors. Obviously, on the fully connected graph, the payoff of defectors is always higher than that of cooperators. Only a certain group separation on the network can make the net benefits from cooperation comparable, thus making individuals choose to cooperate. Therefore, we attribute the fundamental advantage of PGGs to self-reciprocity through group separation on networks, where the role of networks is indispensable.

We show the ranks of all 11,117 networks of size N=8 under PC and DB rules in Fig. 4B. Networks with smaller critical synergy factors $r^{⋆}$ are more advantageous for cooperation and appear earlier in the ranking. PC and DB updates show consistency in their rank trends, with the DB rule slightly more favorable for cooperation. In contrast, the ranks in pairwise DGs are quite different under PC and DB rules. This is curious because there is no qualitative difference between these update rules, which consistently assume the advantages of high payoffs in strategy evolution. Here, PGGs show another fundamental advantage: They perform consistently across various details in update rules.

In addition, it is worth mentioning that the star graph ranks in the top 0.97 and 7.11% for the PC and DB updates, which agrees with our previous conclusion that star graphs are among the most conducive networks for cooperation in PGGs. More generally, we present the best and worst networks of size N=8 as shown in Fig. 4C. The best networks vary in different update rules, but they all have low average degrees. From BD, PC, to DB rules, the best networks shift from linear to starlike in shape. The worst networks are consistent across all update rules, which have high degrees, from fully connected to similar structures.

### Robustness of PGGs on empirical networks

**Fig. 5.**
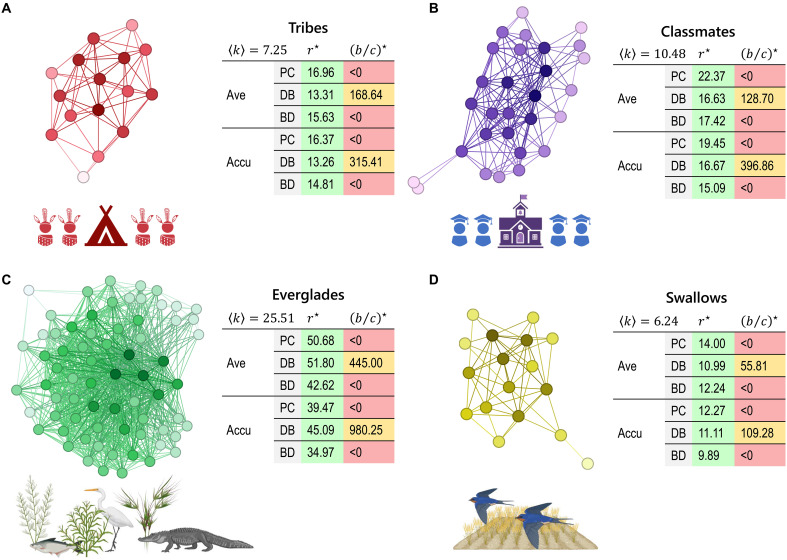
PGGs could be a promising interaction mode for cooperation on empirical networks. We analyze PGGs on four empirical networks, where the cooperation conditions are consistently relaxed across various model details in PGGs. In contrast, the conditions for cooperation are strict and inconsistent across different model details in pairwise DGs. The examined empirical systems include the following: (**A**) 16 tribes of the Gahuku-Gama alliance structure of the Eastern Central Highlands of New Guinea (*56*, *57*); (**B**) 29 seventh-grade students in Victoria, Australia, connected by whom they would prefer to work with (*58*); (**C**) the networks of trophic interactions that occur among the 69 major taxonomic groups of everglades habitats in South Florida ecosystems (*56*, *59*, *60*); and (**D**) the network of body contact interactions among 17 North American barn swallows (*56*, *61*).

The fundamental advantages of PGGs were presented on small networks of sizes N≤8, but we are also interested in the robustness of these advantages in large real-world systems. Here, we analyze four empirical networks and calculate the critical synergy factors for the success of cooperation on each of them (Fig. 5). The results are presented across various model details, including three update rules (PC, DB, and BD) and two payoff calculations [average (ave) and accumulated (accu)].

The first two cases are human societies, including a premodern and a modern social structure. The premodern example is 16 tribes of the Gahuku-Gama alliance structure of the Eastern Central Highlands of New Guinea (*56*, *57*) (Fig. 5A), with normalized critical synergy factors $r^{⋆}/\langle k\rangle =1.83∼2.33$ in PGGs and benefit-to-cost ratios ${\left(b/c\right)}^{⋆}/\langle k\rangle <0$ or ${\left(b/c\right)}^{⋆}/\langle k\rangle \geq 23.26$ in DGs. The modern social structure is 29 seventh-grade students in Australia based on their preferred working partners (*58*) (Fig. 5B), with normalized critical synergy factors $r^{⋆}/\langle k\rangle =1.44∼2.13$ and benefit-to-cost ratios ${\left(b/c\right)}^{⋆}/\langle k\rangle <0$ or ${\left(b/c\right)}^{⋆}/\langle k\rangle \geq 12.28$. The third case is an ecological structure, the trophic interactions among 69 major taxonomic groups of the various everglades habitats in South Florida ecosystems (*56*, *59*, *60*) (Fig. 5C), which has $r^{⋆}/\langle k\rangle =1.37∼2.03$ and ${\left(b/c\right)}^{⋆}/\langle k\rangle <0$ or ${\left(b/c\right)}^{⋆}/\langle k\rangle \geq 17.44$. The last case is an animal social network, constructed with edges of body contact interactions among 17 North American barn swallows (*56*, *61*) (Fig. 5D), with $r^{⋆}/\langle k\rangle =1.58∼2.24$ and ${\left(b/c\right)}^{⋆}/\langle k\rangle <0$ or ${\left(b/c\right)}^{⋆}/\langle k\rangle \geq 8.94$. Because the cooperation conditions increase with average degrees, we mentioned the corresponding normalized conditions above for comparison. According to these real data, we verify that PGGs can support cooperation on these real-world population structures with relaxed conditions and are robust across all model details. From this perspective, PGGs could be a promising interaction mode for cooperation in real-world systems.

On the other hand, we can see that the required critical synergy factors are always greater than the average degree (e.g., for primitive tribes, $r^{⋆}>7.25$), which means that these real networks are not as conducive to cooperation as regular graphs. Therefore, even with an interaction mode like PGGs that are conducive to cooperation, networks alone cannot fully explain the emergence of cooperation. There could be other factors that come together to support cooperation in these social structures.

We also see that accumulated payoffs are slightly more conducive to cooperation in PGGs (the only exceptions are the DB update in “classmates” and “swallows”). This is also consistent with our intuition that payoffs from different groups are physically accumulated in the real world. In contrast, the average payoffs serve normalization and theoretical analysis, which are less conducive to cooperation on the studied real-world networks.

## DISCUSSION

As a group-based interaction, PGGs capture the empirical fact that individuals not only interact with their direct friends but also with their second-order neighbors in the circle of the direct friends (*62*–*64*). Here, we develop an analytical theory to predict the evolution of cooperation in PGGs on arbitrary population structures. Given a network as input, the theory yields the cooperation condition for PGGs on that network, which is accurate under weak selection and can remain qualitatively consistent when selection becomes numerically stronger (fig. S3). Whereas prior works focused on pairwise games (*10*, *42*), our theory advances this line of research to group-based PGGs.

Using our theory, we find that a class of networks, such as the star graph, which were considered unfavorable for cooperation in pairwise games (*10*, *13*), can notably promote cooperation in PGGs. An intuitive explanation may be self-reciprocity embedded in PGGs, i.e., cooperation can yield a net benefit rc/G. Accordingly, when the net benefit exceeds the cost, rc/G>c or r>G, cooperation becomes individually favorable and the situation may not even constitute a social dilemma (*65*). However, we emphasize that self-reciprocity alone is insufficient to fully explain the emergence of cooperation in PGGs. One counterexample is fully connected networks, in which cooperation cannot emerge even when r>N. This is because update rules (e.g., PC) in evolutionary dynamics rely on local fitness-based imitation rather than rational decision-making. In other words, only the network creates certain group separations can individuals “perceive” the net benefit of cooperation. Our analytical framework captures such structural effects, showing that a nondilemma condition (*65*) must be complemented by network-driven group structure for cooperation to emerge in evolution.

These insights also raise questions about the role of evolution in cooperation. Evolution can not only promote cooperation beyond the reach of individual rationality but also hinder cooperation that rational individuals might otherwise pursue. A similar phenomenon was observed in the social goods dilemma (*14*), where evolution promotes prosocial behavior even when such behavior is not collectively optimal. Compared to the social goods dilemma—where interactions are limited to first-order neighbors and second-order edges—our PGG model incorporates second-order neighbors, capturing a broader scope of interaction. An even broader interaction structure was found in the diffusible public goods model (*36*), where public goods can spread to and influence more distant nodes. However, our work provides analytical results for the most widely studied setting (*18*, *20*), where the diffusion is minimal and thus more conducive to cooperation (*36*).

Given the typically high clustering coefficients in real-world networks (*48*, *66*), we highlight the importance of clustering effects arising from second-order interactions (*67*) in PGGs—an effect absent in pairwise games. Previous studies have also revealed clustering effects in PGGs, but these were confined to regular graphs (*31*, *40*). We demonstrate the positive role of clustering in promoting cooperation on general networks. Similar phenomena have also been observed in studies of higher-order networks (*50*, *68*, *69*). However, the notion of clustering in higher-order frameworks differs from that in traditional network models because they require a separately defined higher-order interaction graph. In addition, higher-order studies (*50*) primarily investigate the effects of nonlinear payoff functions (*15*, *16*), and their scope is limited to interactions involving no more than three individuals. By contrast, our framework supports standard PGGs with arbitrary group sizes on conventional networks.

More generally, we test all possible networks with sizes 3≤N≤8 and find that PGGs consistently promote cooperation (except on fully connected networks). In contrast, pairwise games fail to produce cooperation on most networks (*10*) (Fig. 4 and figs. S4 and S5). This difference can be attributed to how benefits and costs are accounted, or the self-reciprocity embedded in PGGs. As previously noted, this built-in feature relies on the network to manifest its effect. Our main contribution is to establish a general theory of PGGs on networks, which is equally important as the foundational theory of pairwise DGs.

We examine four empirical networks, including primitive tribes, junior students, everglades, and barn swallows. The PGG can produce consistent cooperation across all kinds of evolutionary details on these real networks. The results thus imply that PGGs could be a promising interaction mode for the emergence of cooperation in real-world systems. However, these real-world networks do not maximize cooperation. Their cooperation thresholds are stricter than those of regular graphs with the same average degree. In the real world, cooperation is shaped by the coexistence of multiple behavioral and social mechanisms (*70*), such as memory of past interactions, conformity to social norms, and concerns about punishment or reward. By excluding these mechanisms, our analysis isolates the effect of network structure, and under such minimal assumptions, it is expected that the critical synergy factors $r^{⋆}$ appear relatively large.

Although real-world interactions are complex to empirically disentangle the influence of network from other behavioral factors, controlled human experiments provide a means to do so. Rand *et al.* (*71*) showed that theoretical predictions about cooperation thresholds qualitatively match evolutionary outcomes in human groups and that networks with smaller average degree have lower cooperation thresholds and exhibit higher cooperation levels. Similar consistencies of theoretical analysis and realistic observations have been evidenced by many experimental studies (*72*, *73*). By analogy, we expect that network structures with lower critical synergy factors $r^{⋆}$ in our framework should likewise be more conducive to the evolution of cooperation.

For applications to larger population sizes, one challenge is the computational complexity associated with Eqs. 3 to 5. The main linear equations (Eq. 4) of coalescent time $\tau_{\mathrm{ij}}$ are the same as those in ref. (*10*), which can be solved in polynomial time. The additional quantities ${ϒ}_{\mathrm{ij}}$ (Eq. 5) required for PGGs are directly derived from the $\tau_{\mathrm{ij}}$ values and do not lead to a higher computational complexity. We examine six larger empirical networks to demonstrate the computational feasibility of our theory (fig. S6).

Our theory lays the foundation for exploring a large number of extended mechanisms (*6*) in PGGs, such as inertia (*31*, *33*), on general population structures. One can investigate the additional effects of different networks on these mechanisms. One can also study the evolutionary dynamics of PGGs on multilayer (*74*) and dynamic networks (*75*), which were only studied in pairwise games previously. Moreover, one can generalize our results to weighted networks and study the outcomes with arbitrary initial conditions (*12*). The algorithm for group interactions based on second-order neighbors can also be used to study other multiplayer games, including the nonlinear PGGs (*76*–*79*), which bear unknown complexity (*80*, *81*).

## METHODS

### Theoretical conditions for the success of cooperation

Here, we briefly summarize the mathematical derivations of the cooperation condition in PGGs. The system state is denoted by $x=\left(x_{1},x_{2},\ldots ,x_{N}\right)$, where $x_{i}=1$ if agent i cooperates and $x_{i}=0$ if it defects. In this way, we can formalize the payoff calculation on networks. The actual payoff of agent i at system state x, denoted by $f_{i}\left(x\right)$, is expressed as

$$\begin{array}{c} f_{i}\left(x\right)=\frac{1}{G_{i}}\sum_{l\in G_{i}}\left(\frac{r\sum_{\ell \in G_{l}}x_{\ell }c}{G_{l}}-x_{i}c\right)\\ =\frac{1}{1+k_{i}}\left\{\left[\frac{r\left(x_{i}+\sum_{l\in N_{i}}x_{l}\right)c}{k_{i}+1}-x_{i}c\right]+\sum_{l\in N_{i}}\left[\frac{r\left(x_{l}+\sum_{\ell \in N_{l}}x_{\ell }\right)c}{k_{l}+1}-x_{i}c\right]\right\} \end{array}$$ (6)

Agent i plays $G_{i}=1+k_{i}$ games, organized by itself and its $k_{i}$ neighbors $l\in N_{i}$, which form the group $G_{i}=\left\{i\right\}\cup N_{i}$. In the game organized by agent l, there are $G_{l}$ players in the group.

In the weak selection limit (0<δ≪1), the dynamics of strategy evolution almost reduces to the Voter model (*82*), where the marginal role of games does not influence strategy distributions. Therefore, in the previous literature (*10*, *11*) (or Supplementary Note 1), the conditions for the success of cooperation were obtained under neutral drift (δ=0) and remain unchanged for different payoff calculations.

In other words, the key difference in analyzing PGGs is the payoff calculation. We only need to substitute the payoffs of PGGs, i.e., Eq. 6, into the previously obtained cooperation conditions, which (under the PC rule) is

$$\frac{1}{4N^{2}\langle k\rangle }\sum_{i,j\in N}k_{i}p_{\mathrm{ij}}E_{\text{RMC}}^{\circ }\left\{\left(x_{i}-x_{j}\right),\left[f_{i}\left(x\right)-f_{j}\left(x\right)\right]\right\}>0$$ (7)

Applying $f_{i}\left(x\right)$ [$f_{j}\left(x\right)$] of Eq. 6 and considering

$$\tau_{\mathrm{ij}}=\frac{\frac{1}{2}-E_{\text{RMC}}^{\circ }\left[x_{i}x_{j}\right]}{K/4}$$ (8)

we can calculate the condition of Eq. 7 as

$$\begin{array}{c} \sum_{i,j\in N}k_{i}p_{\mathrm{ij}}\left\{\left[\frac{\mathrm{rc}}{{\left(k_{i}+1\right)}^{2}}-c\right]\tau_{\mathrm{ij}}+\frac{\mathrm{rc}}{k_{i}+1}\sum_{l\in N_{i}}\left(\frac{1}{k_{i}+1}+\frac{1}{k_{l}+1}\right)\left(-\tau_{\mathrm{il}}+\tau_{\mathrm{jl}}\right)\right.\\ +\frac{\mathrm{rc}}{k_{i}+1}\sum_{l\in N_{i}}\frac{1}{k_{l}+1}\sum_{\ell \in N_{l}}\left(-\tau_{i\ell }+\tau_{j\ell }\right)-\left[\frac{\mathrm{rc}}{{\left(k_{j}+1\right)}^{2}}-c\right]\left(-\tau_{\mathrm{ij}}\right)\\ -\frac{\mathrm{rc}}{k_{j}+1}\sum_{l\in N_{j}}\left(\frac{1}{k_{j}+1}+\frac{1}{k_{l}+1}\right)\left(-\tau_{\mathrm{il}}+\tau_{\mathrm{jl}}\right)\\ -\frac{\mathrm{rc}}{k_{j}+1}\sum_{l\in N_{j}}\left.\frac{1}{k_{l}+1}\sum_{\ell \in N_{l}}\left(-\tau_{i\ell }+\tau_{j\ell }\right)\right\}>0\\ \Leftrightarrow r>\frac{2\sum_{i,j\in N}k_{i}p_{\mathrm{ij}}\tau_{\mathrm{ij}}}{\sum_{i,j\in N}k_{i}p_{\mathrm{ij}}\left({ϒ}_{\mathrm{ij}}+{ϒ}_{\mathrm{ji}}\right)} \end{array}$$ (9)

which is equivalent to Eq. 3 in the main text, with $\tau_{\mathrm{ij}}$ and ${ϒ}_{\mathrm{ij}}$ obtained through Eqs. 4 and 5, respectively. See Supplementary Note 1 for the meaning of mathematical symbols in Eqs. 7 to 9 and their detailed deductions.

The results under the DB and BD update rules, including those with accumulated payoffs, follow the same idea. We have the cooperation conditions [(eq. S41 for DB and eq. S53 for BD], which are independent of payoff calculations. Applying $f_{i}\left(x\right)$ [$f_{j}\left(x\right)$] of Eq. 6 (or the ones for accumulated payoffs) and their respective $\tau_{\mathrm{ij}}$ values leads to the resultant cooperation conditions, as detailed in Supplementary Note 1.

### Agent-based simulations

We conduct the agent-based simulations using the standard Monte Carlo method. The selection strength δ is between 0.01 (*31*, *40*) and 0.025 (*10*, *83*), which is considered numerically weak. The cost of cooperation c is set to 1 (*20*). In the initial state, there is one random cooperator and N−1 defectors. Each full Monte Carlo step (MCS) contains N elementary time steps where a random focal agent is selected to update the strategy, so that every agent is updated once on average. We allow for up to $4\times 10^{5}$ full MCS, which is theoretically infinite (*10*). If the fraction of cooperators hits a fixation state ($\rho_{C}=1$ or $\rho_{C}=0$), we end the current run and record the result. If a fixation state is not reached within the maximally allowed MCS, we take the actual $\rho_{C}$ at the last step as the result. We repeat the simulations $10^{6}$ to $10^{9}$ times independently under the given game parameters and network, averaging the final fraction of cooperators $\rho_{C}$ in these runs as the actual result of $\rho_{C}$. If the average cooperation fraction $\rho_{C}>1/N$, then evolution favors cooperation.

## Supplementary Materials

### This PDF file includes:

Supplementary Notes 1 to 3 Figs. S1 to S6 References
