# Supplementary material for: Public goods games on any population structure
Source: Sci Adv. 2026 Mar 6;12(10):eaeb1263. doi: 10.1126/sciadv.aeb1263 (PMC12965315; doi:10.1126/sciadv.aeb1263)
Supplement: Supplementary file 1 — Supplementary Notes 1 to 3 Figs. S1 to S6 References [file sciadv.aeb1263_sm.pdf]

Science Advances
AAAS

Supplementary Materials for

Public goods games on any population structure

Chaoqian Wang and Qi Su

Corresponding author: Qi Su, qisu@sjtu.edu.cn

Sci. Adv. **12**, eaeb1263 (2026)
DOI: 10.1126/sciadv.aeb1263

This PDF file includes:

Supplementary Notes 1 to 3
Figs. S1 to S6
References

Supplementary Note 1: Conditions for the success of cooperation in PGGs

1.1 Payoff calculation for PGGs on any network

According to the description in the main text, agents participate in the PGGs organized by themselves and their neighbors, taking the average payoffs obtained in these games as the actual payoff. To formally express this algorithm, we denote the system state (i.e., the strategies of all agents) by $\mathbf{x} = (x_1, x_2, \dots, x_N)$, in a population of size N. If agent i cooperates, $x_i = 1$. If agent i defects, $x_i = 0$. The full cooperation state can be written by $\mathbf{C} = \mathbf{1} = (1, 1, \dots, 1)$ and the full defection state is $\mathbf{D} = \mathbf{0} = (0, 0, \dots, 0)$. The set consisting of all possible system states is denoted by $\mathbf{X}$.

We denote $f_i(\mathbf{x})$ as the average payoff that agent i obtains from the PGGs organized by itself and its neighbors at system state $\mathbf{x}$. Literally, $f_i(\mathbf{x})$ follows the calculation in Eq. (S1):

$$
\begin{aligned}
f_i(\mathbf{x}) &= \frac{1}{G_i} \sum_{l \in \mathscr{G}_i} \left(\frac{r \sum_{\ell \in \mathscr{G}_l} x_\ell c}{G_l} - x_i c \right) \\
&= \frac{1}{1+k_i} \left[\left(\frac{r(x_i + \sum_{l \in \mathscr{N}_i} x_l) c}{k_i + 1} - x_i c \right) + \sum_{l \in \mathscr{N}_i} \left(\frac{r(x_l + \sum_{\ell \in \mathscr{N}_l} x_\ell) c}{k_l + 1} - x_i c \right) \right] \\
&= \left(\frac{rc}{(k_i+1)^2} - c \right) x_i + \frac{rc}{k_i+1} \sum_{l \in \mathscr{N}_i} \left(\frac{1}{k_i+1} + \frac{1}{k_l+1} \right) x_l + \frac{rc}{k_i+1} \sum_{l \in \mathscr{N}_i} \frac{1}{k_l+1} \sum_{\ell \in \mathscr{N}_l} x_\ell .
\end{aligned} \tag{S1}
$$

In the first line, $\mathscr{G}_i = \{i\} \cup \mathscr{N}_i$ is the group containing oneself and its neighbors. In the second line, the former item is the PGG organized by agent i, and the latter item is the PGGs organized by its neighbors $l \in \mathscr{N}_i$. The third line is a simplification of the second line for later use.

1.2 General conditions for the success of cooperation

According to Ref. (*11*), the general condition for the success of cooperation on arbitrary networks is

$$
\mathbb{E}^{\circ}_{\mathrm{RMC}}[\hat{\Delta}'_{\mathrm{sel}}(\mathbf{x})] > 0, \tag{S2}
$$

where, the upper-right corner label $\circ$ of a quantity is to take the value of this quantity at $\delta = 0$ (neutral drift). The upper-right corner label $'$ of a quantity is to calculate the first-order derivative of this quantity with respect to δ at $\delta = 0$.

$\hat{\Delta}_{\mathrm{sel}}(\mathbf{x})$ is the change in the cooperation fraction within an elementary Monte Carlo step (MCS) due to strategy learning (i.e., selection, abbreviated as "sel"), weighted by reproduction numbers, at system state $\mathbf{x}$. The expression of $\hat{\Delta}_{\mathrm{sel}}(\mathbf{x})$ is (*84*)

$$
\hat{\Delta}_{\mathrm{sel}}(\mathbf{x}) = \frac{1}{N} \sum_{i \in \mathscr{N}} x_i (\hat{b}_i(\mathbf{x}) - \hat{d}_i(\mathbf{x})), \tag{S3}
$$

where $\hat{b}_i(\mathbf{x})$ and $\hat{d}_i(\mathbf{x})$ represent the birth and death probabilities of agent i, weighted by reproduction numbers, respectively.

Before weighted by reproduction numbers, $\hat{b}_i(\mathbf{x})$ and $\hat{d}_i(\mathbf{x})$ are given by $b_i(\mathbf{x}) = \sum_{j \in \mathscr{N}} e_{ij}(\mathbf{x})$ and $d_i(\mathbf{x}) = \sum_{j \in \mathscr{N}} e_{ji}(\mathbf{x})$. An agent's strategy reproduces if learned by other agents, or dies if the agent adopts the strategy of others. Here, $e_{ij}(\mathbf{x})$ is the probability that agent i transmits its strategy to agent j, determined by specific strategy update rules.

The birth and death probabilities of agent i weighted by reproduction numbers, $\hat{b}_i(\mathbf{x})$ and $\hat{d}_i(\mathbf{x})$, take the following form:

$$
\hat{b}_i(\mathbf{x}) = \sum_{j \in \mathscr{N}} e_{ij}(\mathbf{x}) v_j, \tag{S4a}
$$

$$
\hat{d}_i(\mathbf{x}) = \sum_{j \in \mathscr{N}} e_{ji}(\mathbf{x}) v_i, \tag{S4b}
$$

where, v_i and v_j represent the reproduction numbers of agents i and j, respectively. The reproduction numbers $\{v_i\}_{i \in \mathscr{N}}$ of all agents in the population are defined based on the fact that under neutral drift, natural selection does not influence the change in strategy proportions ($\hat{\Delta}^{\circ}_{\mathrm{sel}}(\mathbf{x}) = 0$). Additionally, considering normalization, the average reproduction number for each agent is set to 1. Therefore, the following equation holds (*11*):

$$
\hat{d}^{\circ}_i(\mathbf{x}) = \hat{b}^{\circ}_i(\mathbf{x}) \Leftrightarrow \sum_{j \in \mathscr{N}} e^{\circ}_{ji}(\mathbf{x}) v_i = \sum_{j \in \mathscr{N}} e^{\circ}_{ij}(\mathbf{x}) v_j, \tag{S5a}
$$

$$
\sum_{i \in \mathscr{N}} v_i = N. \tag{S5b}
$$

The first line results from $\hat{\Delta}^{\circ}_{\text{sel}}(\mathbf{x}) = 0$, while the second line is for normalization. From Eqs. (S5), the reproduction numbers of all agents can be solved given the network and update rules.

Since there is no expected change in $\hat{\Delta}_{\text{sel}}(\mathbf{x})$ caused by natural selection under neutral drift, we can study the first derivative of $\hat{\Delta}_{\text{sel}}(\mathbf{x})$ with respect to δ at $\delta = 0$ in order to quantitatively analyze $\hat{\Delta}_{\text{sel}}(\mathbf{x})$. Substituting Eqs. (S4) into Eq. (S3) and taking the derivative, we obtain

$$\begin{aligned}\hat{\Delta}'_{\text{sel}}(\mathbf{x}) &= \frac{1}{N}\sum_{i,j\in\mathcal{N}} x_i(e'_{ij}(\mathbf{x})v_j - e'_{ji}(\mathbf{x})v_i) \\ &= \frac{1}{2N}\sum_{i,j\in\mathcal{N}} (x_i - x_j)(e'_{ij}(\mathbf{x})v_j - e'_{ji}(\mathbf{x})v_i). \end{aligned} \tag{S6}$$

Taking the equivalent form in the second line (based on the symmetry between i and j) can facilitate subsequent calculations. At this point, by calculating the strategy reproduction probabilities $\{e_{ij}(\mathbf{x})\}_{i,j\in\mathcal{N},\mathbf{x}\in\mathbf{X}}$ and reproduction numbers $\{v_i\}_{i\in\mathcal{N}}$ under the given update rule, we can obtain the corresponding value of $\hat{\Delta}'_{\text{sel}}(\mathbf{x})$.

Another concept that appears in Eq. (S2) is $\mathbb{E}_{\text{RMC}}[\cdot]$, where RMC stands for the rare-mutation conditional distribution. Before defining RMC, it is necessary to define MSS: the mutation-selection stationary distribution. This distribution describes the system's state when a mutation mechanism is present. The introduction of the mutation mechanism is intended to construct a mathematically tractable, complete Markov chain. Suppose that in each elementary Monte Carlo step, the focal agent mutates with probability u (i.e., mutation): it switches to either cooperation or defection with probability $1/2$ respectively. With the remaining probability $1-u$, the agent update the strategy (i.e., selection) according to the given update rule. The weak mutation limit $u \to 0$ leads to the model we study in the main text, where strategy updates depend solely on the strategy update rule.

$\Pi_{\text{MSS}}(\mathbf{x})$ represents the probability that the system stablizes at state $\mathbf{x}$ under the mutation-selection stationary distribution. The sum of probabilities for all possible stationary states is 1, i.e., $\sum_{\mathbf{x}\in\mathbf{X}} \Pi_{\text{MSS}}(\mathbf{x}) = 1$. Obviously, in the weak mutation limit $u \to 0$, the system has only two stable states: full cooperation ($\mathbf{x} = \mathbf{C}$) and full defection ($\mathbf{x} = \mathbf{D}$): $\Pi_{\text{MSS}}(\mathbf{C}) \to \rho_C/(\rho_C+\rho_D)$, $\Pi_{\text{MSS}}(\mathbf{D}) \to \rho_D/(\rho_C+\rho_D)$ (*85*, *86*). For $\mathbf{x} \notin \{\mathbf{C},\mathbf{D}\}$, $\Pi_{\text{MSS}}(\mathbf{x}) \to 0$.

On this basis, the rare-mutation conditional (RMC) distribution describes the distribution of system states among possible states other than full cooperation and full defection as $u \to 0$. $\Pi_{\text{RMC}}(\mathbf{x})$ represents the probability that the system is in state $\mathbf{x}$ under the RMC distribution, satisfying the normalization condition $\sum_{\mathbf{x}\in\mathbf{X}\setminus\{\mathbf{C},\mathbf{D}\}} \Pi_{\text{RMC}}(\mathbf{x}) = 1$ (note that here $\mathbf{x}$ is restricted to $\mathbf{x} \in \mathbf{X}\setminus\{\mathbf{C},\mathbf{D}\}$, i.e., $\mathbf{x} \notin \{\mathbf{C},\mathbf{D}\}$). By this definition, $\Pi_{\text{RMC}}(\mathbf{x})$ can be derived from $\Pi_{\text{MSS}}(\mathbf{x})$,

$$\Pi_{\text{RMC}}(\mathbf{x}) = \lim_{u\to 0} \frac{\Pi_{\text{MSS}}(\mathbf{x})}{1-\Pi_{\text{MSS}}(\mathbf{C})-\Pi_{\text{MSS}}(\mathbf{D})}. \tag{S7}$$

$\mathbb{E}_{\text{MSS}}[\cdot]$ and $\mathbb{E}_{\text{RMC}}[\cdot]$ represent the expected value under the corresponding distribution, i.e., the sum of the products of all possible state variables in $[\cdot]$ and their respective probabilities. For example, given a function $f(\mathbf{x})$ of the system state $\mathbf{x}$, we have

$$\mathbb{E}_{\text{MSS}}[f(\mathbf{x})] = \sum_{\mathbf{x}\in\mathbf{X}} \Pi_{\text{MSS}}(\mathbf{x})f(\mathbf{x}), \tag{S8}$$

$$\mathbb{E}_{\text{RMC}}[f(\mathbf{x})] = \sum_{\mathbf{x}\in\mathbf{X}\setminus\{\mathbf{C},\mathbf{D}\}} \Pi_{\text{RMC}}(\mathbf{x})f(\mathbf{x}). \tag{S9}$$

Later, we will need the following property: in the limit $u \to 0$, for any agent $i \in \mathcal{N}$, we have $\mathbb{E}^{\circ}_{\text{MSS}}[x_i] = 1/2$ and $\mathbb{E}^{\circ}_{\text{RMC}}[x_i] = 1/2$. To prove, under neutral drift, strategies C and D are indistinguishable and thus interchangeable. From $\rho_C = \rho_D$ and $\rho_C + \rho_D = 1$, we can solve for $\rho_C = \rho_D = 1/2$. Therefore, based on $\Pi_{\text{MSS}}(\mathbf{C}) \to \rho_C/(\rho_C+\rho_D)$ and $\Pi_{\text{MSS}}(\mathbf{D}) \to \rho_D/(\rho_C+\rho_D)$, we have $\Pi_{\text{MSS}}(\mathbf{C}), \Pi_{\text{MSS}}(\mathbf{D}) \to 1/2$. Consequently, $\mathbb{E}^{\circ}_{\text{MSS}}[x_i] = 1/2\times 1 + 1/2\times 0 = 1/2$. Similarly, due to the interchangeability of C and D, we have $\Pi_{\text{RMC}}(\mathbf{x}) = \Pi_{\text{RMC}}(\mathbf{1}-\mathbf{x})$, and we can calculate $\mathbb{E}^{\circ}_{\text{RMC}}[x_i] = (1/2)\sum_{\mathbf{x}\notin\{\mathbf{C},\mathbf{D}\}}(\Pi_{\text{RMC}}(\mathbf{x})x_i + \Pi_{\text{RMC}}(\mathbf{1}-\mathbf{x})(1-x_i)) = (1/2)\sum_{\mathbf{x}\notin\{\mathbf{C},\mathbf{D}\}} \Pi_{\text{RMC}}(\mathbf{x})(x_i + 1 - x_i) = 1/2$.

We define a quantity K,

$$K = \lim_{u\to 0} \frac{u}{1-\Pi_{\text{MSS}}(\mathbf{C})-\Pi_{\text{MSS}}(\mathbf{D})}. \tag{S10}$$

Ref. (*11*) has shown that K exists and is positive.

Later, we will need another property: let $\phi(\mathbf{x})$ be a function that satisfies $\phi(\mathbf{C}) = \phi(\mathbf{D}) = 0$, then we can calculate and find that $\mathbb{E}_{\text{RMC}}[\phi(\mathbf{x})]$ and $\mathbb{E}_{\text{MSS}}[\phi(\mathbf{x})]$ are related by Eq. (S11):

$$\mathbb{E}_{\text{RMC}}[\phi(\mathbf{x})] = \sum_{\mathbf{x}\in\mathbf{X}\setminus\{\mathbf{C},\mathbf{D}\}} \Pi_{\text{RMC}}(\mathbf{x})\phi(\mathbf{x})$$

$$
\begin{aligned}
&= \sum_{\mathbf{x}\in\mathbf{X}\setminus\{\mathbf{C},\mathbf{D}\}} \lim_{u\to 0} \frac{\Pi_{\text{MSS}}(\mathbf{x})}{1-\Pi_{\text{MSS}}(\mathbf{C})-\Pi_{\text{MSS}}(\mathbf{D})}\phi(\mathbf{x}) \\
&= \lim_{u\to 0} \frac{\sum_{\mathbf{x}\in\mathbf{X}\setminus\{\mathbf{C},\mathbf{D}\}} \Pi_{\text{MSS}}(\mathbf{x})\phi(\mathbf{x})}{1-\Pi_{\text{MSS}}(\mathbf{C})-\Pi_{\text{MSS}}(\mathbf{D})} \\
&= \lim_{u\to 0} \frac{\sum_{\mathbf{x}\in\mathbf{X}} \Pi_{\text{MSS}}(\mathbf{x})\phi(\mathbf{x})}{1-\Pi_{\text{MSS}}(\mathbf{C})-\Pi_{\text{MSS}}(\mathbf{D})} \\
&= \lim_{u\to 0} \frac{\mathbb{E}_{\text{MSS}}[\phi(\mathbf{x})]}{1-\Pi_{\text{MSS}}(\mathbf{C})-\Pi_{\text{MSS}}(\mathbf{D})} \\
&= \left(\lim_{u\to 0} \frac{u}{1-\Pi_{\text{MSS}}(\mathbf{C})-\Pi_{\text{MSS}}(\mathbf{D})}\right)\left(\lim_{u\to 0}\frac{\mathbb{E}_{\text{MSS}}[\phi(\mathbf{x})]}{u}\right) \\
&= K\left(\lim_{u\to 0}\frac{\mathbb{E}_{\text{MSS}}[\phi(\mathbf{x})]}{u}\right) \\
&= K\left.\frac{\mathrm{d}\mathbb{E}_{\text{MSS}}[\phi(\mathbf{x})]}{\mathrm{d}u}\right|_{u=0}.
\end{aligned} \tag{S11}
$$

The final step in Eq. (S11) employs L'Hôpital's Rule, which allows for the calculation of the limit when both the numerator and denominator approach zero by taking the derivative of the numerator and denominator separately.

By utilizing the relationship between $\mathbb{E}_{\text{MSS}}[\cdot]$ and $\mathbb{E}_{\text{RMC}}[\cdot]$, we can start from the MSS distribution and, using stability, derive a recurrence relation by strategy updates within an elementary MCS under the given update rule. Then, by taking the weak mutation limit, we can obtain the results needed for the evolutionary dynamics (RMC distribution) as discussed in the main text (see details below).

1.3 Pairwise comparison (PC)

For the PC rule, the probability $e_{ij}(\mathbf{x})$ that agent i transmits its strategy to agent j can be calculated as follows. In each elementary MCS, agent j is selected as the focal agent with probability $1/N$ to update the strategy. Agent i is chosen as the reference by agent j with probability $k_{ji}/k_j = p_{ji}$ (i.e., $k_{ji}/k_j = 1/k_j$ if i neighbors j; otherwise this probability is zero), and agent i's strategy is learned by agent j with the learning probability $W_{j\leftarrow i}(\mathbf{x})$ (defined by Eq. (2) in the main text). That is,

$$
e_{ij}(\mathbf{x}) = \frac{p_{ji}}{N}\times W_{j\leftarrow i}(\mathbf{x}) = \frac{p_{ji}}{N}\times\frac{1}{1+\exp\left(-\delta(f_i(\mathbf{x})-f_j(\mathbf{x}))\right)}. \tag{S12}
$$

Taking $\delta = 0$ in Eq. (S12), we have

$$
e^{\circ}_{ij}(\mathbf{x}) = \frac{p_{ji}}{2N}. \tag{S13}
$$

Taking the derivative of Eq. (S12) with respect to δ at $\delta = 0$, we have

$$
e'_{ij}(\mathbf{x}) = \frac{p_{ji}}{4N}(f_i(\mathbf{x})-f_j(\mathbf{x})). \tag{S14}
$$

Substituting Eq. (S13) into Eqs. (S5), we obtain

$$
\sum_{j\in\mathcal{N}} e^{\circ}_{ji}(\mathbf{x})v_i = \sum_{j\in\mathcal{N}} e^{\circ}_{ij}(\mathbf{x})v_j \Leftrightarrow \frac{v_i}{2N} = \sum_{j\in\mathcal{N}}\frac{p_{ji}}{2N}v_j, \tag{S15a}
$$

$$
\sum_{i\in\mathcal{N}} v_i = N. \tag{S15b}
$$

Given that $p_{ji} = k_{ji}/k_j$, the solution to Eqs. (S15) is $v_i = k_i/\langle k\rangle$ for $i\in\mathcal{N}$, where $\langle k\rangle = (\sum_{j\in\mathcal{N}} k_j)/N$ represents the average degree of all nodes on the network.

Substituting $v_i = k_i/\langle k\rangle$ and Eq. (S14) into Eq. (S6), we can calculate $\hat{\Delta}'_{\text{sel}}(\mathbf{x})$ under the PC rule:

$$
\begin{aligned}
\hat{\Delta}'_{\text{sel}}(\mathbf{x}) &= \frac{1}{2N}\sum_{i,j\in\mathcal{N}}(x_i-x_j)(e'_{ij}(\mathbf{x})v_j - e'_{ji}(\mathbf{x})v_i) \\
&= \frac{1}{2N}\sum_{i,j\in\mathcal{N}}(x_i-x_j)\left(\frac{p_{ji}}{4N}(f_i(\mathbf{x})-f_j(\mathbf{x}))\frac{k_j}{\langle k\rangle} - \frac{p_{ij}}{4N}(f_j(\mathbf{x})-f_i(\mathbf{x}))\frac{k_i}{\langle k\rangle}\right)
\end{aligned}
$$

$$= \frac{1}{2N} \sum_{i,j \in \mathcal{N}} (x_i - x_j) \frac{k_{ij}}{4N\langle k \rangle} (f_i(\mathbf{x}) - f_j(\mathbf{x}) - f_j(\mathbf{x}) + f_i(\mathbf{x}))$$
$$= \frac{1}{4N^2\langle k \rangle} \sum_{i,j \in \mathcal{N}} (x_i - x_j) k_i p_{ij} (f_i(\mathbf{x}) - f_j(\mathbf{x})). \tag{S16}$$

Substituting Eq. (S16) into Eq. (S2), we obtain the condition for the success of cooperation under the PC rule:

$$\mathbb{E}^{\circ}_{\mathrm{RMC}}[\hat{\Delta}'_{\mathrm{sel}}(\mathbf{x})] > 0 \Leftrightarrow \frac{1}{4N^2\langle k \rangle} \sum_{i,j \in \mathcal{N}} k_i p_{ij} \mathbb{E}^{\circ}_{\mathrm{RMC}}[(x_i - x_j)(f_i(\mathbf{x}) - f_j(\mathbf{x}))] > 0. \tag{S17}$$

Note that quantities such as N, k_i, and p_{ij} are input parameters with constant expected values. Therefore, they can be factored out of $\mathbb{E}^{\circ}_{\mathrm{RMC}}[\cdot]$.

We first calculate $\mathbb{E}^{\circ}_{\mathrm{RMC}}[(x_i - x_j)(f_i(\mathbf{x}) - f_j(\mathbf{x}))]$ in Eq. (S17). Inserting the payoffs in PGGs, $f_i(\mathbf{x})$ and $f_j(\mathbf{x})$, by using Eq. (S1) and notice that r and c are also input parameters that remain constant, we have

$$\begin{aligned}
&\mathbb{E}^{\circ}_{\mathrm{RMC}}[(x_i - x_j)(f_i(\mathbf{x}) - f_j(\mathbf{x}))] \\
&= \mathbb{E}^{\circ}_{\mathrm{RMC}}\Bigg[\left(\frac{rc}{(k_i+1)^2} - c \right) (x_i^2 - x_i x_j) + \frac{rc}{k_i+1} \sum_{l \in \mathcal{N}_i} \left(\frac{1}{k_i+1} + \frac{1}{k_l+1} \right) (x_i x_l - x_j x_l) \\
&\quad + \frac{rc}{k_i+1} \sum_{l \in \mathcal{N}_i} \frac{1}{k_l+1} \sum_{\ell \in \mathcal{N}_l} (x_i x_\ell - x_j x_\ell) - \left(\frac{rc}{(k_j+1)^2} - c \right) (x_i x_j - x_j^2) \\
&\quad - \frac{rc}{k_j+1} \sum_{l \in \mathcal{N}_j} \left(\frac{1}{k_j+1} + \frac{1}{k_l+1} \right) (x_i x_l - x_j x_l) - \frac{rc}{k_j+1} \sum_{l \in \mathcal{N}_j} \frac{1}{k_l+1} \sum_{\ell \in \mathcal{N}_l} (x_i x_\ell - x_j x_\ell) \Bigg] \\
&= \left(\frac{rc}{(k_i+1)^2} - c \right) (\mathbb{E}^{\circ}_{\mathrm{RMC}}[x_i^2] - \mathbb{E}^{\circ}_{\mathrm{RMC}}[x_i x_j]) + \frac{rc}{k_i+1} \sum_{l \in \mathcal{N}_i} \left(\frac{1}{k_i+1} + \frac{1}{k_l+1} \right) (\mathbb{E}^{\circ}_{\mathrm{RMC}}[x_i x_l] - \mathbb{E}^{\circ}_{\mathrm{RMC}}[x_j x_l]) \\
&\quad + \frac{rc}{k_i+1} \sum_{l \in \mathcal{N}_i} \frac{1}{k_l+1} \sum_{\ell \in \mathcal{N}_l} (\mathbb{E}^{\circ}_{\mathrm{RMC}}[x_i x_\ell] - \mathbb{E}^{\circ}_{\mathrm{RMC}}[x_j x_\ell]) - \left(\frac{rc}{(k_j+1)^2} - c \right) (\mathbb{E}^{\circ}_{\mathrm{RMC}}[x_i x_j] - \mathbb{E}^{\circ}_{\mathrm{RMC}}[x_j^2]) \\
&\quad - \frac{rc}{k_j+1} \sum_{l \in \mathcal{N}_j} \left(\frac{1}{k_j+1} + \frac{1}{k_l+1} \right) (\mathbb{E}^{\circ}_{\mathrm{RMC}}[x_i x_l] - \mathbb{E}^{\circ}_{\mathrm{RMC}}[x_j x_l]) \\
&\quad - \frac{rc}{k_j+1} \sum_{l \in \mathcal{N}_j} \frac{1}{k_l+1} \sum_{\ell \in \mathcal{N}_l} (\mathbb{E}^{\circ}_{\mathrm{RMC}}[x_i x_\ell] - \mathbb{E}^{\circ}_{\mathrm{RMC}}[x_j x_\ell]) .
\end{aligned} \tag{S18}$$

Since $x_i \in \{0,1\}$, we have $x_i^2 = x_i$, and thus $\mathbb{E}^{\circ}_{\mathrm{RMC}}[x_i^2] = \mathbb{E}^{\circ}_{\mathrm{RMC}}[x_i] = 1/2$ for $i \in \mathcal{N}$. The remaining work is to calculate $\mathbb{E}^{\circ}_{\mathrm{RMC}}[x_i x_j]$ for all $i, j \in \mathcal{N}$.

We begin with the MSS distribution. Since the MSS distribution is stationary, the expected system's state remains unchanged after strategy updates. We aim to derive a recurrence relation by working through the strategy update within an elementary MCS. For convenience of calculation, we study $\mathbb{E}^{\circ}_{\mathrm{MSS}}[(x_i - 1/2)(x_j - 1/2)]$, which has a useful property $\mathbb{E}^{\circ}_{\mathrm{MSS}}[x_i - 1/2] = 0$ due to $\mathbb{E}^{\circ}_{\mathrm{MSS}}[x_i] = 1/2$.

Integrating the mutation mechanism described in Section 1.2, the possible events that happen within an elementary MCS can be classified into the following categories based on their impact on x_i or x_j.

- Agent i is selected as the focal agent with probability $1/N$ to update its strategy:

  (i) The focal agent i mutates with probability u, becoming cooperation ($x_i \leftarrow 1$) with probability $1/2$, or defection ($x_i \leftarrow 0$) with probability $1/2$;

  (ii) Agent i updates its strategy under the PC rule with probability $1-u$. With probability p_{il}, agent i chooses reference agent l ($l \in \mathcal{N}$), learning l's strategy, $x_i \leftarrow x_l$, with probability $W^{\circ}_{i \leftarrow l}(\mathbf{x}) = 1/2$ (note that we are discussing neutral drift now), or keeping the current strategy x_i unchanged with probability $1 - W^{\circ}_{i \leftarrow l}(\mathbf{x}) = 1/2$. The probabilities summarized here are consistent with the strategy transmission probability $e^{\circ}_{li} = p_{il}/(2N)$ in Eq. (S13), but the probability $1/N$ to select focal agent i is not repeatedly considered.

- Similarly, agent j is selected as the focal agent with probability $1/N$ to update its strategy:

(i) The focal agent j mutates with probability u, becoming cooperation ($x_j \leftarrow 1$) with probability $1/2$, or defection ($x_j \leftarrow 0$) with probability $1/2$;

(ii) Agent j updates its strategy under the PC rule with probability $1-u$. With probability p_{jl}, agent j chooses reference agent l ($l \in \mathcal{N}$), learning l's strategy, $x_j \leftarrow x_l$, with probability $W^{\circ}_{j\leftarrow l}(\mathbf{x}) = 1/2$, or keeping the current strategy x_j unchanged with probability $1 - W^{\circ}_{j\leftarrow l}(\mathbf{x}) = 1/2$.

- The focal agent is one of the remaining $N-2$ agents other than i and j, with probability $1/N$. Since only the focal agent's strategy may update, both x_i and x_j remain unchanged.

Combining all the above possibilities of an elementary MCS, we can obtain the following recurrence relation under the MSS distribution:

$$
\begin{aligned}
&\mathbb{E}^{\circ}_{\text{MSS}}[(x_i-1/2)(x_j-1/2)] \\
=&\frac{1}{N}\Bigg\{u\left(\frac{1}{2}\mathbb{E}^{\circ}_{\text{MSS}}[(1-1/2)(x_j-1/2)]+\frac{1}{2}\mathbb{E}^{\circ}_{\text{MSS}}[(0-1/2)(x_j-1/2)]\right) \\
&+(1-u)\sum_{l\in\mathcal{N}} p_{il}\Big(W^{\circ}_{i\leftarrow l}(\mathbf{x})\mathbb{E}^{\circ}_{\text{MSS}}[(x_l-1/2)(x_j-1/2)]+(1-W^{\circ}_{i\leftarrow l}(\mathbf{x}))\mathbb{E}^{\circ}_{\text{MSS}}[(x_i-1/2)(x_j-1/2)]\Big)\Bigg\} \\
&+\frac{1}{N}\Bigg\{u\left(\frac{1}{2}\mathbb{E}^{\circ}_{\text{MSS}}[(x_i-1/2)(1-1/2)]+\frac{1}{2}\mathbb{E}^{\circ}_{\text{MSS}}[(x_i-1/2)(0-1/2)]\right) \\
&+(1-u)\sum_{l\in\mathcal{N}} p_{jl}\Big(W^{\circ}_{j\leftarrow l}(\mathbf{x})\mathbb{E}^{\circ}_{\text{MSS}}[(x_i-1/2)(x_l-1/2)]+(1-W^{\circ}_{j\leftarrow l}(\mathbf{x}))\mathbb{E}^{\circ}_{\text{MSS}}[(x_i-1/2)(x_j-1/2)]\Big)\Bigg\} \\
&+(N-2)\frac{1}{N}\mathbb{E}^{\circ}_{\text{MSS}}[(x_i-1/2)(x_j-1/2)] \\
=&\frac{1}{N}\Bigg\{0+(1-u)\sum_{l\in\mathcal{N}}\frac{p_{il}}{2}\Big(\mathbb{E}^{\circ}_{\text{MSS}}[(x_l-1/2)(x_j-1/2)]+\mathbb{E}^{\circ}_{\text{MSS}}[(x_i-1/2)(x_j-1/2)]\Big)\Bigg\} \\
&+\frac{1}{N}\Bigg\{0+(1-u)\sum_{l\in\mathcal{N}}\frac{p_{jl}}{2}\Big(\mathbb{E}^{\circ}_{\text{MSS}}[(x_i-1/2)(x_l-1/2)]+\mathbb{E}^{\circ}_{\text{MSS}}[(x_i-1/2)(x_j-1/2)]\Big)\Bigg\} \\
&+(N-2)\frac{1}{N}\mathbb{E}^{\circ}_{\text{MSS}}[(x_i-1/2)(x_j-1/2)].
\end{aligned}
\tag{S19}
$$

We integrate $\mathbb{E}^{\circ}_{\text{MSS}}[(x_i-1/2)(x_j-1/2)]$ into the left-hand side and denote $\underline{x}_i = x_i - 1/2$ for convenience. Then, we have

$$
\mathbb{E}^{\circ}_{\text{MSS}}[\underline{x}_i\underline{x}_j] = \frac{1-u}{2}\left(\sum_{l\in\mathcal{N}} p_{il}\mathbb{E}^{\circ}_{\text{MSS}}[\underline{x}_l\underline{x}_j] + \sum_{l\in\mathcal{N}} p_{jl}\mathbb{E}^{\circ}_{\text{MSS}}[\underline{x}_i\underline{x}_l]\right). \tag{S20}
$$

We define variables $\phi_{ij}(\mathbf{x})$,

$$
\phi_{ij}(\mathbf{x}) = \underline{x}_i\underline{x}_j - \frac{1}{2}\left(\sum_{l\in\mathcal{N}} p_{il}\underline{x}_l\underline{x}_j + \sum_{l\in\mathcal{N}} p_{jl}\underline{x}_i\underline{x}_l\right), \tag{S21}
$$

which satisfy the properties $\phi_{ij}(\mathbf{C}) = 1/4 - 1/2 \times (1/4 + 1/4) = 0$ and similarly, $\phi_{ij}(\mathbf{D}) = 0$. Therefore, $\phi_{ij}(\mathbf{x})$ can be used to relate the MSS and RMC distributions through Eq. (S11) as $u \to 0$.

We first calculate $\mathbb{E}^{\circ}_{\text{MSS}}[\phi_{ij}(\mathbf{x})]$. Writing down the expected value of Eq. (S21) and using Eq. (S20), we have

$$
\begin{aligned}
\mathbb{E}^{\circ}_{\text{MSS}}[\phi_{ij}(\mathbf{x})] &= \mathbb{E}^{\circ}_{\text{MSS}}[\underline{x}_i\underline{x}_j] - \frac{1}{2}\left(\sum_{l\in\mathcal{N}} p_{il}\mathbb{E}^{\circ}_{\text{MSS}}[\underline{x}_l\underline{x}_j] + \sum_{l\in\mathcal{N}} p_{jl}\mathbb{E}^{\circ}_{\text{MSS}}[\underline{x}_i\underline{x}_l]\right) \\
&= \mathbb{E}^{\circ}_{\text{MSS}}[\underline{x}_i\underline{x}_j] - \frac{1}{1-u}\mathbb{E}^{\circ}_{\text{MSS}}[\underline{x}_i\underline{x}_j] \\
&= -\frac{u}{1-u}\mathbb{E}^{\circ}_{\text{MSS}}[\underline{x}_i\underline{x}_j].
\end{aligned}
\tag{S22}
$$

According to Eq. (S11), we can calculate $\mathbb{E}^{\circ}_{\mathrm{RMC}}[\phi_{ij}(\mathbf{x})]$ from $\mathbb{E}^{\circ}_{\mathrm{MSS}}[\phi_{ij}(\mathbf{x})]$,

$$
\begin{aligned}
\mathbb{E}^{\circ}_{\mathrm{RMC}}[\phi_{ij}(\mathbf{x})] &= K \left.\frac{\mathrm{d}\mathbb{E}^{\circ}_{\mathrm{MSS}}[\phi_{ij}(\mathbf{x})]}{\mathrm{d}u}\right|_{u=0} \\
&= K \left.\frac{\mathrm{d}}{\mathrm{d}u}\right|_{u=0} \left(-\frac{u}{1-u}\mathbb{E}^{\circ}_{\mathrm{MSS}}[\underline{x_i}\underline{x_j}]\right) \\
&= K \left(-\left.\mathbb{E}^{\circ}_{\mathrm{MSS}}[\underline{x_i}\underline{x_j}]\right|_{u=0} + 0\right) \\
&= -K\left(\frac{1}{2}\times(1-\frac{1}{2})(1-\frac{1}{2}) + \frac{1}{2}\times(0-\frac{1}{2})(0-\frac{1}{2})\right) \\
&= -\frac{K}{4}.
\end{aligned} \tag{S23}
$$

In the second-to-last step, we recalled that as $u \to 0$, there are only two stationary states under MSS, $\mathbf{x} = \mathbf{1}$ or $\mathbf{x} = \mathbf{0}$, each with probability $1/2$ under neutral drift.

On the other hand, we write down the expected value of Eq. (S21) under the RMC distribution and obtain another expression of $\mathbb{E}^{\circ}_{\mathrm{RMC}}[\phi_{ij}(\mathbf{x})]$:

$$
\mathbb{E}^{\circ}_{\mathrm{RMC}}[\phi_{ij}(\mathbf{x})] = \mathbb{E}^{\circ}_{\mathrm{RMC}}[\underline{x_i}\underline{x_j}] - \frac{1}{2}\left(\sum_{l\in\mathcal{N}} p_{il}\mathbb{E}^{\circ}_{\mathrm{RMC}}[\underline{x_l}\underline{x_j}] + \sum_{l\in\mathcal{N}} p_{jl}\mathbb{E}^{\circ}_{\mathrm{RMC}}[\underline{x_i}\underline{x_l}]\right). \tag{S24}
$$

Substituting $\mathbb{E}^{\circ}_{\mathrm{RMC}}[\phi_{ij}(\mathbf{x})] = -K/4$ (Eq. (S23)) into Eq. (S24), we have

$$
\mathbb{E}^{\circ}_{\mathrm{RMC}}[\underline{x_i}\underline{x_j}] = \frac{1}{2}\left(\sum_{l\in\mathcal{N}} p_{il}\mathbb{E}^{\circ}_{\mathrm{RMC}}[\underline{x_l}\underline{x_j}] + \sum_{l\in\mathcal{N}} p_{jl}\mathbb{E}^{\circ}_{\mathrm{RMC}}[\underline{x_i}\underline{x_l}]\right) - \frac{K}{4}. \tag{S25}
$$

We define variables τ_{ij} for $i, j \in \mathcal{N}$,

$$
\tau_{ij} = \frac{\frac{1}{2} - \mathbb{E}^{\circ}_{\mathrm{RMC}}[x_i x_j]}{K/4}. \tag{S26}
$$

Obviously, $\tau_{ii} = 0$ when $i = j$, because $\mathbb{E}^{\circ}_{\mathrm{RMC}}[x_i^2] = 1/2$. Also, $\tau_{ij} = \tau_{ji}$, because $\mathbb{E}^{\circ}_{\mathrm{RMC}}[x_i x_j] = \mathbb{E}^{\circ}_{\mathrm{RMC}}[x_j x_i]$.

When $i \neq j$, we can solve for the values of τ_{ij} by the recurrence relation. We know that $\mathbb{E}^{\circ}_{\mathrm{RMC}}[x_i x_j]$ and $\mathbb{E}^{\circ}_{\mathrm{RMC}}[\underline{x_i}\underline{x_j}]$ have the following relation:

$$
\begin{aligned}
\mathbb{E}^{\circ}_{\mathrm{RMC}}[\underline{x_i}\underline{x_j}] &= \mathbb{E}^{\circ}_{\mathrm{RMC}}[(x_i - 1/2)(x_j - 1/2)] \\
&= \mathbb{E}^{\circ}_{\mathrm{RMC}}[x_i x_j] - \frac{1}{2}\mathbb{E}^{\circ}_{\mathrm{RMC}}[x_i] - \frac{1}{2}\mathbb{E}^{\circ}_{\mathrm{RMC}}[x_j] + \frac{1}{4} \\
&= \mathbb{E}^{\circ}_{\mathrm{RMC}}[x_i x_j] - \frac{1}{4},
\end{aligned} \tag{S27}
$$

and therefore, Eq. (S26) can be written as

$$
\tau_{ij} = \frac{\frac{1}{2} - \left(\mathbb{E}^{\circ}_{\mathrm{RMC}}[\underline{x_i}\underline{x_j}] + \frac{1}{4}\right)}{K/4} = \frac{1 - 4\mathbb{E}^{\circ}_{\mathrm{RMC}}[\underline{x_i}\underline{x_j}]}{K} \tag{S28}
$$

or

$$
\mathbb{E}^{\circ}_{\mathrm{RMC}}[\underline{x_i}\underline{x_j}] = \frac{1 - K\tau_{ij}}{4}. \tag{S29}
$$

Substituting Eq. (S29) into Eq. (S25), we obtain the recurrence relation of τ_{ij}:

$$
\frac{1 - K\tau_{ij}}{4} = \frac{1}{2}\left(\sum_{l\in\mathcal{N}} p_{il}\frac{1 - K\tau_{lj}}{4} + \sum_{l\in\mathcal{N}} p_{jl}\frac{1 - K\tau_{il}}{4}\right) - \frac{K}{4}
$$

$$\Leftrightarrow \tau_{ij} = 1 + \frac{1}{2}\left(\sum_{l\in\mathcal{N}} p_{il}\tau_{lj} + \sum_{l\in\mathcal{N}} p_{jl}\tau_{il}\right). \tag{S30}$$

The recurrence relation Eq. (S30), together with $\tau_{ii} = 0$, form a system of linear equations, through which all τ_{ij} values ($i, j \in \mathcal{N}$) can be determined on a given network.

Back to the halfway calculation in Eq. (S18) of the cooperation success condition. Substituting all $\mathbb{E}^{\circ}_{\mathrm{RMC}}[x_i x_j]$ into Eq. (S18) with computable τ_{ij} using Eq. (S26), and then substituting the result into Eq. (S17), where the positive factors $K/4$ and $1/(4N^2\langle k\rangle)$ can be canceled out, we arrive at the following condition for the success of cooperation:

$$\begin{aligned}
&\mathbb{E}^{\circ}_{\mathrm{RMC}}[\hat{\Delta}'_{\mathrm{sel}}(\mathbf{x})] > 0\\
\Leftrightarrow &\sum_{i,j\in\mathcal{N}} k_i p_{ij}\Bigg\{\left(\frac{rc}{(k_i+1)^2} - c\right)\tau_{ij} + \frac{rc}{k_i+1}\sum_{l\in\mathcal{N}_i}\left(\frac{1}{k_i+1}+\frac{1}{k_l+1}\right)(-\tau_{il}+\tau_{jl})\\
&+\frac{rc}{k_i+1}\sum_{l\in\mathcal{N}_i}\frac{1}{k_l+1}\sum_{\ell\in\mathcal{N}_l}(-\tau_{i\ell}+\tau_{j\ell}) - \left(\frac{rc}{(k_j+1)^2}-c\right)(-\tau_{ij})\\
&-\frac{rc}{k_j+1}\sum_{l\in\mathcal{N}_j}\left(\frac{1}{k_j+1}+\frac{1}{k_l+1}\right)(-\tau_{il}+\tau_{jl}) - \frac{rc}{k_j+1}\sum_{l\in\mathcal{N}_j}\frac{1}{k_l+1}\sum_{\ell\in\mathcal{N}_l}(-\tau_{i\ell}+\tau_{j\ell})\Bigg\} > 0\\
\Leftrightarrow\ & r > \frac{2\sum_{i,j\in\mathcal{N}} k_i p_{ij}\tau_{ij}}{\sum_{i,j\in\mathcal{N}} k_i p_{ij}(\Upsilon_{ij}+\Upsilon_{ji})},
\end{aligned} \tag{S31}$$

where Υ_{ij} are defined by (equivalent to Eq. (5) in the main text)

$$\Upsilon_{ij} = \frac{1}{k_i+1}\left(\frac{\tau_{ij} + k_i\sum_{l\in\mathcal{N}} p_{il}(\tau_{jl}-\tau_{il})}{k_i+1} + k_i\sum_{l\in\mathcal{N}} p_{il}\frac{(\tau_{jl}-\tau_{il}) + k_l\sum_{\ell\in\mathcal{N}} p_{l\ell}(\tau_{j\ell}-\tau_{i\ell})}{k_l+1}\right). \tag{S32}$$

And according to the previous discussion, τ_{ij} can be solved by the following system of linear equations (equivalent to Eq. (4) in the main text):

$$\begin{cases}\tau_{ij} = 1 + \dfrac{1}{2}\displaystyle\sum_{l\in\mathcal{N}}(p_{il}\tau_{jl} + p_{jl}\tau_{il}), & \text{if } j \neq i,\\ \tau_{ij} = 0, & \text{if } j = i.\end{cases} \tag{S33}$$

Finally, although usually $\Upsilon_{ij} \neq \Upsilon_{ji}$, we can still infer that $\sum_{i,j\in\mathcal{N}} k_i p_{ij}\Upsilon_{ji} = \sum_{i,j\in\mathcal{N}} k_{ij}\Upsilon_{ji} = \sum_{j,i\in\mathcal{N}} k_{ji}\Upsilon_{ij} = \sum_{j,i\in\mathcal{N}} k_{ij}\Upsilon_{ij} = \sum_{j,i\in\mathcal{N}} k_i p_{ij}\Upsilon_{ij}$, such that $\sum_{i,j\in\mathcal{N}} k_i p_{ij}(\Upsilon_{ij}+\Upsilon_{ji}) = 2\sum_{i,j\in\mathcal{N}} k_i p_{ij}\Upsilon_{ij}$. Therefore, $\sum_{i,j\in\mathcal{N}} k_i p_{ij}(\Upsilon_{ij}+\Upsilon_{ji}) = 2\sum_{i,j\in\mathcal{N}} k_i p_{ij}\Upsilon_{ij}$. As a result, Eq. (S31) can be further simplified as

$$r > \frac{\sum_{i,j\in\mathcal{N}} k_i p_{ij}\tau_{ij}}{\sum_{i,j\in\mathcal{N}} k_i p_{ij}\Upsilon_{ij}}. \tag{S34}$$

The right-hand side is the $r^{\star}$ value under the PC rule, as mentioned in the main text.

1.4 Death-birth (DB)

For the DB rule, the probability $e_{ij}(\mathbf{x})$ that agent i transmits its strategy to agent j can be calculated as follows. In each elementary MCS, agent j is selected as the focal agent with probability $1/N$ to update the strategy. Agent j's strategy "dies", and agent i's strategy occupies the vacant position with probability $W_{j\leftarrow i}(\mathbf{x})$, which is proportional to its fitness among j's neighbors (see Eq. (S35)). That is,

$$e_{ij}(\mathbf{x}) = \frac{1}{N}\times W_{j\leftarrow i}(\mathbf{x}) = \frac{1}{N}\times\frac{k_{ji}F_i(\mathbf{x})}{\sum_{l\in\mathcal{N}} k_{jl}F_l(\mathbf{x})}. \tag{S35}$$

Taking $\delta = 0$ in Eq. (S35), we have

$$e^{\circ}_{ij}(\mathbf{x}) = \frac{k_{ji}}{Nk_j} = \frac{p_{ji}}{N}. \tag{S36}$$

Taking the derivative of Eq. (S35) with respect to δ at $\delta = 0$, we have

$$e'_{ij}(\mathbf{x}) = \frac{1}{N}\frac{k_{ji}f_i(\mathbf{x})k_j - k_{ji}\sum_{l\in\mathcal{N}} k_{jl}f_l(\mathbf{x})}{k_j^2} = \frac{p_{ji}}{N}\left(f_i(\mathbf{x}) - \sum_{l\in\mathcal{N}} p_{jl}f_l(\mathbf{x})\right). \tag{S37}$$

Substituting Eq. (S36) into Eqs. (S5), we obtain

$$\sum_{j\in\mathcal{N}} e^{\circ}_{ji}(\mathbf{x})v_i = \sum_{j\in\mathcal{N}} e^{\circ}_{ij}(\mathbf{x})v_j \Leftrightarrow \frac{v_i}{N} = \sum_{j\in\mathcal{N}} \frac{p_{ji}}{N}v_j, \tag{S38a}$$

$$\sum_{i\in\mathcal{N}} v_i = N. \tag{S38b}$$

Similar to the PC rule, the solution to Eqs. (S38) is also $v_i = k_i/\langle k\rangle$ for $i \in \mathcal{N}$, where $\langle k\rangle = (\sum_{j\in\mathcal{N}} k_j)/N$ represents the average degree of all nodes.

Substituting $v_i = k_i/\langle k\rangle$ and Eq. (S37) into Eq. (S6), we can calculate $\hat{\Delta}'_{\text{sel}}(\mathbf{x})$ under the DB rule. We start from the first line in Eq. (S6),

$$\begin{aligned}
\frac{1}{N}\sum_{i,j\in\mathcal{N}} x_i(e'_{ij}(\mathbf{x})v_j - e'_{ji}(\mathbf{x})v_i) &= \frac{1}{N}\sum_{i,j\in\mathcal{N}} x_i\left(\frac{p_{ji}}{N}\left(f_i(\mathbf{x}) - \sum_{l\in\mathcal{N}} p_{jl}f_l(\mathbf{x})\right)\frac{k_j}{\langle k\rangle} - \frac{p_{ij}}{N}\left(f_j(\mathbf{x}) - \sum_{l\in\mathcal{N}} p_{il}f_l(\mathbf{x})\right)\frac{k_i}{\langle k\rangle}\right)\\
&= \frac{1}{N}\sum_{i,j\in\mathcal{N}} x_i\frac{k_ip_{ij}}{N\langle k\rangle}\left(f_i(\mathbf{x}) - \sum_{l\in\mathcal{N}} p_{jl}f_l(\mathbf{x}) - f_j(\mathbf{x}) + \sum_{l\in\mathcal{N}} p_{il}f_l(\mathbf{x})\right)\\
&= \frac{1}{N}\sum_{i\in\mathcal{N}} x_i\frac{k_i}{N\langle k\rangle}\left(f_i(\mathbf{x}) - \sum_{j,l\in\mathcal{N}} p_{ij}p_{jl}f_l(\mathbf{x}) - \sum_{j\in\mathcal{N}} p_{ij}f_j(\mathbf{x}) + \sum_{l\in\mathcal{N}} p_{il}f_l(\mathbf{x})\right)\\
&= \frac{1}{N}\sum_{i\in\mathcal{N}} x_i\frac{k_i}{N\langle k\rangle}\left(f_i(\mathbf{x}) - \sum_{l\in\mathcal{N}} p^{(2)}_{il}f_l(\mathbf{x})\right)\\
&= \frac{1}{N}\sum_{i,j\in\mathcal{N}} x_i\frac{k_ip^{(2)}_{ij}}{N\langle k\rangle}\left(f_i(\mathbf{x}) - f_j(\mathbf{x})\right),
\end{aligned} \tag{S39}$$

where $p^{(2)}_{ij}$ is defined as $\sum_{l\in\mathcal{N}} p_{il}p_{lj}$. Then, by comparing Eq. (S39) and Eq. (S6), we know that

$$\begin{aligned}
\hat{\Delta}'_{\text{sel}}(\mathbf{x}) &= \frac{1}{N}\sum_{i,j\in\mathcal{N}} x_i(e'_{ij}(\mathbf{x})v_j - e'_{ji}(\mathbf{x})v_i)\\
&= \frac{1}{2N}\sum_{i,j\in\mathcal{N}} (x_i - x_j)(e'_{ij}(\mathbf{x})v_j - e'_{ji}(\mathbf{x})v_i)\\
&= \frac{1}{2N^2\langle k\rangle}\sum_{i,j\in\mathcal{N}} (x_i - x_j)k_ip^{(2)}_{ij}(f_i(\mathbf{x}) - f_j(\mathbf{x})).
\end{aligned} \tag{S40}$$

Substituting Eq. (S40) into Eq. (S2), we obtain the condition for the success of cooperation under the DB rule:

$$\mathbb{E}^{\circ}_{\text{RMC}}[\hat{\Delta}'_{\text{sel}}(\mathbf{x})] > 0 \Leftrightarrow \frac{1}{2N^2\langle k\rangle}\sum_{i,j\in\mathcal{N}} k_ip^{(2)}_{ij}\mathbb{E}^{\circ}_{\text{RMC}}[(x_i - x_j)(f_i(\mathbf{x}) - f_j(\mathbf{x}))] > 0. \tag{S41}$$

Similarly, we first calculate $\mathbb{E}^{\circ}_{\text{RMC}}[(x_i - x_j)(f_i(\mathbf{x}) - f_j(\mathbf{x}))]$ in Eq. (S41), which is completely the same as the one under the PC rule (see Eq. (S18) for the same result).

The remaining work is to calculate $\mathbb{E}^{\circ}_{\text{RMC}}[x_ix_j]$ for all $i, j \in \mathcal{N}$ under the DB rule. Similarly, we begin with the MSS distribution and aim to derive a recurrence relation by working through the strategy update within an elementary MCS. Under the DB rule, the possible events that happen within an elementary MCS can be classified into the following categories based on their impact on x_i or x_j.

- Agent i is selected as the focal agent with probability $1/N$ to update its strategy:

  (i) The focal agent i mutates with probability u, becoming cooperation ($x_i \leftarrow 1$) with probability $1/2$, or defection ($x_i \leftarrow 0$) with probability $1/2$;

(ii) Agent i learns the strategy of a neighbor under the DB rule with probability $1-u$. With probability $W^{\circ}_{i\leftarrow l}(\mathbf{x}) = p_{il}$, agent i learns the strategy of agent l, $x_i \leftarrow x_l$. Note that under the DB rule, $\sum_{l\in\mathcal{N}} W^{\circ}_{i\leftarrow l}(\mathbf{x}) = 1$; the focal agent cannot keep its own strategy.

- Similarly, agent j is selected as the focal agent with probability $1/N$ to update its strategy:

  (i) The focal agent j mutates with probability u, becoming cooperation ($x_j \leftarrow 1$) with probability $1/2$, or defection ($x_j \leftarrow 0$) with probability $1/2$;

  (ii) Agent j learns the strategy of a neighbor under the DB rule with probability $1-u$. With probability $W^{\circ}_{j\leftarrow l}(\mathbf{x}) = p_{jl}$, agent j learns the strategy of agent l, $x_j \leftarrow x_l$.

- The focal agent is one of the remaining $N-2$ agents other than i and j, with probability $1/N$. Since only the focal agent's strategy can update, both x_i and x_j remain unchanged.

Combining all the above possibilities of an elementary MCS, we can obtain the following recurrence relation under the MSS distribution:

$$
\begin{aligned}
&\mathbb{E}^{\circ}_{\text{MSS}}[(x_i-1/2)(x_j-1/2)] \\
=&\frac{1}{N}\left\{u\left(\frac{1}{2}\mathbb{E}^{\circ}_{\text{MSS}}[(1-1/2)(x_j-1/2)]+\frac{1}{2}\mathbb{E}^{\circ}_{\text{MSS}}[(0-1/2)(x_j-1/2)]\right)\right. \\
&\left.+(1-u)\sum_{l\in\mathcal{N}} W^{\circ}_{i\leftarrow l}(\mathbf{x})\mathbb{E}^{\circ}_{\text{MSS}}[(x_l-1/2)(x_j-1/2)]\right\} \\
&+\frac{1}{N}\left\{u\left(\frac{1}{2}\mathbb{E}^{\circ}_{\text{MSS}}[(x_i-1/2)(1-1/2)]+\frac{1}{2}\mathbb{E}^{\circ}_{\text{MSS}}[(x_i-1/2)(0-1/2)]\right)\right. \\
&\left.+(1-u)\sum_{l\in\mathcal{N}} W^{\circ}_{j\leftarrow l}(\mathbf{x})\mathbb{E}^{\circ}_{\text{MSS}}[(x_i-1/2)(x_l-1/2)]\right\} \\
&+(N-2)\frac{1}{N}\mathbb{E}^{\circ}_{\text{MSS}}[(x_i-1/2)(x_j-1/2)] \\
=&\frac{1}{N}\left\{0+(1-u)\sum_{l\in\mathcal{N}} p_{il}\mathbb{E}^{\circ}_{\text{MSS}}[(x_l-1/2)(x_j-1/2)]\right\} \\
&+\frac{1}{N}\left\{0+(1-u)\sum_{l\in\mathcal{N}} p_{jl}\mathbb{E}^{\circ}_{\text{MSS}}[(x_i-1/2)(x_l-1/2)])\right\} \\
&+(N-2)\frac{1}{N}\mathbb{E}^{\circ}_{\text{MSS}}[(x_i-1/2)(x_j-1/2)].
\end{aligned}
\tag{S42}
$$

Integrating $\mathbb{E}^{\circ}_{\text{MSS}}[(x_i-1/2)(x_j-1/2)]$ into the left-hand side and denoting $\underline{x}_i = x_i - 1/2$, we have

$$
\mathbb{E}^{\circ}_{\text{MSS}}[\underline{x}_i\underline{x}_j] = \frac{1-u}{2}\left(\sum_{l\in\mathcal{N}} p_{il}\mathbb{E}^{\circ}_{\text{MSS}}[\underline{x}_l\underline{x}_j] + \sum_{l\in\mathcal{N}} p_{jl}\mathbb{E}^{\circ}_{\text{MSS}}[\underline{x}_i\underline{x}_l]\right),
\tag{S43}
$$

which is completely the same as the one under the PC rule (see Eq. (S20) for the result). Therefore, the subsequent steps are also the same and are not repeated here. We can ultimately use the defined variables τ_{ij},

$$
\tau_{ij} = \frac{\frac{1}{2} - \mathbb{E}^{\circ}_{\text{RMC}}[x_i x_j]}{K/4},
\tag{S44}
$$

to replace all $\mathbb{E}^{\circ}_{\text{RMC}}[x_i x_j]$.

Substituting all $\mathbb{E}^{\circ}_{\text{RMC}}[x_i x_j]$ into $\mathbb{E}^{\circ}_{\text{RMC}}[(x_i - x_j)(f_i(\mathbf{x}) - f_j(\mathbf{x}))]$ (presented in Eq. (S18)) with computable τ_{ij} using Eq. (S44), and then substituting the result into Eq. (S17), where the positive factors $K/4$ and $1/(2N^2\langle k\rangle)$ can be canceled out, we can organize and obtain the following condition for the success of cooperation:

$$
\mathbb{E}^{\circ}_{\text{RMC}}[\hat{\Delta}'_{\text{sel}}(\mathbf{x})] > 0
$$

$$\Leftrightarrow \sum_{i,j\in\mathcal{N}} k_i p_{ij}^{(2)} \Bigg\{ \left(\frac{rc}{(k_i+1)^2}-c\right)\tau_{ij} + \frac{rc}{k_i+1}\sum_{l\in\mathcal{N}_i}\left(\frac{1}{k_i+1}+\frac{1}{k_l+1}\right)(-\tau_{il}+\tau_{jl})$$
$$+\frac{rc}{k_i+1}\sum_{l\in\mathcal{N}_i}\frac{1}{k_l+1}\sum_{\ell\in\mathcal{N}_l}(-\tau_{i\ell}+\tau_{j\ell}) - \left(\frac{rc}{(k_j+1)^2}-c\right)(-\tau_{ij})$$
$$-\frac{rc}{k_j+1}\sum_{l\in\mathcal{N}_j}\left(\frac{1}{k_j+1}+\frac{1}{k_l+1}\right)(-\tau_{il}+\tau_{jl}) - \frac{rc}{k_j+1}\sum_{l\in\mathcal{N}_j}\frac{1}{k_l+1}\sum_{\ell\in\mathcal{N}_l}(-\tau_{i\ell}+\tau_{j\ell})\Bigg\} > 0$$
$$\Leftrightarrow r > \frac{2\sum_{i,j\in\mathcal{N}} k_i p_{ij}^{(2)}\tau_{ij}}{\sum_{i,j\in\mathcal{N}} k_i p_{ij}^{(2)}(\Upsilon_{ij}+\Upsilon_{ji})}, \tag{S45}$$

where Υ_{ij} are also defined by Eq. (S32) (equivalent to Eq. (5) in the main text) and τ_{ij} can also be obtained by solving the system of Eqs. (S33) (or Eq. (4) in the main text).

According to Eq. (S82), we have $k_i p_{ij}^{(2)} = k_j p_{ji}^{(2)}$. Therefore, we can infer that $\sum_{i,j\in\mathcal{N}} k_i p_{ij}^{(2)}\Upsilon_{ji} = \sum_{i,j\in\mathcal{N}} k_j p_{ji}^{(2)}\Upsilon_{ji} = \sum_{j,i\in\mathcal{N}} k_i p_{ij}^{(2)}\Upsilon_{ij}$, such that $\sum_{i,j\in\mathcal{N}} k_i p_{ij}^{(2)}(\Upsilon_{ij}+\Upsilon_{ji}) = 2\sum_{i,j\in\mathcal{N}} k_i p_{ij}^{(2)}\Upsilon_{ij}$. Therefore, Eq. (S45) can be further simplified as

$$r > \frac{\sum_{i,j\in\mathcal{N}} k_i p_{ij}^{(2)}\tau_{ij}}{\sum_{i,j\in\mathcal{N}} k_i p_{ij}^{(2)}\Upsilon_{ij}}, \tag{S46}$$

which gives the $r^\star$ value under the DB rule.

1.5 Beath-dirth (BD)

For the BD rule, the probability $e_{ij}(\mathbf{x})$ that agent i transmits its strategy to agent j can be calculated as follows. In each elementary MCS, agent i is selected as the focal agent with a probability $W_i(\mathbf{x})$ proportional to its fitness in the population,

$$W_i(\mathbf{x}) = \frac{F_i(\mathbf{x})}{\sum_{l\in\mathcal{N}} F_l(\mathbf{x})}, \tag{S47}$$

and transmits its strategy x_i to a random neighbor. That is,

$$e_{ij}(\mathbf{x}) = W_i(\mathbf{x}) \times p_{ij} = \frac{F_i(\mathbf{x})}{\sum_{l\in\mathcal{N}} F_l(\mathbf{x})} \times p_{ij}. \tag{S48}$$

Taking $\delta = 0$ in Eq. (S48), we have

$$e_{ij}^\circ(\mathbf{x}) = \frac{p_{ij}}{N}. \tag{S49}$$

Taking the derivative of Eq. (S48) with respect to δ at $\delta = 0$, we have

$$e_{ij}'(\mathbf{x}) = \frac{p_{ij}}{N}\left(f_i(\mathbf{x}) - \frac{1}{N}\sum_{l\in\mathcal{N}} f_l(\mathbf{x})\right). \tag{S50}$$

Substituting Eq. (S49) into Eqs. (S5), we obtain

$$\sum_{j\in\mathcal{N}} e_{ji}^\circ(\mathbf{x}) v_i = \sum_{j\in\mathcal{N}} e_{ij}^\circ(\mathbf{x}) v_j \Leftrightarrow \sum_{j\in\mathcal{N}} \frac{p_{ji}}{N} v_i = \sum_{j\in\mathcal{N}} \frac{p_{ij}}{N} v_j, \tag{S51a}$$
$$\sum_{i\in\mathcal{N}} v_i = N. \tag{S51b}$$

The solution to Eqs. (S51) is $v_i = k_i^{-1}/\langle k^{-1}\rangle$ for $i \in \mathcal{N}$, where $k_i^{-1} = 1/k_i$, and $\langle k^{-1}\rangle = (\sum_{i\in\mathcal{N}} k_i^{-1})/N$ represents the average of the reciprocals of the degree of all nodes.

Substituting $v_i = k_i^{-1}/\langle k^{-1}\rangle$ and Eq. (S50) into Eq. (S6), we can calculate $\hat{\Delta}'_{\text{sel}}(\mathbf{x})$ under the BD rule:

$$\hat{\Delta}'_{\text{sel}}(\mathbf{x}) = \frac{1}{2N}\sum_{i,j\in\mathcal{N}} (x_i - x_j)(e_{ij}'(\mathbf{x})v_j - e_{ji}'(\mathbf{x})v_i)$$

$$
\begin{aligned}
&= \frac{1}{2N} \sum_{i,j\in\mathcal{N}} (x_i - x_j) \left(\frac{p_{ij}}{N} \left(f_i(\mathbf{x}) - \frac{1}{N} \sum_{l\in\mathcal{N}} f_l(\mathbf{x}) \right) \frac{k_j^{-1}}{\langle k^{-1}\rangle} - \frac{p_{ji}}{N} \left(f_j(\mathbf{x}) - \frac{1}{N} \sum_{l\in\mathcal{N}} f_l(\mathbf{x}) \right) \frac{k_i^{-1}}{\langle k^{-1}\rangle} \right) \\
&= \frac{1}{2N} \sum_{i,j\in\mathcal{N}} (x_i - x_j) \left(\frac{k_{ij}}{N} \left(f_i(\mathbf{x}) - \frac{1}{N} \sum_{l\in\mathcal{N}} f_l(\mathbf{x}) \right) \frac{k_i^{-1}k_j^{-1}}{\langle k^{-1}\rangle} - \frac{k_{ji}}{N} \left(f_j(\mathbf{x}) - \frac{1}{N} \sum_{l\in\mathcal{N}} f_l(\mathbf{x}) \right) \frac{k_i^{-1}k_j^{-1}}{\langle k^{-1}\rangle} \right) \\
&= \frac{1}{2N^2\langle k^{-1}\rangle} \sum_{i,j\in\mathcal{N}} (x_i - x_j) \frac{k_{ij}}{k_i k_j} (f_i(\mathbf{x}) - f_j(\mathbf{x})).
\end{aligned}
\tag{S52}
$$

Substituting Eq. (S52) into Eq. (S2), we obtain the condition for the success of cooperation under the BD rule:

$$
\mathbb{E}^{\circ}_{\mathrm{RMC}}[\hat{\Delta}'_{\mathrm{sel}}(\mathbf{x})] > 0 \Leftrightarrow \frac{1}{2N^2\langle k^{-1}\rangle} \sum_{i,j\in\mathcal{N}} \frac{k_{ij}}{k_i k_j} \mathbb{E}^{\circ}_{\mathrm{RMC}}[(x_i - x_j)(f_i(\mathbf{x}) - f_j(\mathbf{x}))] > 0. \tag{S53}
$$

We first calculate $\mathbb{E}^{\circ}_{\mathrm{RMC}}[(x_i - x_j)(f_i(\mathbf{x}) - f_j(\mathbf{x}))]$ in Eq. (S53), which is the same as the one calculated under the PC rule (see Eq. (S18) for the same result). The remaining work is to calculate $\mathbb{E}^{\circ}_{\mathrm{RMC}}[x_i x_j]$ for all $i, j \in \mathcal{N}$ under the BD rule.

Similarly, we begin with the MSS distribution and derive the recurrence relation by working through an elementary MCS. For the mutation mechanism under the BD rule, we specify that the event of a mutation is assigned to the focal agent rather than being transmitted to a random neighbor by the BD rule. This ensures the probability of the initial mutation on each node is $1/N$ in a fixation state, consistent with the PC and DB rules.

Therefore, under the BD rule, the possible events that happen within an elementary MCS can be classified into the following categories.

- Agent i is selected as the focal agent with probability $W_i^{\circ}(\mathbf{x}) = 1/N$ to propagate its strategy:

  (i) The focal agent i mutates with probability u, becoming cooperation ($x_i \leftarrow 1$) with probability $1/2$, or defection ($x_i \leftarrow 0$) with probability $1/2$;

  (ii) Agent i transmits the strategy to a random neighbor with probability $1-u$. With probability $p_{i\ell}$, agent i transmits its strategy to agent ℓ ($\ell \in \mathcal{N}$), $x_\ell \leftarrow x_i$. If $\ell = j$, this influences the quantity $\mathbb{E}^{\circ}_{\mathrm{MSS}}[(x_i - 1/2)(x_j - 1/2)]$; otherwise the quantity keeps unchanged.

- Similarly, agent j is selected as the focal agent with probability $W_j^{\circ}(\mathbf{x}) = 1/N$ to propagate its strategy:

  (i) The focal agent j mutates with probability u, becoming cooperation ($x_j \leftarrow 1$) with probability $1/2$, or defection ($x_j \leftarrow 0$) with probability $1/2$;

  (ii) Agent j transmits the strategy to a random neighbor with probability $1-u$. With probability $p_{j\ell}$, agent j transmits its strategy to agent ℓ ($\ell \in \mathcal{N}$), $x_\ell \leftarrow x_j$. Similarly, if $\ell = i$, this influences the quantity $\mathbb{E}^{\circ}_{\mathrm{MSS}}[(x_i - 1/2)(x_j - 1/2)]$; otherwise the quantity keeps unchanged.

- The focal agent, denoted by $l \in \mathcal{N} \setminus \{i, j\}$, is one of the remaining $N-2$ agents other than i and j, with probability $W_l^{\circ}(\mathbf{x}) = 1/N$:

  (i) The focal agent l mutates with probability u, which can only change x_l and has nothing to do with x_i or x_j;

  (ii) Agent l transmits the strategy to a random neighbor with probability $1-u$. With probability $p_{l\ell}$, agent l transmits its strategy to agent ℓ ($\ell \in \mathcal{N}$), $x_\ell \leftarrow x_l$. If $\ell = i$ or $\ell = j$, this influences the quantity $\mathbb{E}^{\circ}_{\mathrm{MSS}}[(x_i - 1/2)(x_j - 1/2)]$; otherwise the quantity keeps unchanged.

Combining all the above possibilities of an elementary MCS, we obtain the following recurrence relation under the MSS distribution:

$$
\begin{aligned}
&\mathbb{E}^{\circ}_{\mathrm{MSS}}[(x_i - 1/2)(x_j - 1/2)] \\
&= W_i^{\circ}(\mathbf{x}) \Bigg\{ u \left(\frac{1}{2} \mathbb{E}^{\circ}_{\mathrm{MSS}}[(1 - 1/2)(x_j - 1/2)] + \frac{1}{2} \mathbb{E}^{\circ}_{\mathrm{MSS}}[(0 - 1/2)(x_j - 1/2)] \right) \\
&\quad + (1-u) \left(\sum_{\ell\in\mathcal{N}\setminus\{j\}} p_{i\ell} \mathbb{E}^{\circ}_{\mathrm{MSS}}[(x_i - 1/2)(x_j - 1/2)] + p_{ij} \mathbb{E}^{\circ}_{\mathrm{MSS}}[(x_i - 1/2)(x_i - 1/2)] \right) \Bigg\} \\
&\quad + W_j^{\circ}(\mathbf{x}) \Bigg\{ u \left(\frac{1}{2} \mathbb{E}^{\circ}_{\mathrm{MSS}}[(x_i - 1/2)(1 - 1/2)] + \frac{1}{2} \mathbb{E}^{\circ}_{\mathrm{MSS}}[(x_i - 1/2)(0 - 1/2)] \right)
\end{aligned}
$$

$$
\begin{aligned}
&+(1-u)\left(\sum_{\ell\in\mathcal{N}\setminus\{i\}} p_{j\ell}\mathbb{E}^{\circ}_{\mathrm{MSS}}[(x_i-1/2)(x_j-1/2)]+p_{ji}\mathbb{E}^{\circ}_{\mathrm{MSS}}[(x_j-1/2)(x_j-1/2)]\right)\Bigg\}\\
&+\sum_{l\in\mathcal{N}\setminus\{i,j\}} W^{\circ}_l(\mathbf{x})\Bigg\{u\mathbb{E}^{\circ}_{\mathrm{MSS}}[(x_i-1/2)(x_j-1/2)]+(1-u)\left(\sum_{\ell\in\mathcal{N}\setminus\{i,j\}} p_{l\ell}\mathbb{E}^{\circ}_{\mathrm{MSS}}[(x_i-1/2)(x_j-1/2)]\right.\\
&\left.+p_{li}\mathbb{E}^{\circ}_{\mathrm{MSS}}[(x_l-1/2)(x_j-1/2)]+p_{lj}\mathbb{E}^{\circ}_{\mathrm{MSS}}[(x_i-1/2)(x_l-1/2)]\right)\Bigg\}\\
=&\frac{1}{N}\Bigg\{0+(1-u)\left(\sum_{\ell\in\mathcal{N}\setminus\{j\}} p_{i\ell}\mathbb{E}^{\circ}_{\mathrm{MSS}}[(x_i-1/2)(x_j-1/2)]+p_{ij}\mathbb{E}^{\circ}_{\mathrm{MSS}}[(x_i-1/2)(x_i-1/2)]\right)\Bigg\}\\
&+\frac{1}{N}\Bigg\{0+(1-u)\left(\sum_{\ell\in\mathcal{N}\setminus\{i\}} p_{j\ell}\mathbb{E}^{\circ}_{\mathrm{MSS}}[(x_i-1/2)(x_j-1/2)]+p_{ji}\mathbb{E}^{\circ}_{\mathrm{MSS}}[(x_j-1/2)(x_j-1/2)]\right)\Bigg\}\\
&+\frac{1}{N}\sum_{l\in\mathcal{N}\setminus\{i,j\}}\Bigg\{u\mathbb{E}^{\circ}_{\mathrm{MSS}}[(x_i-1/2)(x_j-1/2)]+(1-u)\left(\sum_{\ell\in\mathcal{N}\setminus\{i,j\}} p_{l\ell}\mathbb{E}^{\circ}_{\mathrm{MSS}}[(x_i-1/2)(x_j-1/2)]\right.\\
&\left.+p_{li}\mathbb{E}^{\circ}_{\mathrm{MSS}}[(x_l-1/2)(x_j-1/2)]+p_{lj}\mathbb{E}^{\circ}_{\mathrm{MSS}}[(x_i-1/2)(x_l-1/2)]\right)\Bigg\}\\
=&\frac{1-u}{N}\sum_{l\in\mathcal{N}}\left(p_{li}\mathbb{E}^{\circ}_{\mathrm{MSS}}[(x_l-1/2)(x_j-1/2)]+p_{lj}\mathbb{E}^{\circ}_{\mathrm{MSS}}[(x_i-1/2)(x_l-1/2)]\right)\\
&+\frac{u}{N}\sum_{l\in\mathcal{N}\setminus\{i,j\}}\mathbb{E}^{\circ}_{\mathrm{MSS}}[(x_i-1/2)(x_j-1/2)]+\frac{1-u}{N}\sum_{l\in\mathcal{N}}\sum_{\ell\in\mathcal{N}\setminus\{i,j\}} p_{l\ell}\mathbb{E}^{\circ}_{\mathrm{MSS}}[(x_i-1/2)(x_j-1/2)]\\
=&\frac{1-u}{N}\sum_{l\in\mathcal{N}}\left(p_{li}\mathbb{E}^{\circ}_{\mathrm{MSS}}[(x_l-1/2)(x_j-1/2)]+p_{lj}\mathbb{E}^{\circ}_{\mathrm{MSS}}[(x_i-1/2)(x_l-1/2)]\right)\\
&+\frac{u}{N}(N-2)\mathbb{E}^{\circ}_{\mathrm{MSS}}[(x_i-1/2)(x_j-1/2)]+\frac{1-u}{N}\sum_{l\in\mathcal{N}}(1-p_{li}-p_{lj})\mathbb{E}^{\circ}_{\mathrm{MSS}}[(x_i-1/2)(x_j-1/2)]\\
=&\frac{1-u}{N}\sum_{l\in\mathcal{N}}\left(p_{li}\mathbb{E}^{\circ}_{\mathrm{MSS}}[(x_l-1/2)(x_j-1/2)]+p_{lj}\mathbb{E}^{\circ}_{\mathrm{MSS}}[(x_i-1/2)(x_l-1/2)]\right)\\
&+\left(1-\frac{2u}{N}-\frac{1-u}{N}\sum_{l\in\mathcal{N}}(p_{li}+p_{lj})\right)\mathbb{E}^{\circ}_{\mathrm{MSS}}[(x_i-1/2)(x_j-1/2)].
\end{aligned}
\tag{S54}
$$

Integrating $\mathbb{E}^{\circ}_{\mathrm{MSS}}[(x_i-1/2)(x_j-1/2)]$ into the left-hand side and denoting $\underline{x}_i=x_i-1/2$, we obtain

$$
\begin{aligned}
&\mathbb{E}^{\circ}_{\mathrm{MSS}}[\underline{x}_i\underline{x}_j]=\frac{1-u}{2u+(1-u)\sum_{l\in\mathcal{N}}(p_{li}+p_{lj})}\left(\sum_{l\in\mathcal{N}} p_{li}\mathbb{E}^{\circ}_{\mathrm{MSS}}[\underline{x}_l\underline{x}_j]+\sum_{l\in\mathcal{N}} p_{lj}\mathbb{E}^{\circ}_{\mathrm{MSS}}[\underline{x}_i\underline{x}_l]\right)\\
\Leftrightarrow&\left(\frac{2u}{(1-u)\sum_{l\in\mathcal{N}}(p_{li}+p_{lj})}+1\right)\mathbb{E}^{\circ}_{\mathrm{MSS}}[\underline{x}_i\underline{x}_j]=\frac{1}{\sum_{l\in\mathcal{N}}(p_{li}+p_{lj})}\left(\sum_{l\in\mathcal{N}} p_{li}\mathbb{E}^{\circ}_{\mathrm{MSS}}[\underline{x}_l\underline{x}_j]+\sum_{l\in\mathcal{N}} p_{lj}\mathbb{E}^{\circ}_{\mathrm{MSS}}[\underline{x}_i\underline{x}_l]\right).
\end{aligned}
\tag{S55}
$$

We define variables $\tilde{\phi}_{ij}(\mathbf{x})$,

$$
\tilde{\phi}_{ij}(\mathbf{x})=\underline{x}_i\underline{x}_j-\frac{1}{\sum_{l\in\mathcal{N}}(p_{li}+p_{lj})}\left(\sum_{l\in\mathcal{N}} p_{li}\underline{x}_l\underline{x}_j+\sum_{l\in\mathcal{N}} p_{lj}\underline{x}_i\underline{x}_l\right),
\tag{S56}
$$

which satisfy the properties: $\tilde{\phi}_{ij}(\mathbf{C})=\tilde{\phi}_{ij}(\mathbf{D})=0$. Therefore, $\tilde{\phi}_{ij}(\mathbf{x})$ can be used to relate the MSS and RMC distributions through Eq. (S11) as $u\to 0$.

We first calculate $\mathbb{E}^{\circ}_{\mathrm{MSS}}[\tilde{\phi}_{ij}(\mathbf{x})]$. Writing down the expected value of Eq. (S56) and using Eq. (S55), we have

$$
\mathbb{E}^{\circ}_{\mathrm{MSS}}[\tilde{\phi}_{ij}(\mathbf{x})]=\mathbb{E}^{\circ}_{\mathrm{MSS}}[\underline{x}_i\underline{x}_j]-\frac{1}{\sum_{l\in\mathcal{N}}(p_{li}+p_{lj})}\left(\sum_{l\in\mathcal{N}} p_{li}\mathbb{E}^{\circ}_{\mathrm{MSS}}[\underline{x}_l\underline{x}_j]+\sum_{l\in\mathcal{N}} p_{lj}\mathbb{E}^{\circ}_{\mathrm{MSS}}[\underline{x}_i\underline{x}_l]\right)
$$

$$
\begin{aligned}
&= \mathbb{E}^{\circ}_{\mathrm{MSS}}[\underline{x}_i\underline{x}_j] - \left(\frac{2u}{(1-u)\sum_{l\in\mathcal{N}}(p_{li}+p_{lj})}+1\right)\mathbb{E}^{\circ}_{\mathrm{MSS}}[\underline{x}_i\underline{x}_j] \\
&= -\frac{2u}{(1-u)\sum_{l\in\mathcal{N}}(p_{li}+p_{lj})}\mathbb{E}^{\circ}_{\mathrm{MSS}}[\underline{x}_i\underline{x}_j]. \qquad \text{(S57)}
\end{aligned}
$$

According to Eq. (S11), we can calculate $\mathbb{E}^{\circ}_{\mathrm{RMC}}[\tilde{\phi}_{ij}(\mathbf{x})]$ from $\mathbb{E}^{\circ}_{\mathrm{MSS}}[\tilde{\phi}_{ij}(\mathbf{x})]$,

$$
\begin{aligned}
\mathbb{E}^{\circ}_{\mathrm{RMC}}[\tilde{\phi}_{ij}(\mathbf{x})] &= K\left.\frac{\mathrm{d}\mathbb{E}^{\circ}_{\mathrm{MSS}}[\tilde{\phi}_{ij}(\mathbf{x})]}{\mathrm{d}u}\right|_{u=0} \\
&= K\left.\frac{\mathrm{d}}{\mathrm{d}u}\right|_{u=0}\left(-\frac{2u}{(1-u)\sum_{l\in\mathcal{N}}(p_{li}+p_{lj})}\mathbb{E}^{\circ}_{\mathrm{MSS}}[\underline{x}_i\underline{x}_j]\right) \\
&= K\left(-\frac{2}{\sum_{l\in\mathcal{N}}(p_{li}+p_{lj})}\left.\mathbb{E}^{\circ}_{\mathrm{MSS}}[\underline{x}_i\underline{x}_j]\right|_{u=0}+0\right) \\
&= -\frac{K}{2\sum_{l\in\mathcal{N}}(p_{li}+p_{lj})}. \qquad \text{(S58)}
\end{aligned}
$$

On the other hand, writing down the expected value of Eq. (S56) under the RMC distribution leads to another expression of $\mathbb{E}^{\circ}_{\mathrm{RMC}}[\tilde{\phi}_{ij}(\mathbf{x})]$:

$$
\mathbb{E}^{\circ}_{\mathrm{RMC}}[\tilde{\phi}_{ij}(\mathbf{x})] = \mathbb{E}^{\circ}_{\mathrm{RMC}}[\underline{x}_i\underline{x}_j] - \frac{1}{\sum_{l\in\mathcal{N}}(p_{li}+p_{lj})}\left(\sum_{l\in\mathcal{N}} p_{li}\mathbb{E}^{\circ}_{\mathrm{RMC}}[\underline{x}_l\underline{x}_j] + \sum_{l\in\mathcal{N}} p_{lj}\mathbb{E}^{\circ}_{\mathrm{RMC}}[\underline{x}_i\underline{x}_l]\right). \qquad \text{(S59)}
$$

Substituting the result of Eq. (S58) into Eq. (S59), we have

$$
\mathbb{E}^{\circ}_{\mathrm{RMC}}[\underline{x}_i\underline{x}_j] = \frac{1}{\sum_{l\in\mathcal{N}}(p_{li}+p_{lj})}\left(\sum_{l\in\mathcal{N}} p_{li}\mathbb{E}^{\circ}_{\mathrm{RMC}}[\underline{x}_l\underline{x}_j] + \sum_{l\in\mathcal{N}} p_{lj}\mathbb{E}^{\circ}_{\mathrm{RMC}}[\underline{x}_i\underline{x}_l]\right) - \frac{K}{2\sum_{l\in\mathcal{N}}(p_{li}+p_{lj})}. \qquad \text{(S60)}
$$

We define variables $\tilde{\tau}_{ij}$ for $i, j \in \mathcal{N}$,

$$
\tilde{\tau}_{ij} = \frac{\frac{1}{2} - \mathbb{E}^{\circ}_{\mathrm{RMC}}[x_i x_j]}{K/2}. \qquad \text{(S61)}
$$

Obviously, $\tilde{\tau}_{ii} = 0$ when $i = j$, because $\mathbb{E}^{\circ}_{\mathrm{RMC}}[x_i^2] = 1/2$. Also, $\tilde{\tau}_{ij} = \tilde{\tau}_{ji}$, because $\mathbb{E}^{\circ}_{\mathrm{RMC}}[x_i x_j] = \mathbb{E}^{\circ}_{\mathrm{RMC}}[x_j x_i]$.

When $i \neq j$, we can solve for the values of $\tilde{\tau}_{ij}$ by the recurrence relation. According to Eq. (S27), we know that Eq. (S61) can be written as

$$
\tilde{\tau}_{ij} = \frac{\frac{1}{2} - \left(\mathbb{E}^{\circ}_{\mathrm{RMC}}[\underline{x}_i\underline{x}_j] + \frac{1}{4}\right)}{K/2} = \frac{\frac{1}{2} - 2\mathbb{E}^{\circ}_{\mathrm{RMC}}[\underline{x}_i\underline{x}_j]}{K} \qquad \text{(S62)}
$$

or

$$
\mathbb{E}^{\circ}_{\mathrm{RMC}}[\underline{x}_i\underline{x}_j] = \frac{\frac{1}{2} - K\tilde{\tau}_{ij}}{2}. \qquad \text{(S63)}
$$

Substituting Eq. (S63) into Eq. (S60), we obtain the recurrence relation of $\tilde{\tau}_{ij}$:

$$
\begin{aligned}
&\frac{\frac{1}{2} - K\tilde{\tau}_{ij}}{2} = \frac{1}{\sum_{l\in\mathcal{N}}(p_{li}+p_{lj})}\left(\sum_{l\in\mathcal{N}} p_{li}\frac{\frac{1}{2} - K\tilde{\tau}_{jl}}{2} + \sum_{l\in\mathcal{N}} p_{lj}\frac{\frac{1}{2} - K\tilde{\tau}_{il}}{2}\right) - \frac{K}{2\sum_{l\in\mathcal{N}}(p_{li}+p_{lj})} \\
&\Leftrightarrow \tilde{\tau}_{ij} = \frac{1}{\sum_{l\in\mathcal{N}}(p_{li}+p_{lj})}\left(1 + \sum_{l\in\mathcal{N}} p_{li}\tilde{\tau}_{jl} + \sum_{l\in\mathcal{N}} p_{lj}\tilde{\tau}_{il}\right). \qquad \text{(S64)}
\end{aligned}
$$

The recurrence relation Eq. (S64), together with $\tilde{\tau}_{ii} = 0$, form a system of linear equations, through which all $\tilde{\tau}_{ij}$ values $(i, j \in \mathcal{N})$ can be determined on a given network.

Substituting all $\mathbb{E}^{\circ}_{\mathrm{RMC}}[x_i x_j]$ into Eq. (S18) with computable $\tilde{\tau}_{ij}$ using Eq. (S61), and then substituting the result into the cooperation success condition Eq. (S53), where the positive factors $K/2$ and $1/(2N^2\langle k^{-1}\rangle)$ can be canceled out, we arrive at the following condition for the success of cooperation:

$$
\begin{aligned}
&\mathbb{E}^{\circ}_{\mathrm{RMC}}[\hat{\Delta}'_{\mathrm{sel}}(\mathbf{x})] > 0\\
\Leftrightarrow &\sum_{i,j\in\mathcal{N}} \frac{k_{ij}}{k_i k_j}\Bigg\{ \left(\frac{rc}{(k_i+1)^2}-c\right)\tilde{\tau}_{ij} + \frac{rc}{k_i+1}\sum_{l\in\mathcal{N}_i}\left(\frac{1}{k_i+1}+\frac{1}{k_l+1}\right)(-\tilde{\tau}_{il}+\tilde{\tau}_{jl})\\
&+\frac{rc}{k_i+1}\sum_{l\in\mathcal{N}_i}\frac{1}{k_l+1}\sum_{\ell\in\mathcal{N}_l}(-\tilde{\tau}_{i\ell}+\tilde{\tau}_{j\ell}) - \left(\frac{rc}{(k_j+1)^2}-c\right)(-\tilde{\tau}_{ij})\\
&-\frac{rc}{k_j+1}\sum_{l\in\mathcal{N}_j}\left(\frac{1}{k_j+1}+\frac{1}{k_l+1}\right)(-\tilde{\tau}_{il}+\tilde{\tau}_{jl}) - \frac{rc}{k_j+1}\sum_{l\in\mathcal{N}_j}\frac{1}{k_l+1}\sum_{\ell\in\mathcal{N}_l}(-\tilde{\tau}_{i\ell}+\tilde{\tau}_{j\ell})\Bigg\} > 0\\
\Leftrightarrow\ & r > \frac{2\sum_{i,j\in\mathcal{N}}\dfrac{k_{ij}}{k_i k_j}\tilde{\tau}_{ij}}{\sum_{i,j\in\mathcal{N}}\dfrac{k_{ij}}{k_i k_j}(\tilde{\Upsilon}_{ij}+\tilde{\Upsilon}_{ji})},
\end{aligned}
\tag{S65}
$$

where $\tilde{\Upsilon}_{ij}$ are defined by

$$
\tilde{\Upsilon}_{ij} = \frac{1}{k_i+1}\left(\frac{\tilde{\tau}_{ij}+k_i\sum_{l\in\mathcal{N}}p_{il}(\tilde{\tau}_{jl}-\tilde{\tau}_{il})}{k_i+1} + k_i\sum_{l\in\mathcal{N}}p_{il}\frac{(\tilde{\tau}_{jl}-\tilde{\tau}_{il})+k_l\sum_{\ell\in\mathcal{N}}p_{l\ell}(\tilde{\tau}_{j\ell}-\tilde{\tau}_{i\ell})}{k_l+1}\right). \tag{S66}
$$

And according to the previous discussion, $\tilde{\tau}_{ij}$ can be solved by the following system of linear equations:

$$
\begin{cases}
\tilde{\tau}_{ij} = \dfrac{1}{\sum_{l\in\mathcal{N}}(p_{li}+p_{lj})}\left(1+\sum_{l\in\mathcal{N}}(p_{li}\tilde{\tau}_{jl}+p_{lj}\tilde{\tau}_{il})\right), & \text{if } j\neq i,\\
\tilde{\tau}_{ij}=0, & \text{if } j=i.
\end{cases}
\tag{S67}
$$

In applications, we can use the following equivalent form, which is more intuitive for calculation:

$$
\begin{cases}
\tilde{\tau}_{ij} = \dfrac{1}{\sum_{l\in\mathcal{N}_i}k_l^{-1}+\sum_{l\in\mathcal{N}_j}k_l^{-1}}\left(1+\sum_{l\in\mathcal{N}_i}k_l^{-1}\tilde{\tau}_{jl}+\sum_{l\in\mathcal{N}_j}k_l^{-1}\tilde{\tau}_{il}\right), & \text{if } j\neq i,\\
\tilde{\tau}_{ij}=0, & \text{if } j=i.
\end{cases}
\tag{S68}
$$

Finally, $\sum_{i,j\in\mathcal{N}}k_{ij}/(k_ik_j)\tilde{\Upsilon}_{ji} = \sum_{i,j\in\mathcal{N}}k_{ij}/(k_ik_j)\tilde{\Upsilon}_{ij}$. Therefore, $\sum_{i,j\in\mathcal{N}}k_{ij}/(k_ik_j)(\tilde{\Upsilon}_{ij}+\tilde{\Upsilon}_{ji}) = 2\sum_{i,j\in\mathcal{N}}k_{ij}/(k_ik_j)\tilde{\Upsilon}_{ij}$. As a result, Eq. (S65) can be further simplified as

$$
r > \frac{\sum_{i,j\in\mathcal{N}}\dfrac{k_{ij}}{k_ik_j}\tilde{\tau}_{ij}}{\sum_{i,j\in\mathcal{N}}\dfrac{k_{ij}}{k_ik_j}\tilde{\Upsilon}_{ij}}. \tag{S69}
$$

The right-hand side is the $r^\star$ value under the BD rule. Furthermore, let $\tilde{\tau}^{(1)} = \sum_{i,j\in\mathcal{N}}k_{ij}/(k_ik_j)\tilde{\tau}_{ij}$, $\tilde{\Upsilon}^{(1)} = \sum_{i,j\in\mathcal{N}}k_{ij}/(k_ik_j)\tilde{\Upsilon}_{ij}$, then the $r^\star$ value under the BD rule can also be expressed in shorthand as

$$
r^\star = \frac{\tilde{\tau}^{(1)}}{\tilde{\Upsilon}^{(1)}}. \tag{S70}
$$

1.6 Variation of the model: accumulated payoff

In the previous deduction, we assume that an agent i's actual payoff is averaged over the $1+k_i$ games organized by itself and its neighbors, ensuring the consistency of payoff scales across different numbers of neighbors. Another approach is to take the accumulated payoff from these games. On homogeneous graphs, there is no difference between these two approaches in the weak selection limit, but on heterogeneous graphs, agents with more neighbors tend to have higher or lower payoffs because they participate in more games. From a physical perspective, the accumulated payoff is also a more intuitive model detail in real-world systems. We are thus interested in examining the conditions for the success of cooperation when using accumulated payoffs.

1.6.1 Modified payoff calculation

We do not normalize agent i's actual payoff by dividing $1+k_i$ but take the accumulated payoff directly. Similar to Eq. (S1), the calculation of agent i's actual payoff $f_i(\mathbf{x})$ follows Eq. (S71).

$$\begin{aligned} f_i(\mathbf{x}) &= \sum_{l\in\mathscr{G}_i}\left(\frac{r\sum_{\ell\in\mathscr{G}_l}x_\ell c}{G_l}-x_ic\right)\\ &= \left(\frac{r(x_i+\sum_{l\in\mathscr{N}_i}x_l)c}{k_i+1}-x_ic\right)+\sum_{l\in\mathscr{N}_i}\left(\frac{r(x_l+\sum_{\ell\in\mathscr{N}_l}x_\ell)c}{k_l+1}-x_ic\right)\\ &= \left(\frac{rc}{k_i+1}-(k_i+1)c\right)x_i+rc\sum_{l\in\mathscr{N}_i}\left(\frac{1}{k_i+1}+\frac{1}{k_l+1}\right)x_l+rc\sum_{l\in\mathscr{N}_i}\frac{1}{k_l+1}\sum_{\ell\in\mathscr{N}_l}x_\ell. \end{aligned} \tag{S71}$$

The dynamics of strategy evolution remain the same under neutral drift. Only the quantity $\mathbb{E}^{\circ}_{\mathrm{RMC}}[(x_i-x_j)(f_i(\mathbf{x})-f_j(\mathbf{x}))]$ is influenced by the modified payoff calculation $f_i(\mathbf{x})$ ($f_j(\mathbf{x})$) and is recalculated as follows.

$$\begin{aligned} &\mathbb{E}^{\circ}_{\mathrm{RMC}}[(x_i-x_j)(f_i(\mathbf{x})-f_j(\mathbf{x}))]\\ =&\,\mathbb{E}^{\circ}_{\mathrm{RMC}}\Bigg[\left(\frac{rc}{k_i+1}-(k_i+1)c\right)(x_i^2-x_ix_j)+rc\sum_{l\in\mathscr{N}_i}\left(\frac{1}{k_i+1}+\frac{1}{k_l+1}\right)(x_ix_l-x_jx_l)\\ &+rc\sum_{l\in\mathscr{N}_i}\frac{1}{k_l+1}\sum_{\ell\in\mathscr{N}_l}(x_ix_\ell-x_jx_\ell)-\left(\frac{rc}{k_j+1}-(k_j+1)c\right)(x_ix_j-x_j^2)\\ &-rc\sum_{l\in\mathscr{N}_j}\left(\frac{1}{k_j+1}+\frac{1}{k_l+1}\right)(x_ix_l-x_jx_l)-rc\sum_{l\in\mathscr{N}_j}\frac{1}{k_l+1}\sum_{\ell\in\mathscr{N}_l}(x_ix_\ell-x_jx_\ell)\Bigg]\\ =&\left(\frac{rc}{k_i+1}-(k_i+1)c\right)\left(\mathbb{E}^{\circ}_{\mathrm{RMC}}[x_i^2]-\mathbb{E}^{\circ}_{\mathrm{RMC}}[x_ix_j]\right)+rc\sum_{l\in\mathscr{N}_i}\left(\frac{1}{k_i+1}+\frac{1}{k_l+1}\right)\left(\mathbb{E}^{\circ}_{\mathrm{RMC}}[x_ix_l]-\mathbb{E}^{\circ}_{\mathrm{RMC}}[x_jx_l]\right)\\ &+rc\sum_{l\in\mathscr{N}_i}\frac{1}{k_l+1}\sum_{\ell\in\mathscr{N}_l}\left(\mathbb{E}^{\circ}_{\mathrm{RMC}}[x_ix_\ell]-\mathbb{E}^{\circ}_{\mathrm{RMC}}[x_jx_\ell]\right)-\left(\frac{rc}{k_j+1}-(k_j+1)c\right)\left(\mathbb{E}^{\circ}_{\mathrm{RMC}}[x_ix_j]-\mathbb{E}^{\circ}_{\mathrm{RMC}}[x_j^2]\right)\\ &-rc\sum_{l\in\mathscr{N}_j}\left(\frac{1}{k_j+1}+\frac{1}{k_l+1}\right)\left(\mathbb{E}^{\circ}_{\mathrm{RMC}}[x_ix_l]-\mathbb{E}^{\circ}_{\mathrm{RMC}}[x_jx_l]\right)-rc\sum_{l\in\mathscr{N}_j}\frac{1}{k_l+1}\sum_{\ell\in\mathscr{N}_l}\left(\mathbb{E}^{\circ}_{\mathrm{RMC}}[x_ix_\ell]-\mathbb{E}^{\circ}_{\mathrm{RMC}}[x_jx_\ell]\right). \end{aligned} \tag{S72}$$

1.6.2 Pairwise comparison

The cooperation condition under the PC rule is still Eq. (S17), because the condition was obtained under neutral drift and thus remains independent of the later introduced marginal effect of games. Using the result of Eq. (S72) and $\tau_{ij} = (1/2-\mathbb{E}^{\circ}_{\mathrm{RMC}}[x_ix_j])/(K/4)$ as defined by Eq. (S26), we calculate

$$\begin{aligned} &\frac{1}{4N^2\langle k\rangle}\sum_{i,j\in\mathscr{N}}k_ip_{ij}\mathbb{E}^{\circ}_{\mathrm{RMC}}[(x_i-x_j)(f_i(\mathbf{x})-f_j(\mathbf{x}))]>0\\ \Leftrightarrow&\sum_{i,j\in\mathscr{N}}k_ip_{ij}\Bigg\{\left(\frac{rc}{k_i+1}-(k_i+1)c\right)\tau_{ij}+rc\sum_{l\in\mathscr{N}_i}\left(\frac{1}{k_i+1}+\frac{1}{k_l+1}\right)(-\tau_{il}+\tau_{jl})\\ &+rc\sum_{l\in\mathscr{N}_i}\frac{1}{k_l+1}\sum_{\ell\in\mathscr{N}_l}(-\tau_{i\ell}+\tau_{j\ell})-\left(\frac{rc}{k_j+1}-(k_j+1)c\right)(-\tau_{ij})\\ &-rc\sum_{l\in\mathscr{N}_j}\left(\frac{1}{k_j+1}+\frac{1}{k_l+1}\right)(-\tau_{il}+\tau_{jl})-rc\sum_{l\in\mathscr{N}_j}\frac{1}{k_l+1}\sum_{\ell\in\mathscr{N}_l}(-\tau_{i\ell}+\tau_{j\ell})\Bigg\}>0\\ \Leftrightarrow&\, r>\frac{\sum_{i,j\in\mathscr{N}}k_ip_{ij}(k_i+k_j+2)\tau_{ij}}{\sum_{i,j\in\mathscr{N}}k_ip_{ij}[(k_i+1)\Upsilon_{ij}+(k_j+1)\Upsilon_{ji}]}. \end{aligned} \tag{S73}$$

Further simplifying Eq. (S73) (using Eq. (S83)) leads to

$$r>\frac{\sum_{i,j\in\mathscr{N}}k_i(k_i+1)p_{ij}\tau_{ij}}{\sum_{i,j\in\mathscr{N}}k_i(k_i+1)p_{ij}\Upsilon_{ij}}. \tag{S74}$$

The right-hand side is the $r^\star$ value for the success of cooperation when using accumulated payoffs under the PC rule. The τ_{ij} values are still obtained by solving Eqs. (S33) on the given network, and the Υ_{ij} values are still obtained by Eq. (S32).

1.6.3 Death-birth

The cooperation condition under the DB rule is Eq. (S41). Using the result of Eq. (S72) and $\tau_{ij} = (1/2 - \mathbb{E}^{\circ}_{\mathrm{RMC}}[x_i x_j])/(K/4)$ as defined by Eq. (S44), we calculate

$$\begin{aligned}
&\frac{1}{2N^2\langle k\rangle}\sum_{i,j\in\mathcal{N}} k_i p_{ij}^{(2)} \mathbb{E}^{\circ}_{\mathrm{RMC}}[(x_i - x_j)(f_i(\mathbf{x}) - f_j(\mathbf{x}))] > 0 \\
\Leftrightarrow &\sum_{i,j\in\mathcal{N}} k_i p_{ij}^{(2)} \Bigg\{ \left(\frac{rc}{k_i+1} - (k_i+1)c\right)\tau_{ij} + rc\sum_{l\in\mathcal{N}_i}\left(\frac{1}{k_i+1}+\frac{1}{k_l+1}\right)(-\tau_{il}+\tau_{jl}) \\
&+ rc\sum_{l\in\mathcal{N}_i}\frac{1}{k_l+1}\sum_{\ell\in\mathcal{N}_l}(-\tau_{i\ell}+\tau_{j\ell}) - \left(\frac{rc}{k_j+1} - (k_j+1)c\right)(-\tau_{ij}) \\
&- rc\sum_{l\in\mathcal{N}_j}\left(\frac{1}{k_j+1}+\frac{1}{k_l+1}\right)(-\tau_{il}+\tau_{jl}) - rc\sum_{l\in\mathcal{N}_j}\frac{1}{k_l+1}\sum_{\ell\in\mathcal{N}_l}(-\tau_{i\ell}+\tau_{j\ell}) \Bigg\} > 0 \\
\Leftrightarrow &\; r > \frac{\sum_{i,j\in\mathcal{N}} k_i p_{ij}^{(2)} (k_i+k_j+2)\tau_{ij}}{\sum_{i,j\in\mathcal{N}} k_i p_{ij}^{(2)} [(k_i+1)\Upsilon_{ij} + (k_j+1)\Upsilon_{ji}]}.
\end{aligned} \tag{S75}$$

Further simplifying Eq. (S75) leads to

$$r > \frac{\sum_{i,j\in\mathcal{N}} k_i(k_i+1) p_{ij}^{(2)} \tau_{ij}}{\sum_{i,j\in\mathcal{N}} k_i(k_i+1) p_{ij}^{(2)} \Upsilon_{ij}}. \tag{S76}$$

The right-hand side is the $r^\star$ value for the success of cooperation when using accumulated payoffs under the DB rule. The τ_{ij} values are still obtained by solving Eqs. (S33) on the given network, and the Υ_{ij} values are still obtained by Eq. (S32).

1.6.4 Birth-death

The cooperation condition under the BD rule is Eq. (S53). Using the result of Eq. (S72) and $\tau_{ij} = (1/2 - \mathbb{E}^{\circ}_{\mathrm{RMC}}[x_i x_j])/(K/2)$ as defined by Eq. (S61), we calculate

$$\begin{aligned}
&\frac{1}{2N^2\langle k^{-1}\rangle}\sum_{i,j\in\mathcal{N}} \frac{k_{ij}}{k_i k_j} \mathbb{E}^{\circ}_{\mathrm{RMC}}[(x_i - x_j)(f_i(\mathbf{x}) - f_j(\mathbf{x}))] > 0 \\
\Leftrightarrow &\sum_{i,j\in\mathcal{N}} \frac{k_{ij}}{k_i k_j} \Bigg\{ \left(\frac{rc}{k_i+1} - (k_i+1)c\right)\tilde{\tau}_{ij} + rc\sum_{l\in\mathcal{N}_i}\left(\frac{1}{k_i+1}+\frac{1}{k_l+1}\right)(-\tilde{\tau}_{il}+\tilde{\tau}_{jl}) \\
&+ rc\sum_{l\in\mathcal{N}_i}\frac{1}{k_l+1}\sum_{\ell\in\mathcal{N}_l}(-\tilde{\tau}_{i\ell}+\tilde{\tau}_{j\ell}) - \left(\frac{rc}{k_j+1} - (k_j+1)c\right)(-\tilde{\tau}_{ij}) \\
&- rc\sum_{l\in\mathcal{N}_j}\left(\frac{1}{k_j+1}+\frac{1}{k_l+1}\right)(-\tilde{\tau}_{il}+\tilde{\tau}_{jl}) - rc\sum_{l\in\mathcal{N}_j}\frac{1}{k_l+1}\sum_{\ell\in\mathcal{N}_l}(-\tilde{\tau}_{i\ell}+\tilde{\tau}_{j\ell}) \Bigg\} > 0 \\
\Leftrightarrow &\; r > \frac{\sum_{i,j\in\mathcal{N}} \frac{k_{ij}}{k_i k_j} (k_i+k_j+2)\tilde{\tau}_{ij}}{\sum_{i,j\in\mathcal{N}} \frac{k_{ij}}{k_i k_j} [(k_i+1)\tilde{\Upsilon}_{ij} + (k_j+1)\tilde{\Upsilon}_{ji}]}.
\end{aligned} \tag{S77}$$

Further simplifying Eq. (S75) leads to

$$r > \frac{\sum_{i,j\in\mathcal{N}} \frac{k_{ij}}{k_i k_j}(k_i+1)\tilde{\tau}_{ij}}{\sum_{i,j\in\mathcal{N}} \frac{k_{ij}}{k_i k_j}(k_i+1)\tilde{\Upsilon}_{ij}}. \tag{S78}$$

The right-hand side is the $r^\star$ value for the success of cooperation when using accumulated payoffs under the BD rule. The $\tilde{\tau}_{ij}$ values are obtained by solving Eqs. (S67) on the given network, and the $\tilde{\Upsilon}_{ij}$ values are obtained by Eq. (S66).

Supplementary Note 2: Applications to specific networks

With the cooperation conditions obtained in Supplementary Note 1, we can calculate the critical synergy factor for the success of cooperation in PGGs on any given network. Here, we present the calculation process for five examples: regular graphs, star graphs, hub-to-hub star graphs, m-hub star graphs, and fans.

2.1 Regular graphs

The theoretical results of PGG on regular graphs have been previously obtained (*40*), and our framework on any network can reproduce these results when applied to regular networks. On a regular graph, all nodes have the same number of neighbors, $k_i \equiv k$ for $i \in \mathcal{N}$, so $p_{ij} = p_{ji} \equiv 1/k$ for $i, j \in \mathcal{N}$, $i \neq j$.

The calculation for regular graphs is, in fact, the least intuitive compared to other heterogeneous networks when using this framework. We cannot directly solve the system of linear equations for τ_{ij} but instead need to construct intermediate quantities and special recurrence relations for regular graphs (*10*).

We first define

$$\tau_i = 1 + \sum_{j\in\mathcal{N}} p_{ij}\tau_{ij}, \tag{S79}$$

with which we calculate the recurrence relation of $\tau^{(n)}$ defined in the main text:

$$\begin{aligned}
\tau^{(n)} &= \sum_{i,j\in\mathcal{N}} k_i p_{ij}^{(n)} \tau_{ij} \\
&= \sum_{\substack{i,j\in\mathcal{N}\\ i\neq j}} k_i p_{ij}^{(n)} \left(1 + \frac{1}{2}\sum_{l\in\mathcal{N}} p_{il}\tau_{jl} + \frac{1}{2}\sum_{l\in\mathcal{N}} p_{jl}\tau_{il}\right) \\
&= \sum_{i,j\in\mathcal{N}} k_i p_{ij}^{(n)} \left(1 + \frac{1}{2}\sum_{l\in\mathcal{N}} p_{il}\tau_{jl} + \frac{1}{2}\sum_{l\in\mathcal{N}} p_{jl}\tau_{il}\right) - \sum_{i\in\mathcal{N}} k_i p_{ii}^{(n)} \left(1 + \sum_{l\in\mathcal{N}} p_{il}\tau_{il}\right) \\
&= \sum_{i,j\in\mathcal{N}} k_i p_{ij}^{(n)} + \frac{1}{2}\sum_{i,j,l\in\mathcal{N}} k_j p_{ji}^{(n)} p_{il}\tau_{jl} + \frac{1}{2}\sum_{i,j,l\in\mathcal{N}} k_i p_{ij}^{(n)} p_{jl}\tau_{il} - \sum_{i\in\mathcal{N}} k_i p_{ii}^{(n)} \tau_i \\
&= N\langle k\rangle + \frac{1}{2}\sum_{j,l\in\mathcal{N}} k_j p_{jl}^{(n+1)}\tau_{jl} + \frac{1}{2}\sum_{i,l\in\mathcal{N}} k_i p_{il}^{(n+1)}\tau_{il} - \sum_{i\in\mathcal{N}} k_i p_{ii}^{(n)}\tau_i \\
&= N\langle k\rangle + \tau^{(n+1)} - \sum_{i\in\mathcal{N}} k_i p_{ii}^{(n)}\tau_i.
\end{aligned} \tag{S80}$$

Therefore, we have the following recurrence relation:

$$\tau^{(n+1)} = \tau^{(n)} + \sum_{i\in\mathcal{N}} k_i p_{ii}^{(n)}\tau_i - N\langle k\rangle. \tag{S81}$$

The fourth line in Eq. (S80) used the following fact:

$$k_i p_{ij}^{(n)} = \sum_{\ell_1\in\mathcal{N}} k_{i\ell_1} p_{\ell_1 j}^{(n-1)} = \sum_{\ell_1,\ell_2,\ldots,\ell_{n-1}\in\mathcal{N}} k_{i\ell_1}\frac{k_{\ell_1\ell_2}\cdots k_{\ell_{n-1}j}}{k_{\ell_1}\cdots k_{\ell_{n-1}}} = \sum_{\ell_1,\ell_2,\ldots,\ell_{n-1}\in\mathcal{N}} k_{j\ell_{n-1}}\frac{k_{\ell_{n-1}\ell_{n-2}}\cdots k_{\ell_1 i}}{k_{\ell_{n-1}}\cdots k_{\ell_1}} = k_j p_{ji}^{(n)}. \tag{S82}$$

With the help of the recurrence relation Eq. (S81), we can start from $\tau^{(0)}$ and obtain all $\tau^{(n)}$ values step by step. For $i = j$, we have $\tau_{ij} = 0$. Moreover, one stays in the original position if not walking, so $p_{ij}^{(0)} = 1$ if $i = j$ and $p_{ij}^{(0)} = 0$ if $i \neq j$. Therefore, $\tau^{(0)} = \sum_{i,j\in\mathcal{N}} k_i p_{ij}^{(0)}\tau_{ij} = 0$. Substituting these to Eq. (S81), we have the following results:

$$\tau^{(0)} = 0, \tag{S83a}$$

$$\tau^{(1)} = \sum_{i\in\mathcal{N}} k_i\tau_i - N\langle k\rangle, \tag{S83b}$$

$$\tau^{(2)} = \sum_{i\in\mathcal{N}} k_i\tau_i(1 + p_{ii}^{(1)}) - 2N\langle k\rangle, \tag{S83c}$$

$$\tau^{(3)} = \sum_{i\in\mathcal{N}} k_i\tau_i(1 + p_{ii}^{(1)} + p_{ii}^{(2)}) - 3N\langle k\rangle, \tag{S83d}$$

$$\tau^{(4)} = \sum_{i\in\mathcal{N}} k_i\tau_i(1+p_{ii}^{(1)}+p_{ii}^{(2)}+p_{ii}^{(3)}) - 4N\langle k\rangle. \tag{S83e}$$

As declared by Ref. (*10*), there is a relation: $\lim_{n\to\infty} p_{ii}^{(n)} = k_i/(N\langle k\rangle)$. Then, taking $n\to\infty$ in the recurrence relation Eq. (S81), we have

$$\tau^{(\infty)} = \tau^{(\infty)} + \sum_{i\in\mathcal{N}} k_i p_{ii}^{(\infty)}\tau_i - N\langle k\rangle \Leftrightarrow \sum_{i\in\mathcal{N}} k_i^2\tau_i = N^2\langle k\rangle^2. \tag{S84}$$

Supposing all nodes on the regular graph are transitive, we denote $p_{ii}^{(n)} \equiv p^{(n)}$ in shorthand. Obviously, $p^{(1)} = 0$, because we assumed no self-loops on the network, and one cannot leave and return to the same node within a single step; $p^{(2)} = 1/k$, since for each possible first step, the probability of returning to the original node in the second step is $1/k$. The average degree of the network is $\langle k\rangle = k$, so we have $\sum_{i\in\mathcal{N}} k_i\tau_i \equiv N^2k$ according to Eq. (S84). To summarize, Eqs. (S83) can be calculated as

$$\tau^{(0)} = 0, \tag{S85a}$$
$$\tau^{(1)} = \sum_{i\in\mathcal{N}} k_i\tau_i - Nk = (N-1)Nk, \tag{S85b}$$
$$\tau^{(2)} = \sum_{i\in\mathcal{N}} k_i\tau_i - 2Nk = (N-2)Nk, \tag{S85c}$$
$$\tau^{(3)} = \sum_{i\in\mathcal{N}} k_i\tau_i\left(1+\frac{1}{k}\right) - 3Nk = \left[N\left(1+\frac{1}{k}\right)-3\right]Nk, \tag{S85d}$$
$$\tau^{(4)} = \sum_{i\in\mathcal{N}} k_i\tau_i\left(1+\frac{1}{k}+p_{ii}^{(3)}\right) - 4Nk = \left[N\left(1+\frac{1}{k}+p^{(3)}\right)-4\right]Nk. \tag{S85e}$$

Since $k_i \equiv k$ on a regular graph, Υ_{ij} in Eq. (S32) can be simplified as

$$\Upsilon_{ij} = \frac{1}{(k+1)^2}\left(\tau_{ij} + 2k\sum_{l\in\mathcal{N}} p_{il}(\tau_{jl}-\tau_{il}) + k^2\sum_{\ell\in\mathcal{N}} p_{i\ell}^{(2)}(\tau_{j\ell}-\tau_{i\ell})\right). \tag{S86}$$

Now, we can calculate the critical synergy factor for the success of cooperation in PGGs. The general approach is to calculate the numerator $\sum_{i,j\in\mathcal{N}} k_i p_{ij}^{(n)}\tau_{ij}$ and denominator $\sum_{i,j\in\mathcal{N}} k_i p_{ij}^{(n)}\Upsilon_{ij}$ separately, expressing them by $\tau^{(0)}$, $\tau^{(1)}$, $\tau^{(2)}$, etc., and applying the results of Eqs. (S85).

For the PC rule, the numerator of $r^\star$ is

$$\sum_{i,j\in\mathcal{N}} k_i p_{ij}\tau_{ij} = \tau^{(1)}, \tag{S87}$$

and by Eq. (S86), the denominator is

$$\begin{aligned}\sum_{i,j\in\mathcal{N}} k_i p_{ij}\Upsilon_{ij} &= \frac{1}{(k+1)^2}\left(\sum_{i,j\in\mathcal{N}} k_i p_{ij}\tau_{ij} + 2k\sum_{i,j,l\in\mathcal{N}} k_i p_{ij}p_{il}\tau_{jl} - 2k\sum_{i,j,l\in\mathcal{N}} k_i p_{ij}p_{il}\tau_{il}\right.\\ &\quad\left. + k^2\sum_{i,j,\ell\in\mathcal{N}} k_i p_{ij}p_{i\ell}^{(2)}\tau_{j\ell} - k^2\sum_{i,j,\ell\in\mathcal{N}} k_i p_{ij}p_{i\ell}^{(2)}\tau_{i\ell}\right)\\ &= \frac{1}{(k+1)^2}\left(\sum_{i,j\in\mathcal{N}} k_i p_{ij}\tau_{ij} + 2k\sum_{j,l\in\mathcal{N}} k_j p_{jl}^{(2)}\tau_{jl} - 2k\sum_{i,l\in\mathcal{N}} k_i p_{il}\tau_{il}\right.\\ &\quad\left. + k^2\sum_{j,\ell\in\mathcal{N}} k_j p_{j\ell}^{(3)}\tau_{j\ell} - k^2\sum_{i,\ell\in\mathcal{N}} k_i p_{i\ell}^{(2)}\tau_{i\ell}\right)\\ &= \frac{\tau^{(1)} + 2k(\tau^{(2)}-\tau^{(1)}) + k^2(\tau^{(3)}-\tau^{(2)})}{(k+1)^2}.\end{aligned} \tag{S88}$$

Assembling Eq. (S87) and Eq. (S88) and inserting the results of Eqs. (S85), we have

$$r^\star = \frac{\sum_{i,j\in\mathcal{N}} k_i p_{ij}\tau_{ij}}{\sum_{i,j\in\mathcal{N}} k_i p_{ij}\Upsilon_{ij}}$$

$$
\begin{aligned}
&= \frac{(k+1)^2\tau^{(1)}}{\tau^{(1)} + 2k(\tau^{(2)} - \tau^{(1)}) + k^2(\tau^{(3)} - \tau^{(2)})} \\
&= \frac{(N-1)G}{N-G} \xrightarrow{N\to\infty} G,
\end{aligned} \tag{S89}
$$

which is the critical synergy factor for the success of cooperation in PGGs on regular graphs under the PC rule, consistent with the previous research (*31*, *40*). Eq. (S89) has replaced the number of neighbors k by the group size $G = k+1$ for intuitive understanding in PGGs.

For the DB rule, the calculation is similar. The numerator of $r^\star$ is

$$
\sum_{i,j\in\mathcal{N}} k_i p_{ij}^{(2)} \tau_{ij} = \tau^{(2)}, \tag{S90}
$$

and by Eq. (S86), the denominator is

$$
\begin{aligned}
\sum_{i,j\in\mathcal{N}} k_i p_{ij}^{(2)} \Upsilon_{ij} = \frac{1}{(k+1)^2} \Bigg(&\sum_{i,j\in\mathcal{N}} k_i p_{ij}^{(2)} \tau_{ij} + 2k \sum_{i,j,l\in\mathcal{N}} k_i p_{ij}^{(2)} p_{il} \tau_{jl} - 2k \sum_{i,j,l\in\mathcal{N}} k_i p_{ij}^{(2)} p_{il} \tau_{il} \\
&+ k^2 \sum_{i,j,\ell\in\mathcal{N}} k_i p_{ij}^{(2)} p_{i\ell}^{(2)} \tau_{j\ell} - k^2 \sum_{i,j,\ell\in\mathcal{N}} k_i p_{ij}^{(2)} p_{i\ell}^{(2)} \tau_{i\ell} \Bigg) \\
= \frac{1}{(k+1)^2} \Bigg(&\sum_{i,j\in\mathcal{N}} k_i p_{ij}^{(2)} \tau_{ij} + 2k \sum_{j,l\in\mathcal{N}} k_j p_{jl}^{(3)} \tau_{jl} - 2k \sum_{i,l\in\mathcal{N}} k_i p_{il} \tau_{il} \\
&+ k^2 \sum_{j,\ell\in\mathcal{N}} k_j p_{j\ell}^{(4)} \tau_{j\ell} - k^2 \sum_{i,\ell\in\mathcal{N}} k_i p_{i\ell}^{(2)} \tau_{i\ell} \Bigg) \\
= \frac{\tau^{(2)} + 2k(\tau^{(3)} - \tau^{(1)}) + k^2(\tau^{(4)} - \tau^{(2)})}{(k+1)^2}&.
\end{aligned} \tag{S91}
$$

Assembling Eq. (S90) and Eq. (S91) and inserting the results of Eq. (S85), we have

$$
\begin{aligned}
r^\star &= \frac{\sum_{i,j\in\mathcal{N}} k_i p_{ij}^{(2)} \tau_{ij}}{\sum_{i,j\in\mathcal{N}} k_i p_{ij}^{(2)} \Upsilon_{ij}} \\
&= \frac{(k+1)^2\tau^{(2)}}{\tau^{(2)} + 2k(\tau^{(3)} - \tau^{(1)}) + k^2(\tau^{(4)} - \tau^{(2)})} \\
&= \frac{(N-2)G^2}{N(G-1)^2 p^{(3)} + N(G+2) - 2G^2} \\
&= \frac{(N-2)G^2}{N(G-2)\mathscr{C} + N(G+2) - 2G^2} \xrightarrow{N\to\infty} \frac{G^2}{(G-2)\mathscr{C} + G + 2}.
\end{aligned} \tag{S92}
$$

This is the critical synergy factor for the success of cooperation in PGGs on regular graphs under the DB rule, consistent with the previous research (*31*, *33*, *40*). Eq. (S92) has replaced the three-step random walk probability $p^{(3)}$ by clustering coefficient $\mathscr{C} = k^2 p^{(3)}/(k-1)$, which is an intuitive and commonly used concept in network science.

For the BD rule, the recurrence relation Eq. (S68) reduces to the following form on a regular graph:

$$
\begin{cases}
\tilde{\tau}_{ij} = \dfrac{1}{2}\left(1 + \displaystyle\sum_{l\in\mathcal{N}_i} \tilde{\tau}_{jl} + \sum_{l\in\mathcal{N}_j} \tilde{\tau}_{il}\right), & \text{if } j \neq i, \\
\tilde{\tau}_{ij} = 0, & \text{if } j = i.
\end{cases} \tag{S93}
$$

For the critical synergy factor, $\tilde{\tau}_{ij}$ in the numerator and denominator are homogeneous (see Eq. (S69) and Eq. (S66)). Therefore, the critical synergy factor is invariant by replacing $\tilde{\tau}_{ij} \leftarrow \tilde{\tau}^*_{ij}/2$, which makes Eq. (S93) the same recurrence relation for $\tilde{\tau}^*_{ij}$ as the one for τ_{ij} under the PC and DB rules. The solution for $\tilde{\tau}^*_{ij}$ is thus equal to τ_{ij}. On the other hand, we have $k_{ij}/(k_i k_j) = k_i p_{ij}/k^2$ on regular graphs, where k^2 can be canceled simultaneously in the numerator and denominator of the critical synergy factor, making it (Eq. (S69)) the same as the one for the PC rule (Eq. (S34)).

Therefore, on regular graphs, the condition for the success of cooperation in PGGs under the BD rule is equal to the one under the PC rule (i.e., Eq. (S89)).

- Accumulated payoff

On regular graphs, $k_i \equiv k$ for $i \in \mathcal{N}$. Therefore, $k_i + 1$ can be canceled simultaneously in the numerator and denominator in Eq. (S74), Eq. (S76), and Eq. (S78). As a consequence, the critical synergy factors for accumulated payoff are equal to the ones for average payoff under the three update rules (Eq. (S34), Eq. (S46), and Eq. (S69)). For the results under the PC and BD rules, please refer to Eq. (S89), and for the result under the DB rule, please refer to Eq. (S92).

2.2 Star graph

For heterogeneous networks, we can calculate the critical synergy factor by solving the recurrence relation and assembling the resultant τ_{ij} values.

On a star graph, there is one hub (H) and n leaves (L). The hub node has $k_H = n$ neighbors, and each leaf node has $k_L = 1$ neighbor. The values are equal among τ_{ij} of the same type, and for the star graph, there are only two non-zero τ_{ij} types: τ_{HL}, the relation between the hub and a leaf, and $\tau_{LL'}$, the relation between a leaf and another leaf. According to Eq. (4) in the main text, we have the system of linear equations:

$$\begin{cases} \tau_{HL} = 1 + \dfrac{n-1}{2n}\tau_{LL'}, \\ \tau_{LL'} = 1 + \dfrac{1}{2}\tau_{HL} + \dfrac{1}{2}\tau_{HL}. \end{cases} \tag{S94}$$

The solution is

$$\begin{cases} \tau_{HL} = \dfrac{3n-1}{n+1}, \\ \tau_{LL'} = \dfrac{4n}{n+1}. \end{cases} \tag{S95}$$

Unlike τ_{ij}, the values of Υ_{ij} are asymmetric with respect to i and j. Therefore, we need to calculate three Υ_{ij} types: Υ_{HL}, Υ_{LH}, and $\Upsilon_{LL'}$. Inserting the τ_{ij} values of Eq. (S95) into Eq. (5) in the main text, we obtain the required Υ_{ij} values:

$$\begin{aligned} \Upsilon_{HL} &= \frac{1}{k_H+1}\left(\frac{\tau_{HL} + \sum_{l\in\mathcal{N}_H}(\tau_{Ll} - \tau_{Hl})}{k_L+1} + \sum_{l\in\mathcal{N}_H}\frac{(\tau_{Ll} - \tau_{Hl}) + \sum_{\ell\in\mathcal{N}_l}(\tau_{L\ell} - \tau_{H\ell})}{k_l+1}\right) \\ &= \frac{1}{k_H+1}\left\{\frac{\tau_{HL} + [(n-1)\tau_{LL'} - n\tau_{HL}]}{k_H+1}\right. \\ &\quad \left. + \left[\frac{(\tau_{LL} - \tau_{HL}) + (\tau_{HL} - \tau_{HH})}{k_L+1} + (n-1)\frac{(\tau_{LL'} - \tau_{HL}) + (\tau_{HL} - \tau_{HH})}{k_L+1}\right]\right) \\ &= \frac{(n-1)[(n+3)\tau_{LL'} - 2\tau_{HL}]}{2(n+1)^2} \\ &= \frac{2n^2 - n - 1}{(n+1)^2}, \end{aligned} \tag{S96a}$$

$$\begin{aligned} \Upsilon_{LH} &= \frac{1}{k_L+1}\left(\frac{\tau_{HL} + (\tau_{HH} - \tau_{HL})}{k_L+1} + \frac{(\tau_{HH} - \tau_{HL}) + [(\tau_{HL} - \tau_{LL}) + (n-1)(\tau_{HL} - \tau_{LL'})]}{k_H+1}\right) \\ &= \frac{(n-1)(\tau_{HL} - \tau_{LL'})}{2(n+1)} \\ &= -\frac{n-1}{2(n+1)}, \end{aligned} \tag{S96b}$$

$$\begin{aligned} \Upsilon_{LL'} &= \frac{1}{k_L+1}\left(\frac{\tau_{LL'} + \sum_{l\in\mathcal{N}_L}(\tau_{L'l} - \tau_{Ll})}{k_L+1} + \sum_{l\in\mathcal{N}_L}\frac{(\tau_{L'l} - \tau_{Ll}) + \sum_{\ell\in\mathcal{N}_l}(\tau_{L'\ell} - \tau_{L\ell})}{k_l+1}\right) \\ &= \frac{1}{k_L+1}\left(\frac{\tau_{LL'} + (\tau_{L'H} - \tau_{LH})}{k_L+1}\right. \\ &\quad \left. + \frac{(\tau_{L'H} - \tau_{LH}) + [(\tau_{L'L} - \tau_{LL}) + (\tau_{L'L'} - \tau_{LL'}) + (n-2)(\tau_{L'L''} - \tau_{LL'})]}{k_H+1}\right) \end{aligned}$$

$$= \frac{n}{n+1}. \tag{S96c}$$

Some informal variations of the symbols during the calculation are for intuitive understanding. For example, L' refers to "another leaf" and thus $\tau_{L'L'} = \tau_{LL}$, $\tau_{L'L''} = \tau_{LL'}$.

Then, we can apply these τ_{ij} and Υ_{ij} values to calculate the critical synergy factor on the star graph. For the PC rule, the numerator is

$$\begin{aligned}
\tau^{(1)} &= \sum_{i,j\in\mathcal{N}} k_i p_{ij} \tau_{ij} \\
&= k_H(p_{HH}\tau_{HH} + np_{HL}\tau_{HL}) + nk_L(p_{LH}\tau_{LH} + p_{LL}\tau_{LL} + (n-1)p_{LL'}\tau_{LL'}) \\
&= 2n\tau_{HL} \\
&= \frac{2n(3n-1)}{n+1},
\end{aligned} \tag{S97}$$

where the p_{ij} values are obtained by the network directly. For example, $p_{HH} = p_{LL} = 0$ (no self-loops), $p_{LL'} = 0$ (no edges between leaves), $p_{HL} = 1/k_H = 1/n$, $p_{LH} = 1/k_L = 1$. Similarly, the denominator is

$$\begin{aligned}
\Upsilon^{(1)} &= \sum_{i,j\in\mathcal{N}} k_i p_{ij} \Upsilon_{ij} \\
&= k_H(p_{HH}\Upsilon_{HH} + np_{HL}\Upsilon_{HL}) + nk_L(p_{LH}\Upsilon_{LH} + p_{LL}\Upsilon_{LL} + (n-1)p_{LL'}\Upsilon_{LL'}) \\
&= n(\Upsilon_{HL} + \Upsilon_{LH}) \\
&= \frac{n(3n^2-2n-1)}{2(n+1)^2}.
\end{aligned} \tag{S98}$$

Therefore, the critical synergy factor on star graphs under the PC rule is

$$r^\star = \frac{\tau^{(1)}}{\Upsilon^{(1)}} = \frac{4(3n-1)(n+1)}{3n^2-2n-1} \xrightarrow{n\to\infty} 4. \tag{S99}$$

For the DB rule, the numerator is

$$\begin{aligned}
\tau^{(2)} &= \sum_{i,j\in\mathcal{N}} k_i p^{(2)}_{ij} \tau_{ij} \\
&= k_H(p^{(2)}_{HH}\tau_{HH} + np^{(2)}_{HL}\tau_{HL}) + nk_L(p^{(2)}_{LH}\tau_{LH} + p^{(2)}_{LL}\tau_{LL} + (n-1)p^{(2)}_{LL'}\tau_{LL'}) \\
&= (n-1)\tau_{LL'} \\
&= \frac{4n(n-1)}{n+1},
\end{aligned} \tag{S100}$$

where the $p^{(2)}_{ij}$ values are also directly obtained by the network: $p^{(2)}_{HH} = 1$ (the first step must walk to one of the leaves and second step must walk to the hub), $p^{(2)}_{LL} = 1/n$, $p^{(2)}_{LL'} = 1/n$, $p^{(2)}_{HL} = 0$, $p^{(2)}_{LH} = 0$. Similarly, the denominator is

$$\begin{aligned}
\Upsilon^{(2)} &= \sum_{i,j\in\mathcal{N}} k_i p^{(2)}_{ij} \Upsilon_{ij} \\
&= k_H(p^{(2)}_{HH}\Upsilon_{HH} + np^{(2)}_{HL}\Upsilon_{HL}) + nk_L(p^{(2)}_{LH}\Upsilon_{LH} + p^{(2)}_{LL}\Upsilon_{LL} + (n-1)p^{(2)}_{LL'}\Upsilon_{LL'}) \\
&= (n-1)\Upsilon_{LL'} \\
&= \frac{n(n-1)}{n+1}.
\end{aligned} \tag{S101}$$

Therefore, the critical synergy factor on star graphs under the DB rule is

$$r^\star = \frac{\tau^{(2)}}{\Upsilon^{(2)}} \equiv 4. \tag{S102}$$

For the BD rule, we list the system of linear equations according to Eq. (S68):

$$\begin{cases}
\tilde{\tau}_{HL} = \dfrac{n}{n^2+1} + \dfrac{n(n-1)}{n^2+1}\tilde{\tau}_{LL'}, \\
\tilde{\tau}_{LL'} = \dfrac{n}{2} + \tilde{\tau}_{HL}.
\end{cases} \tag{S103}$$

The solution is

$$\begin{cases} \tilde{\tau}_{HL} = \dfrac{n(n^2-n+2)}{2(n+1)}, \\ \tilde{\tau}_{LL'} = \dfrac{n(n^2+3)}{2(n+1)}. \end{cases} \tag{S104}$$

Inserting these $\tilde{\tau}_{ij}$ values into Eq. (S66), we obtain the required $\tilde{\Upsilon}_{ij}$ values:

$$\tilde{\Upsilon}_{HL} = \frac{(n-1)[(n+3)\tilde{\tau}_{LL'} - 2\tilde{\tau}_{HL}]}{2(n+1)^2} = \frac{n(n^3-n^2+5n-5)}{4(n+1)^2}, \tag{S105a}$$

$$\tilde{\Upsilon}_{LH} = \frac{(n-1)(\tilde{\tau}_{HL} - \tilde{\tau}_{LL'})}{2(n+1)} = \frac{n(n-1)}{4(n+1)}. \tag{S105b}$$

Then, we apply these $\tilde{\tau}_{ij}$ and $\tilde{\Upsilon}_{ij}$ values to calculate the critical synergy factor on the star graph under the BD rule. The numerator is

$$\tilde{\tau}^{(1)} = \sum_{i,j\in\mathcal{N}} \frac{k_{ij}}{k_i k_j}\tilde{\tau}_{ij} = 2\tilde{\tau}_{HL} = \frac{n(n^2-n+2)}{n+1}, \tag{S106}$$

and the denominator is

$$\tilde{\Upsilon}^{(1)} = \sum_{i,j\in\mathcal{N}} \frac{k_{ij}}{k_i k_j}\tilde{\Upsilon}_{ij} = \tilde{\Upsilon}_{HL} + \tilde{\Upsilon}_{LH} = \frac{n(n^3-2n^2+5n-4)}{4(n+1)^2}. \tag{S107}$$

Therefore, the critical synergy factor on star graphs under the BD rule is

$$r^\star = \frac{\tilde{\tau}^{(1)}}{\tilde{\Upsilon}^{(1)}} = \frac{4n^3+4n+8}{n^3-2n^2+5n-4} \xrightarrow{n\to\infty} 4. \tag{S108}$$

- Accumulated payoff

When using accumulated payoffs, the values of τ_{ij}, Υ_{ij}, $\tilde{\tau}_{ij}$, and $\tilde{\Upsilon}_{ij}$ keep unchanged, but the formulas of the critical synergy factors are different.

For the PC rule, we follow Eq. (S74). The numerator is

$$\sum_{i,j\in\mathcal{N}} k_i(k_i+1)p_{ij}\tau_{ij} = (n+3)\tau_{HL} = \frac{(3n-1)(n+3)}{n+1}, \tag{S109}$$

and the denominator is

$$\sum_{i,j\in\mathcal{N}} k_i(k_i+1)p_{ij}\Upsilon_{ij} = (n+1)\Upsilon_{HL} + 2\Upsilon_{LH} = \frac{2n(n-1)}{n+1}. \tag{S110}$$

The critical synergy factor on star graphs under the PC rule when using accumulated payoff is

$$r^\star_{\text{accu}} = \frac{(3n-1)(n+3)}{2n(n-1)} \xrightarrow{n\to\infty} \frac{3}{2}. \tag{S111}$$

For the DB rule, we follow Eq. (S76). The numerator is

$$\sum_{i,j\in\mathcal{N}} k_i(k_i+1)p^{(2)}_{ij}\tau_{ij} = 2(n-1)\tau_{LL'} = \frac{8n(n-1)}{n+1}, \tag{S112}$$

and the denominator is

$$\sum_{i,j\in\mathcal{N}} k_i(k_i+1)p^{(2)}_{ij}\Upsilon_{ij} = 2(n-1)\Upsilon_{LL'} = \frac{2n(n-1)}{n+1}. \tag{S113}$$

The critical synergy factor on star graphs under the DB rule when using accumulated payoff is

$$r^{\star}_{\text{accu}} \equiv 4. \tag{S114}$$

For the BD rule, we follow Eq. (S78). The numerator is

$$\sum_{i,j\in\mathscr{N}} \frac{k_{ij}}{k_i k_j}(k_i+1)\tilde{\tau}_{ij} = (n+3)\tilde{\tau}_{HL} = \frac{n(n+3)(n^2-n+2)}{2(n+1)}, \tag{S115}$$

and the denominator is

$$\sum_{i,j\in\mathscr{N}} \frac{k_{ij}}{k_i k_j}(k_i+1)\tilde{\Upsilon}_{ij} = (n+1)\tilde{\Upsilon}_{HL} + 2\tilde{\Upsilon}_{LH} = \frac{n(n^3-n^2+3n-3)}{4(n+1)}. \tag{S116}$$

The critical synergy factor on star graphs under the BD rule when using accumulated payoff is

$$r^{\star}_{\text{accu}} = \frac{2(n+3)(n^2-n+2)}{n^3-n^2+3n-3} \xrightarrow{n\to\infty} 2. \tag{S117}$$

2.3 Hub-to-hub star

On a hub-to-hub star graph, there are two hubs (H), each has n leaves (L). A hub node has $k_H = n+1$ neighbors (n leaves and the other hub), and each leaf node has $k_L = 1$ neighbor. There are five non-zero τ_{ij} types: $\tau_{HH'}$, the relation between the two hubs; $\tau_{LL'}$, the relation between two leaves of the same hub; $\tau_{LL''}$, the relation between a leaf of one hub and another leaf of the other hub; τ_{HL}, the relation between a hub and one of its leaves; $\tau_{HL'}$, the relation between a hub and a leaf of the other hub. According to Eq. (4) in the main text, we have the system of linear equations:

$$\begin{cases} \tau_{HH'} = 1 + \dfrac{n}{n+1}\tau_{HL'}, \\ \tau_{HL} = 1 + \dfrac{1}{2(n+1)}\tau_{HL'} + \dfrac{n-1}{2(n+1)}\tau_{LL'}, \\ \tau_{HL'} = 1 + \dfrac{1}{2(n+1)}\tau_{HL} + \dfrac{n}{2(n+1)}\tau_{LL''} + \dfrac{1}{2}\tau_{HH'}, \\ \tau_{LL'} = 1 + \tau_{HL}, \\ \tau_{LL''} = 1 + \tau_{HL'}. \end{cases} \tag{S118}$$

The solution is

$$\begin{cases} \tau_{HH'} = \dfrac{4n^3+20n^2+17n+5}{(2n+5)(n+1)}, \\ \tau_{HL} = \dfrac{5(2n+1)}{2n+5}, \\ \tau_{HL'} = \dfrac{2(2n^2+9n+5)}{2n+5}, \\ \tau_{LL'} = \dfrac{2(6n+5)}{2n+5}, \\ \tau_{LL''} = \dfrac{4n^2+20n+15}{2n+5}. \end{cases} \tag{S119}$$

Inserting these τ_{ij} values into Eq. (5) in the main text, we obtain the required Υ_{ij} values:

$$\begin{aligned} \Upsilon_{HH'} &= \frac{n}{2(n+2)}(\tau_{HH'} - \tau_{HL} + \tau_{HL'}) \\ &= \frac{n(4n^3+16n^2+15n+5)}{2n^3+11n^2+19n+10}, \end{aligned} \tag{S120a}$$

$$\begin{aligned}\Upsilon_{HL} &= -\frac{2}{(n+2)^2}\tau_{HH'} - \frac{n-2}{(n+2)^2}\tau_{HL} - \frac{n-2}{(n+2)^2}\tau_{HL'} + \frac{n^2+3n-4}{2(n+2)^2}\tau_{LL'} + \frac{2}{(n+2)^2}\tau_{LL''} \\ &= \frac{n(6n^3+21n^2+30n+23)}{(n+2)^2(2n^2+7n+5)}, \end{aligned} \tag{S120b}$$

$$\begin{aligned}\Upsilon_{LH} &= \frac{1}{2(n+2)}\tau_{HH'} + \frac{n-1}{2(n+2)}\tau_{HL} - \frac{1}{2(n+2)}\tau_{HL'} - \frac{n-1}{2(n+2)}\tau_{LL'} \\ &= -\frac{n(2n^2+7n+9)}{4n^3+22n^2+38n+20}, \end{aligned} \tag{S120c}$$

$$\begin{aligned}\Upsilon_{HL'} &= -\frac{2}{(n+2)^2}\tau_{HH'} - \frac{n^2+4n-4}{2(n+2)^2}\tau_{HL} + \frac{n^2+4}{2(n+2)^2}\tau_{HL'} + \frac{n-1}{(n+2)^2}\tau_{LL'} + \frac{n^2+4n}{2(n+2)^2}\tau_{LL''} \\ &= \frac{4n^5+26n^4+64n^3+84n^2+60n+10}{(n+2)^2(2n^2+7n+5)}, \end{aligned} \tag{S120d}$$

$$\begin{aligned}\Upsilon_{LH'} &= \frac{n+4}{4(n+2)}\tau_{HH'} - \frac{n+4}{4(n+2)}\tau_{HL} + \frac{3n}{4(n+2)}\tau_{HL'} - \frac{n-1}{2(n+2)}\tau_{LL'} \\ &= \frac{8n^4+34n^3+53n^2+31n+10}{2(2n^3+11n^2+19n+10)}, \end{aligned} \tag{S120e}$$

$$\begin{aligned}\Upsilon_{LL'} &= \frac{\tau_{LL'}}{4} \\ &= \frac{6n+5}{2(2n+5)}. \end{aligned} \tag{S120f}$$

Then, we apply these τ_{ij} and Υ_{ij} values to calculate the critical synergy factor on the hub-to-hub star graph. For the PC rule, the numerator is

$$\begin{aligned}\tau^{(1)} &= 2\tau_{HH'} + 4n\tau_{HL} \\ &= \frac{2(24n^3+50n^2+27n+5)}{2n^2+7n+5}, \end{aligned} \tag{S121}$$

and the denominator is

$$\begin{aligned}\Upsilon^{(1)} &= 2\Upsilon_{HH'} + 2n\Upsilon_{HL} + 2n\Upsilon_{LH} \\ &= \frac{n(18n^4+79n^3+131n^2+98n+20)}{(n+2)^2(2n^2+7n+5)}. \end{aligned} \tag{S122}$$

Therefore, the critical synergy factor on hub-to-hub star graphs under the PC rule is

$$r^\star = \frac{\tau^{(1)}}{\Upsilon^{(1)}} = \frac{2(n+2)^2(24n^3+50n^2+27n+5)}{n(18n^4+79n^3+131n^2+98n+20)} \xrightarrow{n\to\infty} \frac{8}{3}. \tag{S123}$$

For the DB rule, the numerator is

$$\begin{aligned}\tau^{(2)} &= \sum_{i,j\in\mathcal{N}} k_i p_{ij}^{(2)}\tau_{ij} \\ &= \frac{2n}{n+1}\tau_{HL'} + \frac{2n(n-1)}{n+1}\tau_{LL'} + \frac{2n}{n+1}\tau_{HL'} \\ &= \frac{4n(10n^2+17n+5)}{2n^2+7n+5}, \end{aligned} \tag{S124}$$

and the denominator is

$$\begin{aligned}\Upsilon^{(2)} &= \sum_{i,j\in\mathcal{N}} k_i p_{ij}^{(2)}\Upsilon_{ij} \\ &= \frac{2n}{n+1}\Upsilon_{HL'} + \frac{2n(n-1)}{n+1}\Upsilon_{LL'} + \frac{2n}{n+1}\Upsilon_{L'H} \end{aligned}$$

$$= \frac{n(22n^5+131n^4+287n^3+296n^2+148n+20)}{(2n+5)(n^2+3n+2)^2}. \tag{S125}$$

Therefore, the critical synergy factor on hub-to-hub star graphs under the DB rule is

$$r^\star = \frac{4(n+2)^2(10n^3+27n^2+22n+5)}{22n^5+131n^4+287n^3+296n^2+148n+20} \xrightarrow{n\to\infty} \frac{20}{11}. \tag{S126}$$

For the BD rule, we list the system of linear equations according to Eq. (S68):

$$\begin{cases} \tilde{\tau}_{HH'} = \dfrac{n+1}{2(n^2+n+1)}(1+2n\tilde{\tau}_{HL'}), \\ \tilde{\tau}_{HL} = \dfrac{n+1}{n^2+n+2}\left(1+\dfrac{1}{n+1}\tilde{\tau}_{HL'}+(n-1)\tilde{\tau}_{LL'}\right), \\ \tilde{\tau}_{HL'} = \dfrac{n+1}{n^2+n+2}\left(1+\dfrac{1}{n+1}\tilde{\tau}_{HH'}+\dfrac{1}{n+1}\tilde{\tau}_{HL}+n\tilde{\tau}_{LL''}\right), \\ \tilde{\tau}_{LL'} = \dfrac{n+1}{2}+\tilde{\tau}_{HL}, \\ \tilde{\tau}_{LL''} = \dfrac{n+1}{2}+\tilde{\tau}_{HL'}. \end{cases} \tag{S127}$$

The solution is

$$\begin{cases} \tilde{\tau}_{HH'} = \dfrac{(n+1)(n^5+6n^4+10n^3+12n^2+9n+5)}{2(n^3+3n^2+4n+5)}, \\ \tilde{\tau}_{HL} = \dfrac{2n^5+5n^4+10n^3+11n^2+9n+5}{2(n^3+3n^2+4n+5)}, \\ \tilde{\tau}_{HL'} = \dfrac{n^6+7n^5+17n^4+28n^3+30n^2+23n+10}{2(n^3+3n^2+4n+5)}, \\ \tilde{\tau}_{LL'} = \dfrac{n^5+3n^4+7n^3+9n^2+9n+5}{n^3+3n^2+4n+5}, \\ \tilde{\tau}_{LL''} = \dfrac{n^6+7n^5+18n^4+32n^3+37n^2+32n+15}{2(n^3+3n^2+4n+5)}. \end{cases} \tag{S128}$$

Inserting these $\tilde{\tau}_{ij}$ values into Eq. (S66), we obtain the required $\tilde{\Upsilon}_{ij}$ values:

$$\begin{aligned} \tilde{\Upsilon}_{HH'} &= \frac{n}{2(n+2)}(\tilde{\tau}_{HH'}-\tilde{\tau}_{HL}+\tilde{\tau}_{HL'}) \\ &= \frac{n(n^6+6n^5+14n^4+20n^3+20n^2+14n+5)}{2(n^4+5n^3+10n^2+13n+10)}, \end{aligned} \tag{S129a}$$

$$\begin{aligned} \tilde{\Upsilon}_{HL} &= -\frac{2}{(n+2)^2}\tilde{\tau}_{HH'}-\frac{n-2}{(n+2)^2}\tilde{\tau}_{HL}-\frac{n-2}{(n+2)^2}\tilde{\tau}_{HL'}+\frac{n^2+3n-4}{2(n+2)^2}\tilde{\tau}_{LL'}+\frac{2}{(n+2)^2}\tilde{\tau}_{LL''} \\ &= \frac{n(n^6+4n^5+12n^4+24n^3+36n^2+36n+15)}{2(n+2)^2(n^3+3n^2+4n+5)}, \end{aligned} \tag{S129b}$$

$$\begin{aligned} \tilde{\Upsilon}_{LH} &= \frac{1}{2(n+2)}\tilde{\tau}_{HH'}+\frac{n-1}{2(n+2)}\tilde{\tau}_{HL}-\frac{1}{2(n+2)}\tilde{\tau}_{HL'}-\frac{n-1}{2(n+2)}\tilde{\tau}_{LL'} \\ &= -\frac{n(n^4+4n^3+9n^2+11n+5)}{4(n^4+5n^3+10n^2+13n+10)}. \end{aligned} \tag{S129c}$$

Then, we apply these $\tilde{\tau}_{ij}$ and $\tilde{\Upsilon}_{ij}$ values to calculate the critical synergy factor on the hub-to-hub star graph under the BD rule. The numerator is

$$\tilde{\tau}^{(1)} = \frac{2}{(n+1)^2}\tilde{\tau}_{HH'}+\frac{4n}{n+1}\tilde{\tau}_{HL} = \frac{4n^6+11n^5+26n^4+32n^3+30n^2+19n+5}{n^4+4n^3+7n^2+9n+5}, \tag{S130}$$

and the denominator is

$$\tilde{\Upsilon}^{(1)} = \frac{2}{(n+1)^2}\tilde{\Upsilon}_{HH'} + \frac{2n}{n+1}\tilde{\Upsilon}_{HL} + \frac{2n}{n+1}\tilde{\Upsilon}_{LH} = \frac{n(2n^7+9n^6+32n^5+69n^4+101n^3+107n^2+66n+20)}{2(n+2)^2(n^4+4n^3+7n^2+9n+5)}. \tag{S131}$$

Therefore, the critical synergy factor on hub-to-hub star graphs under the BD rule is

$$r^\star = \frac{\tilde{\tau}^{(1)}}{\tilde{\Upsilon}^{(1)}} = \frac{2(n+2)^2(4n^6+11n^5+26n^4+32n^3+30n^2+19n+5)}{n(2n^7+9n^6+32n^5+69n^4+101n^3+107n^2+66n+20)} \xrightarrow{n\to\infty} 4. \tag{S132}$$

- Accumulated payoff

When using accumulated payoffs, we follow Eq. (S74) for the PC rule. The numerator is

$$\begin{aligned}\sum_{i,j\in\mathscr{N}} k_i(k_i+1)p_{ij}\tau_{ij} &= 2(n+2)\tau_{HH'} + 2n(n+4)\tau_{HL} \\ &= \frac{2(14n^4+83n^3+122n^2+59n+10)}{2n^2+7n+5},\end{aligned} \tag{S133}$$

and the denominator is

$$\begin{aligned}\sum_{i,j\in\mathscr{N}} k_i(k_i+1)p_{ij}\Upsilon_{ij} &= 2(n+2)\Upsilon_{HH'} + 2n(n+2)\Upsilon_{HL} + 4n\Upsilon_{LH} \\ &= \frac{2n(10n^4+43n^3+70n^2+49n+10)}{2n^3+11n^2+19n+10}.\end{aligned} \tag{S134}$$

The critical synergy factor on hub-to-hub star graphs under the PC rule when using accumulated payoff is

$$r^\star_{\text{accu}} = \frac{14n^5+111n^4+288n^3+303n^2+128n+20}{n(10n^4+43n^3+70n^2+49n+10)} \xrightarrow{n\to\infty} \frac{7}{5}. \tag{S135}$$

For the DB rule, we follow Eq. (S76). The numerator is

$$\begin{aligned}\sum_{i,j\in\mathscr{N}} k_i(k_i+1)p^{(2)}_{ij}\tau_{ij} &= \frac{2n(n+4)}{n+1}\tau_{HL'} + \frac{4n(n-1)}{n+1}\tau_{LL'} \\ &= \frac{4n(2n^3+29n^2+39n+10)}{2n^2+7n+5},\end{aligned} \tag{S136}$$

and the denominator is

$$\begin{aligned}\sum_{i,j\in\mathscr{N}} k_i(k_i+1)p^{(2)}_{ij}\Upsilon_{ij} &= \frac{2n(n+2)}{n+1}\Upsilon_{HL'} + \frac{4n}{n+1}\Upsilon_{LH'} + \frac{4n(n-1)}{n+1}\Upsilon_{LL'} \\ &= \frac{2n(4n^5+40n^4+115n^3+141n^2+74n+10)}{(n+1)^2(2n^2+9n+10)}.\end{aligned} \tag{S137}$$

The critical synergy factor on hub-to-hub star graphs under the DB rule when using accumulated payoff is

$$r^\star_{\text{accu}} = \frac{4n^5+70n^4+260n^3+370n^2+216n+40}{4n^5+40n^4+115n^3+141n^2+74n+10} \xrightarrow{n\to\infty} 1. \tag{S138}$$

Eq. (S138) is the result of the super structure for cooperation presented in the main text.

For the BD rule, we follow Eq. (S78). The numerator is

$$\begin{aligned}\sum_{i,j\in\mathscr{N}} \frac{k_{ij}}{k_ik_j}(k_i+1)\tilde{\tau}_{ij} &= \frac{2(n+2)}{(n+1)^2}\tilde{\tau}_{HH'} + \frac{2n(n+4)}{n+1}\tilde{\tau}_{HL} \\ &= \frac{2n^7+14n^6+38n^5+73n^4+85n^3+74n^2+43n+10}{n^4+4n^3+7n^2+9n+5},\end{aligned} \tag{S139}$$

and the denominator is

$$\sum_{i,j\in\mathcal{N}}\frac{k_{ij}}{k_ik_j}(k_i+1)\tilde{\Upsilon}_{ij}=\frac{2(n+2)}{(n+1)^2}\tilde{\Upsilon}_{HH'}+\frac{2n(n+2)}{n+1}\tilde{\Upsilon}_{HL}+\frac{4n}{n+1}\tilde{\Upsilon}_{LH}$$
$$=\frac{n(n^7+5n^6+18n^5+39n^4+56n^3+56n^2+33n+10)}{n^5+6n^4+15n^3+23n^2+23n+10}. \tag{S140}$$

The critical synergy factor on hub-to-hub star graphs under the BD rule when using accumulated payoff is

$$r^\star_{\text{accu}}=\frac{2n^8+18n^7+66n^6+149n^5+231n^4+244n^3+191n^2+96n+20}{n(n^7+5n^6+18n^5+39n^4+56n^3+56n^2+33n+10)}\xrightarrow{n\to\infty}2. \tag{S141}$$

2.4 m-hub star

On an m-hub star graph, there are m hubs (H), each has n leaves (L). A hub node has $k_H=n+m-1$ neighbors (n leaves and the remaining $m-1$ hubs), and each leaf node has $k_L=1$ neighbor. There are five non-zero τ_{ij} types: $\tau_{HH'}$, the relation between one hub and another hub; $\tau_{LL'}$, the relation between two leaves of the same hub; $\tau_{LL''}$, the relation between two leaves of different hubs; τ_{HL}, the relation between a hub and one of its leaves; $\tau_{HL'}$, the relation between a hub and a leaf of another hub. The m-hub star reduces to a hub-to-hub star when $m=2$. According to Eq. (4) in the main text, we have the system of linear equations:

$$\begin{cases}\tau_{HH'}=1+\dfrac{m-2}{n+m-1}\tau_{HH'}+\dfrac{n}{n+m-1}\tau_{HL'},\\ \tau_{HL}=1+\dfrac{m-1}{2(n+m-1)}\tau_{HL'}+\dfrac{n-1}{2(n+m-1)}\tau_{LL'},\\ \tau_{HL'}=1+\dfrac{1}{2(n+m-1)}\tau_{HL}+\dfrac{m-2}{2(n+m-1)}\tau_{HL'}+\dfrac{n}{2(n+m-1)}\tau_{LL''}+\dfrac{1}{2}\tau_{HH'},\\ \tau_{LL'}=1+\tau_{HL},\\ \tau_{LL''}=1+\tau_{HL'}.\end{cases} \tag{S142}$$

The solution is

$$\begin{cases}\tau_{HH'}=\dfrac{2m^3+9m^2n-4m^2+12mn^2-10mn+3m+4n^3-4n^2+n-1}{2m^2+4mn-2m+2n^2-n+1},\\ \tau_{HL}=\dfrac{m^3+4m^2n+m^2+4mn^2+2mn-4m+2n^2-5n+1}{2m^2+4mn-2m+2n^2-n+1},\\ \tau_{HL'}=\dfrac{2m^3+9m^2n-m^2+12mn^2-4mn+4n^3-2n^2-2}{2m^2+4mn-2m+2n^2-n+1},\\ \tau_{LL'}=\dfrac{m^3+4m^2n+3m^2+4mn^2+6mn-6m+4n^2-6n+2}{2m^2+4mn-2m+2n^2-n+1},\\ \tau_{LL''}=\dfrac{2m^3+9m^2n+m^2+12mn^2-2m+4n^3-n-1}{2m^2+4mn-2m+2n^2-n+1}.\end{cases} \tag{S143}$$

Inserting these τ_{ij} values into Eq. (5) in the main text, we obtain the required Υ_{ij} values:

$$\Upsilon_{HH'}=\frac{n}{2(n+m)}(\tau_{HL'}-\tau_{HL}+\tau_{HH'})$$
$$=\frac{n(3m^3+14m^2n-6m^2+20mn^2-16mn+7m+8n^3-8n^2+6n-4}{2(2m^3+6m^2n-2m^2+6mn^2-3mn+m+2n^3-n^2+n)}, \tag{S144a}$$

$$\Upsilon_{HL}=-\frac{m(m-1)}{(m+n)^2}\tau_{HH'}+\frac{m-n}{(m+n)^2}\tau_{HL}+\frac{(m-n)(m-1)}{(m+n)^2}\tau_{HL'}$$
$$+\frac{(n-1)(m+n+2)}{2(m+n)^2}\tau_{LL'}+\frac{n(m-1)}{(m+n)^2}\tau_{LL''}$$

$$= \Big\{ m^4n + 7m^4 + 5m^3n^2 + 22m^3n - 15m^3 + 8m^2n^3 + 21m^2n^2 - 37m^2n - 4m^2 + 4mn^4 + 10mn^3 - 32mn^2 - 4mn + 14m + 4n^4 - 10n^3 + 10n - 4 \Big\} / \Big\{ 2(m+n)^2 \times (2m^2 + 4mn - 2m + 2n^2 - n + 1) \Big\}, \tag{S144b}$$

$$\Upsilon_{LH} = \frac{m-1}{2(m+n)}\tau_{HH'} + \frac{n-1}{2(m+n)}\tau_{HL} - \frac{m-1}{2(m+n)}\tau_{HL'} - \frac{n-1}{2(m+n)}\tau_{LL'} = \frac{-3m^3 - 8m^2n + 8m^2 - 6mn^2 + 13mn - 4m - 2n^3 + 5n^2 - 3n}{2(2m^3 + 6m^2n - 2m^2 + 6mn^2 - 3mn + m + 2n^3 - n^2 + n}, \tag{S144c}$$

$$\Upsilon_{HL'} = -\frac{m(m-1)}{(m+n)^2}\tau_{HH'} + \frac{2m - 2n - mn - n^2}{2(m+n)^2}\tau_{HL} + \frac{2m^2 - mn - 2m + n^2 + 2n}{2(m+n)^2}\tau_{HL'} + \frac{n-1}{(m+n)^2}\tau_{LL'} + \frac{3mn - 2n + n^2}{2(m+n)^2}\tau_{LL''} = \Big\{ 3m^4n + 8m^4 + 17m^3n^2 + 23m^3n - 12m^3 + 34m^2n^3 + 13m^2n^2 - 20m^2n - 10m^2 + 28mn^4 - 6mn^3 - 4mn^2 - 22mn + 16m + 8n^5 - 4n^4 + 4n^3 - 12n^2 + 12n - 4 \Big\} / \Big\{ 2(m+n)^2(2m^2 + 4mn - 2m + 2n^2 - n + 1) \Big\}, \tag{S144d}$$

$$\Upsilon_{LH'} = \frac{3m+n-2}{4(m+n)}\tau_{HH'} - \frac{m+n+2}{4(m+n)}\tau_{HL} + \frac{3n-m+2}{4(m+n)}\tau_{HL'} - \frac{n-1}{2(m+n)}\tau_{LL'} = \Big\{ 3m^4 + 19m^3n - 12m^3 + 44m^2n^2 - 42m^2n + 23m^2 + 44mn^3 - 48mn^2 + 47mn - 12m + 16n^4 - 20n^3 + 26n^2 - 16n \Big\} / \Big\{ 4(2m^3 + 6m^2n - 2m^2 + 6mn^2 - 3mn + m + 2n^3 - n^2 + n) \Big\}, \tag{S144e}$$

$$\Upsilon_{LL'} = \frac{\tau_{LL'}}{4} = \frac{m^3 + 4m^2n + 3m^2 + 4mn^2 + 6mn - 6m + 4n^2 - 6n + 2}{4(2m^2 + 4mn - 2m + 2n^2 - n + 1)}. \tag{S144f}$$

Then, we apply these τ_{ij} and Υ_{ij} values to calculate the critical synergy factor on the m-hub star graph. For the PC rule, the numerator is

$$\tau^{(1)} = m(m-1)\tau_{HH'} + 2mn\tau_{HL} = \Big\{ m(2m^4 + 11m^3n - 6m^3 + 20m^2n^2 - 17m^2n + 7m^2 + 12mn^3 - 12mn^2 + 3mn - 4m - 6n^2 + n + 1) \Big\} / \Big\{ 2m^2 + 4mn - 2m + 2n^2 - n + 1 \Big\}, \tag{S145}$$

and the denominator is

$$\Upsilon^{(1)} = m(m-1)\Upsilon_{HH'} + mn\Upsilon_{HL} + mn\Upsilon_{LH} = \Big\{ mn(3m^5 + 18m^4n - 5m^4 + 39m^3n^2 - 28m^3n + 6m^3 + 36m^2n^3 - 51m^2n^2 + 19m^2n - 19m^2 + 12mn^4 - 34mn^3 + 16mn^2 - 28mn + 18m - 6n^4 + 3n^3 - 9n^2 + 14n - 4) \Big\} / \Big\{ 2(m+n)^2(2m^2 + 4mn - 2m + 2n^2 - n + 1) \Big\}. \tag{S146}$$

Therefore, the critical synergy factor on m-hub star graphs under the PC rule is

$$r^\star = \frac{\text{nume}}{\text{deno}} \xrightarrow{n\to\infty} \frac{4m}{2m-1} \xrightarrow{m=2} \frac{8}{3} \xrightarrow{m\to\infty} 2, \tag{S147}$$

where

$$\text{nume} = 2(m+n)^2(2m^4 + 11m^3n - 6m^3 + 20m^2n^2 - 17m^2n + 7m^2 + 12mn^3$$

$$
\begin{aligned}
&\quad -12mn^2+3mn-4m-6n^2+n+1), \\
\text{deno} &= n(3m^5+18m^4n-5m^4+39m^3n^2-28m^3n+6m^3+36m^2n^3-51m^2n^2+19m^2n \\
&\quad -19m^2+12mn^4-34mn^3+16mn^2-28mn+18m-6n^4+3n^3-9n^2+14n-4).
\end{aligned} \tag{S148}
$$

For the DB rule, the numerator is

$$
\begin{aligned}
\tau^{(2)} &= \frac{m(m-1)(m-2)}{n+m-1}\tau_{HH'} + \frac{2mn(m-1)}{n+m-1}\tau_{HL'} + \frac{mn(n-1)}{n+m-1}\tau_{LL'} \\
&= \Big\{m(2m^4+11m^3n-8m^3+20m^2n^2-25m^2n+11m^2+12mn^3-22mn^2+12mn \\
&\quad -7m-4n^3-2n^2-2n+2)\Big\}/\Big\{2m^2+4mn-2m+2n^2-n+1\Big\},
\end{aligned} \tag{S149}
$$

and the denominator is

$$
\begin{aligned}
\Upsilon^{(2)} &= \frac{m(m-1)(m-2)}{n+m-1}\Upsilon_{HH'} + \frac{mn(m-1)}{n+m-1}\Upsilon_{HL'} + \frac{mn(m-1)}{n+m-1}\Upsilon_{LH'} + \frac{mn(n-1)}{n+m-1}\Upsilon_{LL'} \\
&= \Big\{mn(9m^6+63m^5n-30m^5+171m^4n^2-185m^4n+54m^4+225m^3n^3-414m^3n^2 \\
&\quad +246m^3n-99m^3+144m^2n^4-413m^2n^3+384m^2n^2-256m^2n+114m^2+36mn^5 \\
&\quad -182mn^4+242mn^3-215mn^2+168mn-56m-28n^5+50n^4-58n^3+62n^2 \\
&\quad -40n+8)\Big\}/\Big\{4(m+n)^2(2m^3+6m^2n-4m^2+6mn^2-7mn+3m+2n^3-3n^2 \\
&\quad +2n-1)\Big\}.
\end{aligned} \tag{S150}
$$

Therefore, the critical synergy factor on m-hub star graphs under the DB rule is

$$
r^\star = \frac{\text{nume}}{\text{deno}} \xrightarrow{n\to\infty} \frac{12m-4}{9m-7} \xrightarrow{m=2} \frac{20}{11} \xrightarrow{m\to\infty} \frac{4}{3}, \tag{S151}
$$

where

$$
\begin{aligned}
\text{nume} &= 4(m+n)^2(2m^3+6m^2n-4m^2+6mn^2-7mn+3m+2n^3-3n^2+2n-1) \\
&\quad \times(2m^4+11m^3n-8m^3+20m^2n^2-25m^2n+11m^2+12mn^3-22mn^2+12mn \\
&\quad -7m-4n^3-2n^2-2n+2), \\
\text{deno} &= n(2m^2+4mn-2m+2n^2-n+1)(9m^6+63m^5n-30m^5+171m^4n^2-185m^4n \\
&\quad +54m^4+225m^3n^3-414m^3n^2+246m^3n-99m^3+144m^2n^4-413m^2n^3+384m^2n^2 \\
&\quad -256m^2n+114m^2+36mn^5-182mn^4+242mn^3-215mn^2+168mn-56m-28n^5 \\
&\quad +50n^4-58n^3+62n^2-40n+8).
\end{aligned} \tag{S152}
$$

For the BD rule, we list the system of linear equations according to Eq. (S68):

$$
\begin{cases}
\tilde{\tau}_{HH'} = \dfrac{n+m-1}{2(n^2-n+mn+m-1)}\left(1+\dfrac{2(m-2)}{n+m-1}\tilde{\tau}_{HH'}+2n\tilde{\tau}_{HL'}\right), \\
\tilde{\tau}_{HL} = \dfrac{n+m-1}{n^2-n+mn+m}\left(1+\dfrac{m-1}{n+m-1})\tilde{\tau}_{HL'}+(n-1)\tilde{\tau}_{LL'}\right), \\
\tilde{\tau}_{HL'} = \dfrac{n+m-1}{n^2-n+mn+m}\left(1+\dfrac{1}{n+m-1}\tilde{\tau}_{HL}+\dfrac{m-2}{n+m-1}\tilde{\tau}_{HL'}+n\tilde{\tau}_{LL''}+\dfrac{1}{n+m-1}\tilde{\tau}_{HH'}\right), \\
\tilde{\tau}_{LL'} = \dfrac{n+m-1}{2}+\tilde{\tau}_{HL}, \\
\tilde{\tau}_{LL''} = \dfrac{n+m-1}{2}+\tilde{\tau}_{HL},
\end{cases} \tag{S153}
$$

which can be solved, but the solution for $\tilde{\tau}_{ij}$ is too long and thus not presented here. Inserting these $\tilde{\tau}_{ij}$ values into Eq. (S66), we can obtain the required $\tilde{\Upsilon}_{ij}$ values. Finally, we can apply these $\tilde{\tau}_{ij}$ and $\tilde{\Upsilon}_{ij}$ values to calculate the critical synergy factor on the m-hub star graph under the BD rule. The numerator is

$$\tilde{\tau}^{(1)} = \frac{m(m-1)}{(n+m-1)^2}\tilde{\tau}_{HH'} + \frac{2mn}{n+m-1}\tilde{\tau}_{HL}, \tag{S154}$$

and the denominator is

$$\tilde{\Upsilon}^{(1)} = \frac{m(m-1)}{(n+m-1)^2}\tilde{\Upsilon}_{HH'} + \frac{mn}{n+m-1}\tilde{\Upsilon}_{HL} + \frac{mn}{n+m-1}\tilde{\Upsilon}_{LH}. \tag{S155}$$

Therefore, the critical synergy factor on m-hub star graphs under the BD rule is

$$r^\star = \frac{\tilde{\tau}^{(1)}}{\tilde{\Upsilon}^{(1)}} = \frac{\text{nume}}{\text{deno}} \xrightarrow{n\to\infty} 4, \tag{S156}$$

where

$$\begin{aligned}
\text{nume} = {} & 2(m+n)^2(2m^4n^3 + 2m^4n^2 + 6m^3n^4 - m^3n^3 - 2m^3n^2 + 3m^3n + 6m^2n^5 - 8m^2n^4 + m^2n^3 \\
& + 6m^2n^2 - 3m^2n + 3m^2 + 2mn^6 - 5mn^5 + 10mn^3 - 12mn^2 + 7mn - 4m - 3n^5 + 10n^4 \\
& - 16n^3 + 14n^2 - 7n + 1), \\
\text{deno} = {} & n(m^5n^3 + 4m^5n^2 + 2m^5n + 4m^4n^4 + 10m^4n^3 - 7m^4n^2 + 2m^4n + 2m^4 + 6m^3n^5 + 6m^3n^4 \\
& - 23m^3n^3 + 24m^3n^2 - 7m^3n + 5m^3 + 4m^2n^6 - 2m^2n^5 - 20m^2n^4 + 40m^2n^3 - 35m^2n^2 \\
& + 17m^2n - 17m^2 + mn^7 - 2mn^6 - 9mn^5 + 28mn^4 - 41mn^3 + 23mn^2 - 20mn + 6m \\
& - 3n^6 + 10n^5 - 19n^4 + 15n^3 - 7n^2 - 2n + 4).
\end{aligned} \tag{S157}$$

- Accumulated payoff

When using accumulated payoffs, we follow Eq. (S74) for the PC rule. The numerator is

$$\sum_{i,j\in\mathcal{N}} k_i(k_i+1)p_{ij}\tau_{ij} = m(m+n)(m-1)\tau_{HH'} + mn(m+n+2)\tau_{HL}, \tag{S158}$$

and the denominator is

$$\sum_{i,j\in\mathcal{N}} k_i(k_i+1)p_{ij}\Upsilon_{ij} = m(m+n)(m-1)\Upsilon_{HH'} + mn(m+n)\Upsilon_{HL} + 2mn\Upsilon_{LH}. \tag{S159}$$

The critical synergy factor on m-hub star graphs under the PC rule when using accumulated payoff is

$$r^\star_{\text{accu}} = \frac{\text{nume}}{\text{deno}} \xrightarrow{n\to\infty} \frac{4m-1}{3m-1} \xrightarrow{m\to\infty} \frac{4}{3}, \tag{S160}$$

where

$$\begin{aligned}
\text{nume} = {} & 2(2m^3 + 6m^2n - 2m^2 + 6mn^2 - 3mn + m + 2n^3 - n^2 + n)(2m^5 + 12m^4n - 6m^4 \\
& + 26m^3n^2 - 22m^3n + 7m^3 + 24m^2n^3 - 24m^2n^2 + 16m^2n - 4m^2 + 8mn^4 - 8mn^3 \\
& + 10mn^2 - 12mn + m - 2n^4 + 3n^3 - 10n^2 + 3n), \\
\text{deno} = {} & n(2m^2 + 4mn - 2m + 2n^2 - n + 1)(3m^5 + 18m^4n - 2m^4 + 39m^3n^2 - 17m^3n - 8m^3 \\
& + 36m^2n^3 - 37m^2n^2 - 18m^2n + m^2 + 12mn^4 - 26mn^3 - 14mn^2 + 5mn + 10m \\
& - 4n^4 - 6n^3 + 4n^2 + 8n - 4).
\end{aligned} \tag{S161}$$

For the DB rule, we follow Eq. (S76). The numerator is

$$\sum_{i,j\in\mathcal{N}} k_i(k_i+1)p^{(2)}_{ij}\tau_{ij} = \frac{m(m+n)(m-1)(m-2)}{m+n-1}\tau_{HH'} + \frac{mn(m-1)(m+n+2)}{m+n-1}\tau_{HL'} + \frac{2mn(n-1)}{m+n-1}\tau_{LL'}, \tag{S162}$$

and the denominator is

$$\sum_{i,j\in\mathcal{N}} k_i(k_i+1)p_{ij}^{(2)}\Upsilon_{ij} = \frac{m(m+n)(m-1)(m-2)}{m+n-1}\Upsilon_{HH'} + \frac{mn(m+n)(m-1)}{m+n-1}\Upsilon_{HL'} + \frac{2mn(n-1)}{m+n-1}\Upsilon_{LH'} + \frac{2mn(n-1)}{m+n-1}\Upsilon_{LL'}. \tag{S163}$$

The critical synergy factor on m-hub star graphs under the DB rule when using accumulated payoff is

$$r^\star_{\text{accu}} = \frac{\text{nume}}{\text{deno}} \xrightarrow{n\to\infty} 1, \tag{S164}$$

where

$$\begin{aligned}
\text{nume} = {} & 2(2m^4+8m^3n-4m^3+12m^2n^2-11m^2n+3m^2+8mn^3-10mn^2+5mn-m+2n^4\\
& -3n^3+2n^2-n)(2m^5+11m^4n-8m^4+21m^3n^2-27m^3n+11m^3+16m^2n^3-25m^2n^2\\
& +23m^2n-7m^2+4mn^4-6mn^3+14mn^2-17mn+2m-4n^4+6n^3-18n^2+2n),\\
\text{deno} = {} & n(2m^2+4mn-2m+2n^2-n+1)(3m^6+20m^5n-4m^5+51m^4n^2-33m^4n-5m^4\\
& +62m^3n^3-81m^3n^2+7m^3n-3m^3+36m^2n^4-80m^2n^3+38m^2n^2-15m^2n+23m^2\\
& +8mn^5-36mn^4+32mn^3-22mn^2+23mn-18m-8n^5+8n^4-10n^3+6n^2-6n+4).
\end{aligned} \tag{S165}$$

For the BD rule, we follow Eq. (S78). The numerator is

$$\sum_{i,j\in\mathcal{N}} \frac{k_{ij}}{k_ik_j}(k_i+1)\tilde{\tau}_{ij} = \frac{m(m+n)(m-1)}{(n+m-1)^2}\tilde{\tau}_{HH'} + \frac{mn(m+n+2)}{n+m-1}\tilde{\tau}_{HL}, \tag{S166}$$

and the denominator is

$$\sum_{i,j\in\mathcal{N}} \frac{k_{ij}}{k_ik_j}(k_i+1)\tilde{\Upsilon}_{ij} = \frac{m(m+n)(m-1)}{(n+m-1)^2}\tilde{\Upsilon}_{HH'} + \frac{mn(m+n)}{n+m-1}\tilde{\Upsilon}_{HL} + \frac{2mn}{n+m-1}\tilde{\Upsilon}_{LH}. \tag{S167}$$

The critical synergy factor on m-hub star graphs under the BD rule when using accumulated payoff is

$$r^\star_{\text{accu}} = \frac{\text{nume}}{\text{deno}} \xrightarrow{n\to\infty} 2, \tag{S168}$$

where

$$\begin{aligned}
\text{nume} = {} & 2(m^4n+4m^3n^2-2m^3n+3m^3+6m^2n^3-6m^2n^2+9m^2n-4m^2+4mn^4-6mn^3+9mn^2\\
& -7mn+m+n^5-2n^4+3n^3-3n^2+n)(m^5n^3+2m^5n^2+4m^4n^4+6m^4n^3-4m^4n^2\\
& +3m^4n+6m^3n^5+6m^3n^4-15m^3n^3+15m^3n^2-4m^3n+3m^3+4m^2n^6+2m^2n^5-20m^2n^4\\
& +29m^2n^3-17m^2n^2+10m^2n-4m^2+mn^7-11mn^5+21mn^4-16mn^3+6mn^2-4mn\\
& +m-2n^6+4n^5-n^4-7n^3+10n^2-5n),\\
\text{deno} = {} & n(m^3n+3m^2n^2-2m^2n+3m^2+3mn^3-4mn^2+6mn-4m+n^4-2n^3+3n^2-3n+1)\\
& \times(m^5n^3+4m^5n^2+4m^5n+4m^4n^4+10m^4n^3+m^4n^2-9m^4n+4m^4+6m^3n^5+6m^3n^4\\
& -10m^3n^3-11m^3n^2+22m^3n-5m^3+4m^2n^6-2m^2n^5-9m^2n^4-3m^2n^3+27m^2n^2\\
& -28m^2n-m^2+mn^7-2mn^6-4mn^5+3mn^4+9mn^3-31mn^2+13mn-2m-2n^6\\
& +4n^5-4n^4-6n^3+10n^2-8n+4).
\end{aligned} \tag{S169}$$

2.5 Ceiling fan

On a ceiling fan graph, there is one hub (H) and n leaves (L), each leaf consists of two connected nodes. The hub node has $k_H = 2n$ neighbors, and each leaf node has $k_L = 2$ neighbors (the hub and the other leaf node). There are three non-zero τ_{ij} types: τ_{HL}, the relation between the hub and a leaf node; $\tau_{LL'}$, the relation between the two leaf nodes of the same leaf; $\tau_{LL''}$,

the relation between two leaf nodes of two different leaves. According to Eq. (4) in the main text, we have the system of linear equations:

$$\begin{cases} \tau_{HL} = 1 + \frac{1}{4n}(\tau_{LL'} + (2n-2)\tau_{LL''}) + \frac{1}{4}\tau_{HL}, \\ \tau_{LL'} = 1 + \frac{1}{4}\tau_{HL} + \frac{1}{4}\tau_{HL}, \\ \tau_{LL''} = 1 + \frac{1}{4}(\tau_{HL} + \tau_{LL''}) + \frac{1}{4}(\tau_{HL} + \tau_{LL''}). \end{cases} \tag{S170}$$

The solution is

$$\begin{cases} \tau_{HL} = \frac{2(8n-3)}{2n+3}, \\ \tau_{LL'} = \frac{10n}{2n+3}, \\ \tau_{LL''} = \frac{20n}{2n+3}. \end{cases} \tag{S171}$$

Inserting these τ_{ij} values into Eq. (5) in the main text, we calculate the required Υ_{ij} values:

$$\begin{aligned} \Upsilon_{HL} &= -\frac{4n^2+8n-3}{3(2n+1)^2}\tau_{HL} + \frac{4n+5}{3(2n+1)^2}\tau_{LL'} + \frac{8n^2+2n-10}{3(2n+1)^2}\tau_{LL''} \\ &= \frac{2(16n^3-4n^2-9n-3)}{(2n+1)^2(2n+3)}, \end{aligned} \tag{S172a}$$

$$\begin{aligned} \Upsilon_{LH} &= \frac{10n-1}{9(2n+1)}\tau_{HL} - \frac{4n+5}{9(2n+1)}\tau_{LL'} - \frac{2n-2}{3(2n+1)}\tau_{LL''} \\ &= -\frac{2(n-1)}{3(4n^2+8n+3)}, \end{aligned} \tag{S172b}$$

$$\Upsilon_{LL'} = 0, \tag{S172c}$$

$$\Upsilon_{LL''} = -\frac{2}{9}\tau_{LL'} + \frac{4}{9}\tau_{LL''} = \frac{20n}{6n+9}. \tag{S172d}$$

Then, we apply these τ_{ij} and Υ_{ij} values to calculate the critical synergy factor on the ceiling fan graph. For the PC rule, the numerator is

$$\tau^{(1)} = 4n\tau_{HL} + 2n\tau_{LL'} = \frac{12n(7n-2)}{2n+3}, \tag{S173}$$

and the denominator is

$$\Upsilon^{(1)} = 2n\Upsilon_{HL} + 2n\Upsilon_{LH} + 2n\Upsilon_{LL'} = \frac{8n(24n^3-7n^2-13n-4)}{3(2n+1)^2(2n+3)}. \tag{S174}$$

Therefore, the critical synergy factor on ceiling fan graphs under the PC rule is

$$r^\star = \frac{9(2n+1)^2(7n-2)}{2(24n^3-7n^2-13n-4)} \xrightarrow{n\to\infty} \frac{21}{4}. \tag{S175}$$

For the DB rule, the numerator is

$$\tau^{(2)} = 2n\tau_{HL} + \tau_{LL'} + (2n-2)\tau_{LL''} = \frac{6n(12n-7)}{2n+3}, \tag{S176}$$

and the denominator is

$$\Upsilon^{(2)} = n\Upsilon_{HL} + n\Upsilon_{LH} + \Upsilon_{LL'} + (2n-2)\Upsilon_{LL''} = \frac{4n(64n^3-7n^2-43n-14)}{3(2n+1)^2(2n+3)}. \tag{S177}$$

Therefore, the critical synergy factor on ceiling fan graphs under the DB rule is

$$r^\star = \frac{9(2n+1)^2(12n-7)}{2(64n^3-7n^2-43n-14)} \xrightarrow{n\to\infty} \frac{27}{8}. \tag{S178}$$

For the BD rule, we list the system of linear equations according to Eq. (S68):

$$\begin{cases} \tilde{\tau}_{HL} = \dfrac{2n}{2n^2+n+1}\left(1+\dfrac{1}{2}\tilde{\tau}_{LL'}+(n-1)\tilde{\tau}_{LL''}+\dfrac{1}{2}\tilde{\tau}_{HL}\right), \\ \tilde{\tau}_{LL'} = \dfrac{n}{n+1}\left(1+\dfrac{1}{n}\tilde{\tau}_{HL}\right), \\ \tilde{\tau}_{LL''} = \dfrac{n}{n+1}\left(1+\dfrac{1}{n}\tilde{\tau}_{HL}+\tilde{\tau}_{LL''}\right). \end{cases} \tag{S179}$$

The solution is

$$\begin{cases} \tilde{\tau}_{HL} = \dfrac{2n^4+n^2+2n}{2n^2+2n+1}, \\ \tilde{\tau}_{LL'} = \dfrac{n(2n^2+3)}{2n^2+2n+1}, \\ \tilde{\tau}_{LL''} = \dfrac{n(2n^2+3)(n+1)}{2n^2+2n+1}. \end{cases} \tag{S180}$$

Inserting these $\tilde{\tau}_{ij}$ values into Eq. (S66), we obtain the required $\tilde{\Upsilon}_{ij}$ values:

$$\begin{aligned} \tilde{\Upsilon}_{HL} &= -\frac{4n^2+8n-3}{3(2n+1)^2}\tilde{\tau}_{HL} + \frac{4n+5}{3(2n+1)^2}\tilde{\tau}_{LL'} + \frac{8n^2+2n-10}{3(2n+1)^2}\tilde{\tau}_{LL''} \\ &= \frac{n(8n^5+4n^4+18n^3+4n^2-25n-9)}{3(2n+1)^2(2n^2+2n+1)}, \end{aligned} \tag{S181a}$$

$$\begin{aligned} \tilde{\Upsilon}_{LH} &= \frac{10n-1}{9(2n+1)}\tilde{\tau}_{HL} - \frac{4n+5}{9(2n+1)}\tilde{\tau}_{LL'} - \frac{2n-2}{3(2n+1)}\tilde{\tau}_{LL''} \\ &= \frac{n(8n^4-10n^3-6n^2+7n+1)}{9(4n^3+6n^2+4n+1)}, \end{aligned} \tag{S181b}$$

$$\tilde{\Upsilon}_{LL'} = 0. \tag{S181c}$$

Then, we apply these $\tilde{\tau}_{ij}$ and $\tilde{\Upsilon}_{ij}$ values to calculate the critical synergy factor on the ceiling fan graph under the BD rule. The numerator is

$$\tilde{\tau}^{(1)} = \tilde{\tau}_{HL} + \frac{n}{2}\tilde{\tau}_{LL'} = \frac{n(6n^3+5n+4)}{2(2n^2+2n+1)}, \tag{S182}$$

and the denominator is

$$\tilde{\Upsilon}^{(1)} = \frac{1}{2}\tilde{\Upsilon}_{HL} + \frac{1}{2}\tilde{\Upsilon}_{LH} + \frac{n}{2}\tilde{\Upsilon}_{LL'} = \frac{n(20n^5+16n^3+10n^2-33n-13)}{9(2n+1)^2(2n^2+2n+1)}. \tag{S183}$$

Therefore, the critical synergy factor on ceiling fan graphs under the BD rule is

$$r^\star = \frac{\tilde{\tau}^{(1)}}{\tilde{\Upsilon}^{(1)}} = \frac{9(2n+1)^2(6n^3+5n+4)}{2(20n^5+16n^3+10n^2-33n-13)} \xrightarrow{n\to\infty} \frac{27}{5}. \tag{S184}$$

- Accumulated payoff

When using accumulated payoffs, we follow Eq. (S74) for the PC rule. The numerator is

$$\sum_{i,j\in\mathcal{N}} k_i(k_i+1)p_{ij}\tau_{ij} = 4n(n+2)\tau_{HL} + 6n\tau_{LL'} = \frac{4n(16n^2+41n-12)}{2n+3}, \tag{S185}$$

and the denominator is

$$\sum_{i,j\in\mathcal{N}} k_i(k_i+1)p_{ij}\Upsilon_{ij} = 2n(2n+1)\Upsilon_{HL}+6n\Upsilon_{LH}+6n\Upsilon_{LL'} = \frac{8n(4n^2-3n-1)}{2n+3}. \tag{S186}$$

The critical synergy factor on ceiling fan graphs under the PC rule when using accumulated payoff is

$$r^{\star}_{\text{accu}} = \frac{16n^2+41n-12}{2(4n^2-3n-1)} \xrightarrow{n\to\infty} 2. \tag{S187}$$

For the DB rule, we follow Eq. (S76). The numerator is

$$\begin{aligned}\sum_{i,j\in\mathcal{N}} k_i(k_i+1)p^{(2)}_{ij}\tau_{ij} &= 2n(n+2)\tau_{HL}+3\tau_{LL'}+6(n-1)\tau_{LL''}\\ &= \frac{2n(16n^2+86n-57)}{2n+3},\end{aligned} \tag{S188}$$

and the denominator is

$$\begin{aligned}\sum_{i,j\in\mathcal{N}} k_i(k_i+1)p^{(2)}_{ij}\Upsilon_{ij} &= n(2n+1)\Upsilon_{HL}+3n\Upsilon_{LH}+3\Upsilon_{LL'}+6(n-1)\Upsilon_{LL''}\\ &= \frac{4n(4n^2+7n-11)}{2n+3}.\end{aligned} \tag{S189}$$

The critical synergy factor on ceiling fan graphs under the DB rule when using accumulated payoff is

$$r^{\star}_{\text{accu}} = \frac{16n^2+86n-57}{2(4n^2+7n-11)} \xrightarrow{n\to\infty} 2. \tag{S190}$$

For the BD rule, we follow Eq. (S78). The numerator is

$$\begin{aligned}\sum_{i,j\in\mathcal{N}} \frac{k_{ij}}{k_ik_j}(k_i+1)\tilde{\tau}_{ij} &= (n+2)\tilde{\tau}_{HL}+\frac{3n}{2}\tilde{\tau}_{LL'}\\ &= \frac{n(4n^4+14n^3+2n^2+17n+8)}{2(2n^2+2n+1)},\end{aligned} \tag{S191}$$

and the denominator is

$$\begin{aligned}\sum_{i,j\in\mathcal{N}} \frac{k_{ij}}{k_ik_j}(k_i+1)\tilde{\Upsilon}_{ij} &= \frac{2n+1}{2}\tilde{\Upsilon}_{HL}+\frac{3}{2}\tilde{\Upsilon}_{LH}+\frac{3n}{2}\tilde{\Upsilon}_{LL'}\\ &= \frac{n(2n^4+2n^3+n^2-n-4)}{3(2n^2+2n+1)}.\end{aligned} \tag{S192}$$

The critical synergy factor on ceiling fan graphs under the BD rule when using accumulated payoff is

$$r^{\star}_{\text{accu}} = \frac{3(4n^4+14n^3+2n^2+17n+8)}{2(2n^4+2n^3+n^2-n-4)} \xrightarrow{n\to\infty} 3. \tag{S193}$$

By analogy, the critical synergy factor for PGGs on any other networks can be calculated using the same method in the future.

Supplementary Note 3: Some extensions to the donation game (DG)

Here, we give the details of deducing cooperation conditions in pairwise donation games (DGs). These results can be derived through the techniques in previous literature (*10*). However, only the results under the DB and BD updates using average payoff can be found in previous literature (*10*). To compare our PGGs with pairwise DGs across different model details, we deduce the unpublished results of pairwise DGs and present them in our work.

The actual payoff of agent i is averaged over k_i DGs played with all neighbors $l \in \mathcal{N}_i$. In each DG, a cooperator pays c and the other player receives b ($b > c$), while a defector pays nothing and the other player receives nothing. Namely, the actual payoff $f_i(\mathbf{x})$ of agent i is expressed as follows.

$$f_i(\mathbf{x}) = \frac{1}{k_i}\sum_{l\in\mathcal{N}_i}(-x_i c + x_l b) = -x_i c + \frac{1}{k_i}\sum_{l\in\mathcal{N}_i} x_l b. \tag{S194}$$

The dynamics of strategy evolution under neutral drift remain the same. Only the quantity $\mathbb{E}^{\circ}_{\text{RMC}}[(x_i - x_j)(f_i(\mathbf{x}) - f_j(\mathbf{x}))]$ is influenced by the payoff calculation in pairwise DGs. Applying the payoff in Eq. (S194), we have

$$\begin{aligned}
&\mathbb{E}^{\circ}_{\text{RMC}}[(x_i - x_j)(f_i(\mathbf{x}) - f_j(\mathbf{x}))] \\
&= \mathbb{E}^{\circ}_{\text{RMC}}\left[-(x_i^2 - x_i x_j)c + \frac{1}{k_i}\sum_{l\in\mathcal{N}_i}(x_i x_l - x_j x_l)b + (x_i x_j - x_j^2)c - \frac{1}{k_j}\sum_{l\in\mathcal{N}_j}(x_i x_l - x_j x_l)b\right] \\
&= -c\left(\mathbb{E}^{\circ}_{\text{RMC}}[x_i^2] - 2\mathbb{E}^{\circ}_{\text{RMC}}[x_i x_j] + \mathbb{E}^{\circ}_{\text{RMC}}[x_j^2]\right) + \frac{b}{k_i}\sum_{l\in\mathcal{N}_i}\left(\mathbb{E}^{\circ}_{\text{RMC}}[x_i x_l] - \mathbb{E}^{\circ}_{\text{RMC}}[x_j x_l]\right) \\
&\quad - \frac{b}{k_j}\sum_{l\in\mathcal{N}_j}\left(\mathbb{E}^{\circ}_{\text{RMC}}[x_i x_l] - \mathbb{E}^{\circ}_{\text{RMC}}[x_j x_l]\right).
\end{aligned} \tag{S195}$$

3.1 Pairwise comparison

The cooperation condition under the PC rule is Eq. (S17). Using the result of Eq. (S195) and applying $\tau_{ij} = (1/2 - \mathbb{E}^{\circ}_{\text{RMC}}[x_i x_j])/(K/4)$ defined by Eq. (S26), we calculate the cooperation condition under the PC rule as

$$\begin{aligned}
&\frac{1}{4N^2\langle k\rangle}\sum_{i,j\in\mathcal{N}} k_i p_{ij}\mathbb{E}^{\circ}_{\text{RMC}}[(x_i - x_j)(f_i(\mathbf{x}) - f_j(\mathbf{x}))] > 0 \\
\Leftrightarrow &\sum_{i,j\in\mathcal{N}} k_i p_{ij}\left\{-2c\tau_{ij} + \frac{b}{k_i}\sum_{l\in\mathcal{N}_i}(-\tau_{il} + \tau_{jl}) - \frac{b}{k_j}\sum_{l\in\mathcal{N}_j}(-\tau_{il} + \tau_{jl})\right\} > 0 \\
\Leftrightarrow &\frac{b}{c} > \frac{2\sum_{i,j\in\mathcal{N}} k_i p_{ij}\tau_{ij}}{\sum_{i,j,l\in\mathcal{N}} k_i p_{ij}(p_{il} - p_{jl})(\tau_{jl} - \tau_{il})}.
\end{aligned} \tag{S196}$$

Further simplifying Eq. (S196) (using Eq. (S83)) leads to

$$\frac{b}{c} > \frac{\sum_{i,j\in\mathcal{N}} k_i p_{ij}\tau_{ij}}{\sum_{i,j,l\in\mathcal{N}} k_i p_{ij} p_{il}(\tau_{jl} - \tau_{il})} = \frac{\tau^{(1)}}{\tau^{(2)} - \tau^{(1)}}. \tag{S197}$$

The right-hand side is the $(b/c)^{\star}$ value for the success of cooperation in pairwise DGs under the PC rule. The τ_{ij} values should be obtained by solving Eqs. (S33) on a given network.

3.2 Death-birth

The critical $(b/c)^{\star}$ value under the DB update has been first obtained in Ref. (*10*). Trivially, in our calculation, the cooperation condition under the DB rule is Eq. (S41). Using the result of Eq. (S195) and applying $\tau_{ij} = (1/2 - \mathbb{E}^{\circ}_{\text{RMC}}[x_i x_j])/(K/4)$ defined by Eq. (S44), we calculate Eq. (S41) as

$$\begin{aligned}
&\frac{1}{2N^2\langle k\rangle}\sum_{i,j\in\mathcal{N}} k_i p^{(2)}_{ij}\mathbb{E}^{\circ}_{\text{RMC}}[(x_i - x_j)(f_i(\mathbf{x}) - f_j(\mathbf{x}))] > 0 \\
\Leftrightarrow &\sum_{i,j\in\mathcal{N}} k_i p^{(2)}_{ij}\left\{-2c\tau_{ij} + \frac{b}{k_i}\sum_{l\in\mathcal{N}_i}(-\tau_{il} + \tau_{jl}) - \frac{b}{k_j}\sum_{l\in\mathcal{N}_j}(-\tau_{il} + \tau_{jl})\right\} > 0 \\
\Leftrightarrow &\frac{b}{c} > \frac{2\sum_{i,j\in\mathcal{N}} k_i p^{(2)}_{ij}\tau_{ij}}{\sum_{i,j,l\in\mathcal{N}} k_i p^{(2)}_{ij}(p_{il} - p_{jl})(\tau_{jl} - \tau_{il})}.
\end{aligned} \tag{S198}$$

Further simplifying Eq. (S196) leads to

$$\frac{b}{c} > \frac{\sum_{i,j\in\mathcal{N}} k_i p^{(2)}_{ij}\tau_{ij}}{\sum_{i,j,l\in\mathcal{N}} k_i p^{(2)}_{ij} p_{il}(\tau_{jl} - \tau_{il})} = \frac{\tau^{(2)}}{\tau^{(3)} - \tau^{(1)}}. \tag{S199}$$

The right-hand side is the $(b/c)^\star$ value for the success of cooperation in pairwise DGs under the DB rule, which is consistent with "$t_2/(t_3-t_1)$" in the main text of Ref. (*10*). The τ_{ij} values should be obtained by solving Eqs. (S33) on a given network.

3.3 Birth-death

The critical $(b/c)^\star$ value under the BD update has also been mentioned in Ref. (*10*). We examine their results here. The cooperation condition under the BD rule is Eq. (S53). Using the result of Eq. (S195) and applying $\tau_{ij} = (1/2 - \mathbb{E}^\circ_{\text{RMC}}[x_i x_j])/(K/2)$ as defined by Eq. (S61), we calculate Eq. (S53) follows.

$$
\begin{aligned}
&\frac{1}{2N^2\langle k^{-1}\rangle}\sum_{i,j\in\mathcal{N}}\frac{k_{ij}}{k_i k_j}\mathbb{E}^\circ_{\text{RMC}}[(x_i-x_j)(f_i(\mathbf{x})-f_j(\mathbf{x}))]>0\\
\Leftrightarrow\ &\sum_{i,j\in\mathcal{N}}\frac{k_{ij}}{k_i k_j}\left\{-2c\tilde{\tau}_{ij}+\frac{b}{k_i}\sum_{l\in\mathcal{N}_i}(-\tilde{\tau}_{il}+\tilde{\tau}_{jl})-\frac{b}{k_j}\sum_{l\in\mathcal{N}_j}(-\tilde{\tau}_{il}+\tilde{\tau}_{jl})\right\}>0\\
\Leftrightarrow\ &\frac{b}{c}>\frac{2\sum_{i,j\in\mathcal{N}}\frac{k_{ij}}{k_i k_j}\tilde{\tau}_{ij}}{\sum_{i,j,l\in\mathcal{N}}\frac{k_{ij}}{k_i k_j}(p_{il}-p_{jl})(\tilde{\tau}_{jl}-\tilde{\tau}_{il})}=\frac{\sum_{i,j\in\mathcal{N}}\frac{k_{ij}}{k_i k_j}\tilde{\tau}_{ij}}{\sum_{i,j,l\in\mathcal{N}}\frac{k_{ij}k_{il}}{k_i^2 k_j}(\tilde{\tau}_{jl}-\tilde{\tau}_{il})}.
\end{aligned}
\tag{S200}
$$

The right-hand side is the $(b/c)^\star$ value for the success of cooperation in pairwise DGs under the BD rule. The $\tilde{\tau}_{ij}$ values should be obtained by solving Eqs. (S67) on a given network.

3.4 Variation of the model: accumulated payoff

When using accumulated payoffs, the actual payoff of agent i is accumulated through the k_i DGs played with neighbors. The actual payoff $f_i(\mathbf{x})$ of agent i is

$$
f_i(\mathbf{x})=\sum_{l\in\mathcal{N}_i}(-x_i c+x_l b)=-k_i x_i c+\sum_{l\in\mathcal{N}_i}x_l b. \tag{S201}
$$

The dynamics of strategy evolution under neutral drift remain the same, no matter the payoff calculation is averaged, accumulated or other. Only the quantity $\mathbb{E}^\circ_{\text{RMC}}[(x_i-x_j)(f_i(\mathbf{x})-f_j(\mathbf{x}))]$ is influenced. Applying the accumulated payoff calculation in Eq. (S201), we have

$$
\begin{aligned}
&\mathbb{E}^\circ_{\text{RMC}}[(x_i-x_j)(f_i(\mathbf{x})-f_j(\mathbf{x}))]\\
=\ &\mathbb{E}^\circ_{\text{RMC}}\left[-k_i(x_i^2-x_i x_j)c+\sum_{l\in\mathcal{N}_i}(x_i x_l-x_j x_l)b+k_j(x_i x_j-x_j^2)c-\sum_{l\in\mathcal{N}_j}(x_i x_l-x_j x_l)b\right]\\
=\ &-c\left(k_i\mathbb{E}^\circ_{\text{RMC}}[x_i^2]-(k_i+k_j)\mathbb{E}^\circ_{\text{RMC}}[x_i x_j]+k_j\mathbb{E}^\circ_{\text{RMC}}[x_j^2]\right)+b\sum_{l\in\mathcal{N}_i}\left(\mathbb{E}^\circ_{\text{RMC}}[x_i x_l]-\mathbb{E}^\circ_{\text{RMC}}[x_j x_l]\right)\\
&-b\sum_{l\in\mathcal{N}_j}\left(\mathbb{E}^\circ_{\text{RMC}}[x_i x_l]-\mathbb{E}^\circ_{\text{RMC}}[x_j x_l]\right).
\end{aligned}
\tag{S202}
$$

3.4.1 Pairwise comparison

The cooperation condition under the PC rule is still Eq. (S17). Using the result of Eq. (S195) and Eq. (S26), we calculate

$$
\begin{aligned}
&\frac{1}{4N^2\langle k\rangle}\sum_{i,j\in\mathcal{N}}k_i p_{ij}\mathbb{E}^\circ_{\text{RMC}}[(x_i-x_j)(f_i(\mathbf{x})-f_j(\mathbf{x}))]>0\\
\Leftrightarrow\ &\sum_{i,j\in\mathcal{N}}k_i p_{ij}\left\{-(k_i+k_j)c\tau_{ij}+b\sum_{l\in\mathcal{N}_i}(-\tau_{il}+\tau_{jl})-b\sum_{l\in\mathcal{N}_j}(-\tau_{il}+\tau_{jl})\right\}>0\\
\Leftrightarrow\ &\frac{b}{c}>\frac{\sum_{i,j\in\mathcal{N}}k_i(k_i+k_j)p_{ij}\tau_{ij}}{\sum_{i,j,l\in\mathcal{N}}k_i p_{ij}(k_{il}-k_{jl})(\tau_{jl}-\tau_{il})}=\frac{\sum_{i,j\in\mathcal{N}}k_i^2 p_{ij}\tau_{ij}}{\sum_{i,j,l\in\mathcal{N}}k_i^2 p_{ij}p_{il}(\tau_{jl}-\tau_{il})}.
\end{aligned}
\tag{S203}
$$

The right-hand side is the $(b/c)^\star_{\text{accu}}$ value in pairwise DGs using accumulated payoffs under the PC rule. The τ_{ij} values should be obtained by solving Eqs. (S33) on a given network.

3.4.2 Death-birth

The cooperation condition under the DB rule is still Eq. (S41). Using the result of Eq. (S195) and Eq. (S44), we calculate

$$\begin{aligned}
&\frac{1}{2N^2\langle k\rangle}\sum_{i,j\in\mathcal{N}} k_i p_{ij}^{(2)} \mathbb{E}^{\circ}_{\mathrm{RMC}}[(x_i-x_j)(f_i(\mathbf{x})-f_j(\mathbf{x}))]>0\\
\Leftrightarrow &\sum_{i,j\in\mathcal{N}} k_i p_{ij}^{(2)}\left\{-(k_i+k_j)c\tau_{ij}+b\sum_{l\in\mathcal{N}_i}(-\tau_{il}+\tau_{jl})-b\sum_{l\in\mathcal{N}_j}(-\tau_{il}+\tau_{jl})\right\}>0\\
\Leftrightarrow &\frac{b}{c}>\frac{2\sum_{i,j\in\mathcal{N}} k_i(k_i+k_j)p_{ij}^{(2)}\tau_{ij}}{\sum_{i,j,l\in\mathcal{N}} k_i p_{ij}^{(2)}(k_{il}-k_{jl})(\tau_{jl}-\tau_{il})}=\frac{\sum_{i,j\in\mathcal{N}} k_i^2 p_{ij}^{(2)}\tau_{ij}}{\sum_{i,j,l\in\mathcal{N}} k_i^2 p_{ij}^{(2)} p_{il}(\tau_{jl}-\tau_{il})}.
\end{aligned} \tag{S204}$$

The right-hand side is the $(b/c)^{\star}_{\mathrm{accu}}$ value in pairwise DGs using accumulated payoffs under the DB rule. The τ_{ij} values should be obtained by solving Eqs. (S33) on a given network.

3.4.3 Birth-death

The cooperation condition under the BD rule is still Eq. (S53). Using the result of Eq. (S195) and Eq. (S61), we calculate

$$\begin{aligned}
&\frac{1}{2N^2\langle k^{-1}\rangle}\sum_{i,j\in\mathcal{N}} \frac{k_{ij}}{k_i k_j} \mathbb{E}^{\circ}_{\mathrm{RMC}}[(x_i-x_j)(f_i(\mathbf{x})-f_j(\mathbf{x}))]>0\\
\Leftrightarrow &\sum_{i,j\in\mathcal{N}} \frac{k_{ij}}{k_i k_j}\left\{-(k_i+k_j)c\tilde{\tau}_{ij}+b\sum_{l\in\mathcal{N}_i}(-\tilde{\tau}_{il}+\tilde{\tau}_{jl})-b\sum_{l\in\mathcal{N}_j}(-\tilde{\tau}_{il}+\tilde{\tau}_{jl})\right\}>0\\
\Leftrightarrow &\frac{b}{c}>\frac{\sum_{i,j\in\mathcal{N}} \frac{k_{ij}}{k_i k_j}(k_i+k_j)\tilde{\tau}_{ij}}{\sum_{i,j,l\in\mathcal{N}} \frac{k_{ij}}{k_i k_j}(k_{il}-k_{jl})(\tilde{\tau}_{jl}-\tilde{\tau}_{il})}=\frac{\sum_{i,j\in\mathcal{N}} p_{ij}\tilde{\tau}_{ij}}{\sum_{i,j,l\in\mathcal{N}} p_{ji}p_{il}(\tilde{\tau}_{jl}-\tilde{\tau}_{il})}.
\end{aligned} \tag{S205}$$

The right-hand side is the $(b/c)^{\star}_{\mathrm{accu}}$ value in pairwise DGs using accumulated payoffs under the BD rule. The $\tilde{\tau}_{ij}$ values should be obtained by solving Eqs. (S67) on a given network.

The steps to calculate the cooperation conditions of both PGGs and DGs across all model details (PC, DB, and BD updates & average and accumulated payoffs) are summarized in Fig. S1.

		PC	DB	BD
**STEP 1**		Input edges: $k_{ij} \in \{0,1\}$ ($k_{ji} = k_{ij}$) for all nodes $i, j \in \mathcal{N}$. If $k_{ij} = 1$, then $j \in \mathcal{N}_i$ (j is a neighbor of i). If $k_{ij} = 0$, then $j \notin \mathcal{N}_i$. Calculate necessary quantities: $k_i = \sum_{j\in\mathcal{N}} k_{ij}$, $G_i = k_i + 1$, $p_{ij} = \frac{k_{ij}}{k_i}$, $p_{ij}^{(2)} = \sum_{l\in\mathcal{N}} p_{il}p_{lj}$.		
**STEP 2**		$\begin{cases}\tau_{ij} = 1 + \frac{1}{2k_i}\sum_{l\in\mathcal{N}_i}\tau_{jl} + \frac{1}{2k_j}\sum_{l\in\mathcal{N}_j}\tau_{il}, & j \neq i\\ \tau_{ij} = 0, & j = i\end{cases}$		$\begin{cases}\tilde{\tau}_{ij} = \frac{1}{\sum_{l\in\mathcal{N}_i}k_l^{-1} + \sum_{l\in\mathcal{N}_j}k_l^{-1}}\left(1 + \sum_{l\in\mathcal{N}_i}k_l^{-1}\tilde{\tau}_{jl} + \sum_{l\in\mathcal{N}_j}k_l^{-1}\tilde{\tau}_{il}\right), & j \neq i\\ \tilde{\tau}_{ij} = 0, & j = i\end{cases}$
**STEP 3**	**PGG**	$\Upsilon_{ij} = \frac{1}{G_i}\left(\frac{\tau_{ij} + \sum_{l\in\mathcal{N}_i}(\tau_{jl} - \tau_{il})}{G_i} + \sum_{l\in\mathcal{N}_i}\frac{(\tau_{jl} - \tau_{il}) + \sum_{\ell\in\mathcal{N}_l}(\tau_{j\ell} - \tau_{i\ell})}{G_l}\right)$		$\tilde{\Upsilon}_{ij}$: the same as Υ_{ij}, but just use $\tilde{\tau}_{ij}$ instead of τ_{ij}
		$r^\star = \frac{\sum_{i,j\in\mathcal{N}} k_i p_{ij}\tau_{ij}}{\sum_{i,j\in\mathcal{N}} k_i p_{ij}\Upsilon_{ij}}$	$r^\star = \frac{\sum_{i,j\in\mathcal{N}} k_i p_{ij}^{(2)}\tau_{ij}}{\sum_{i,j\in\mathcal{N}} k_i p_{ij}^{(2)}\Upsilon_{ij}}$	$r^\star = \frac{\sum_{i,j\in\mathcal{N}} \frac{k_{ij}}{k_i k_j}\tilde{\tau}_{ij}}{\sum_{i,j\in\mathcal{N}} \frac{k_{ij}}{k_i k_j}\tilde{\Upsilon}_{ij}}$
		$r^\star_{\text{accu}} = \frac{\sum_{i,j\in\mathcal{N}} k_i(k_i+1)p_{ij}\tau_{ij}}{\sum_{i,j\in\mathcal{N}} k_i(k_i+1)p_{ij}\Upsilon_{ij}}$	$r^\star_{\text{accu}} = \frac{\sum_{i,j\in\mathcal{N}} k_i(k_i+1)p_{ij}^{(2)}\tau_{ij}}{\sum_{i,j\in\mathcal{N}} k_i(k_i+1)p_{ij}^{(2)}\Upsilon_{ij}}$	$r^\star_{\text{accu}} = \frac{\sum_{i,j\in\mathcal{N}} \frac{k_{ij}}{k_i k_j}(k_i+1)\tilde{\tau}_{ij}}{\sum_{i,j\in\mathcal{N}} \frac{k_{ij}}{k_i k_j}(k_i+1)\tilde{\Upsilon}_{ij}}$
	**DG**	$\left(\frac{b}{c}\right)^\star = \frac{\sum_{i,j\in\mathcal{N}} k_i p_{ij}\tau_{ij}}{\sum_{i,j,l\in\mathcal{N}} k_i p_{ij} p_{il}(\tau_{jl} - \tau_{il})}$	$\left(\frac{b}{c}\right)^\star = \frac{\sum_{i,j\in\mathcal{N}} k_i p_{ij}^{(2)}\tau_{ij}}{\sum_{i,j,l\in\mathcal{N}} k_i p_{ij}^{(2)} p_{il}(\tau_{jl} - \tau_{il})}$	$\left(\frac{b}{c}\right)^\star = \frac{\sum_{i,j\in\mathcal{N}} \frac{k_{ij}}{k_i k_j}\tilde{\tau}_{ij}}{\sum_{i,j,l\in\mathcal{N}} \frac{k_{ij}k_{il}}{k_i^2 k_j}(\tilde{\tau}_{jl} - \tilde{\tau}_{il})}$
		$\left(\frac{b}{c}\right)^\star_{\text{accu}} = \frac{\sum_{i,j\in\mathcal{N}} k_i^2 p_{ij}\tau_{ij}}{\sum_{i,j,l\in\mathcal{N}} k_i^2 p_{ij} p_{il}(\tau_{jl} - \tau_{il})}$	$\left(\frac{b}{c}\right)^\star_{\text{accu}} = \frac{\sum_{i,j\in\mathcal{N}} k_i^2 p_{ij}^{(2)}\tau_{ij}}{\sum_{i,j,l\in\mathcal{N}} k_i^2 p_{ij}^{(2)} p_{il}(\tau_{jl} - \tau_{il})}$	$\left(\frac{b}{c}\right)^\star_{\text{accu}} = \frac{\sum_{i,j\in\mathcal{N}} p_{ij}\tilde{\tau}_{ij}}{\sum_{i,j,l\in\mathcal{N}} p_{ji} p_{il}(\tilde{\tau}_{jl} - \tilde{\tau}_{il})}$

Figure S1. Steps to calculate the theoretical conditions for the success of cooperation in both PGGs and DGs, including PC, DB, and BD update rules and average & accumulated payoff calculations. Step 1: Input edges $k_{ij} \in \{0,1\}$ between all nodes and calculate necessary quantities. **Step 2**: Solve for the linear equations to obtain τ_{ij} (for PC and DB updates) or $\tilde{\tau}_{ij}$ (for BD update). Note that $\tau_{ij} = \tau_{ji}$, $\tilde{\tau}_{ij} = \tilde{\tau}_{ji}$. **Step 3**: Insert the obtained values into the formulas for cooperation conditions. Note that usually $\Upsilon_{ij} \neq \Upsilon_{ji}$, $\tilde{\Upsilon}_{ij} \neq \tilde{\Upsilon}_{ji}$. $r^\star$ is the critical synergy factor using average payoff and $r^\star_{\text{accu}}$ is using accumulated payoff, and similar to $(b/c)^\star$ and $(b/c)^\star_{\text{accu}}$.

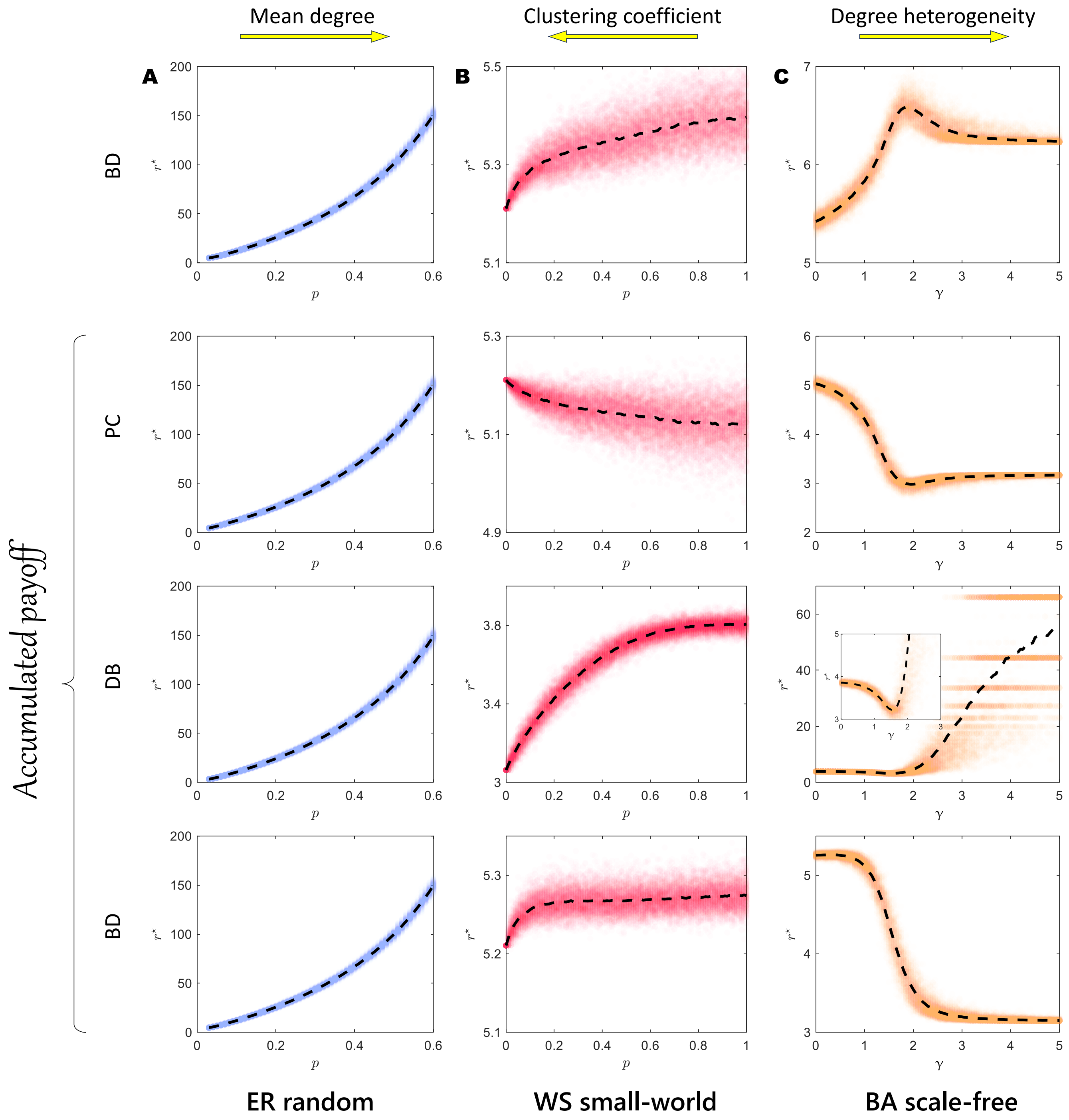

Figure S2. Supplementary results across more model details (BD update and accumulated payoff) for effects of local structures on cooperation in PGGs. (**A**) ER networks. The increasing average degree consistently inhibits cooperation. (**B**) WS networks. The increasing clustering coefficient promotes cooperation, but the PC rule using accumulated payoffs presents the opposite effect. (**C**) BA networks. The increasing degree heterogeneity initially promotes but ultimately inhibits cooperation under the PC and DB rules. However, this does not hold under the BD rule. The parameters are the same as the ones in Fig. 3 in the main text.

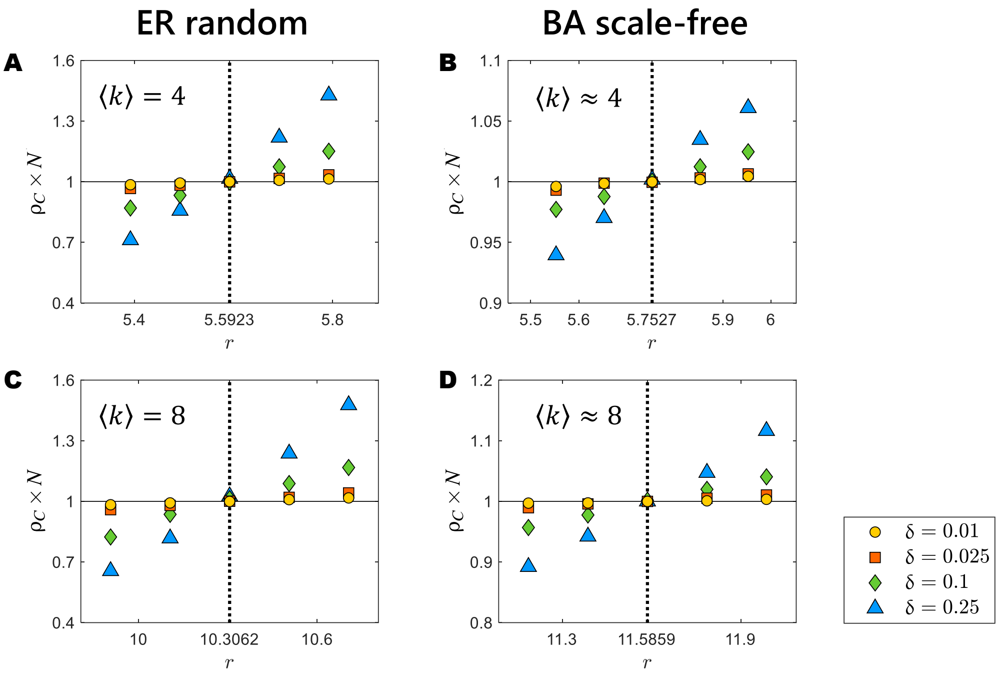

Figure S3. Performance of our theory under non-marginal selection. We examine the results on homogeneous (**A**, **C**) and heterogeneous networks (**B**, **D**) under selection strengths $\delta = 0.01, 0.025, 0.1, 0.25$ (PC rule). The critical threshold derived under weak selection remains accurate for $\delta = 0.1$. Under stronger selection, the theoretical threshold shows a modest deviation from simulation results (e.g., $\delta = 0.25$ in **C**), but the qualitative conclusion remains robust: homogeneous networks support cooperation more effectively than heterogeneous ones ($5.5923 < 5.7527$ and $10.3062 < 11.5859$), consistent with our analytical predictions under weak selection. (**A**) ER network with $\langle k \rangle = 4$ ($p = 4/99$). (**B**) BA network with $\langle k \rangle \approx 4$ ($m = 2$, $\gamma = 2$). (**C**) ER network with $\langle k \rangle = 8$ ($p = 8/99$). (**D**) BA network with $\langle k \rangle \approx 8$ ($m = 4$, $\gamma = 2$). The population size is $N = 100$ for all networks.

$N=3$		$0<r^\star\le 30$	$r^\star>30$	$r^\star<0$ or $r^\star\to\infty$
PGG	PC	1/2	0	*
	DB	1/2	0	*
	BD	1/2	0	*
		$0<(b/c)^\star\le 30$	$(b/c)^\star>30$	$(b/c)^\star<0$ or $(b/c)^\star\to\infty$
DG	PC	0	0	100%
	DB	0	0	100%
	BD	0	0	100%

$N=4$		$0<r^\star\le 30$	$r^\star>30$	$r^\star<0$ or $r^\star\to\infty$
PGG	PC	5/6	0	*
	DB	5/6	0	*
	BD	5/6	0	*
		$0<(b/c)^\star\le 30$	$(b/c)^\star>30$	$(b/c)^\star<0$ or $(b/c)^\star\to\infty$
DG	PC	0	0	100%
	DB	1/6	0	5/6
	BD	0	0	100%

$N=5$		$0<r^\star\le 30$	$r^\star>30$	$r^\star<0$ or $r^\star\to\infty$
PGG	PC	90.48%	4.76%	*
	DB	90.48%	4.76%	*
	BD	90.48%	4.76%	*
		$0<(b/c)^\star\le 30$	$(b/c)^\star>30$	$(b/c)^\star<0$ or $(b/c)^\star\to\infty$
DG	PC	0	0	100%
	DB	23.81%	4.76%	71.43%
	BD	0	0	100%

$N=6$		$0<r^\star\le 30$	$r^\star>30$	$r^\star<0$ or $r^\star\to\infty$
PGG	PC	94.64%	4.46%	*
	DB	95.54%	3.57%	*
	BD	96.43%	2.68%	*
		$0<(b/c)^\star\le 30$	$(b/c)^\star>30$	$(b/c)^\star<0$ or $(b/c)^\star\to\infty$
DG	PC	0	0	100%
	DB	31.25%	6.25%	62.50%
	BD	0	0	100%

$N=7$		$0<r^\star\le 30$	$r^\star>30$	$r^\star<0$ or $r^\star\to\infty$
PGG	PC	97.30%	2.58%	*
	DB	98.12%	1.76%	*
	BD	97.89%	1.99%	*
		$0<(b/c)^\star\le 30$	$(b/c)^\star>30$	$(b/c)^\star<0$ or $(b/c)^\star\to\infty$
DG	PC	0	0	100%
	DB	32.94%	13.13%	53.93%
	BD	0	0	100%

$N=8$		$0<r^\star\le 30$	$r^\star>30$	$r^\star<0$ or $r^\star\to\infty$
PGG	PC	98.81%	1.18%	*
	DB	99.27%	0.72%	*
	BD	99.21%	0.78%	*
		$0<(b/c)^\star\le 30$	$(b/c)^\star>30$	$(b/c)^\star<0$ or $(b/c)^\star\to\infty$
DG	PC	0	0	100%
	DB	31.58%	17.25%	51.17%
	BD	0	0	100%

Figure S4. Supplementary results for PGGs on all networks of different sizes $3 \le N \le 8$. The categories of networks classified by their critical synergy factors are presented. The symbol * means that the only structure that does not support cooperation is the fully connected network. The results are obtained using average payoffs.

Accumulated payoff

$N = 3$		$0 < r^* \leq 30$	$r^* > 30$	$r^* < 0$ or $r^* \to \infty$
PGG	PC	1/2	0	*
	DB	1/2	0	*
	BD	1/2	0	*
		$0 < (b/c)^* \leq 30$	$(b/c)^* > 30$	$(b/c)^* < 0$ or $(b/c)^* \to \infty$
DG	PC	0	0	100%
	DB	0	0	100%
	BD	0	0	100%

$N = 4$		$0 < r^* \leq 30$	$r^* > 30$	$r^* < 0$ or $r^* \to \infty$
PGG	PC	5/6	0	*
	DB	5/6	0	*
	BD	5/6	0	*
		$0 < (b/c)^* \leq 30$	$(b/c)^* > 30$	$(b/c)^* < 0$ or $(b/c)^* \to \infty$
DG	PC	0	0	100%
	DB	1/6	0	5/6
	BD	0	0	100%

$N = 5$		$0 < r^* \leq 30$	$r^* > 30$	$r^* < 0$ or $r^* \to \infty$
PGG	PC	90.48%	4.76%	*
	DB	90.48%	4.76%	*
	BD	90.48%	4.76%	*
		$0 < (b/c)^* \leq 30$	$(b/c)^* > 30$	$(b/c)^* < 0$ or $(b/c)^* \to \infty$
DG	PC	0	0	100%
	DB	23.81%	4.76%	71.43%
	BD	0	0	100%

$N = 6$		$0 < r^* \leq 30$	$r^* > 30$	$r^* < 0$ or $r^* \to \infty$
PGG	PC	96.43%	2.68%	*
	DB	95.54%	3.57%	*
	BD	96.43%	2.68%	*
		$0 < (b/c)^* \leq 30$	$(b/c)^* > 30$	$(b/c)^* < 0$ or $(b/c)^* \to \infty$
DG	PC	0	0	100%
	DB	29.46%	7.14%	63.40%
	BD	0	0	100%

$N = 7$		$0 < r^* \leq 30$	$r^* > 30$	$r^* < 0$ or $r^* \to \infty$
PGG	PC	97.77%	2.11%	*
	DB	98.36%	1.52%	*
	BD	98.94%	0.94%	*
		$0 < (b/c)^* \leq 30$	$(b/c)^* > 30$	$(b/c)^* < 0$ or $(b/c)^* \to \infty$
DG	PC	0	0.12%	99.88%
	DB	29.66%	11.96%	58.38%
	BD	0	0	100%

$N = 8$		$0 < r^* \leq 30$	$r^* > 30$	$r^* < 0$ or $r^* \to \infty$
PGG	PC	99.25%	0.74%	*
	DB	99.40%	0.59%	*
	BD	99.66%	0.33%	*
		$0 < (b/c)^* \leq 30$	$(b/c)^* > 30$	$(b/c)^* < 0$ or $(b/c)^* \to \infty$
DG	PC	0.01%	0.03%	99.96%
	DB	27.57%	17.04%	55.39%
	BD	0	0	100%

Figure S5. Supplementary results to Fig. S4 for PGGs on all networks of different sizes $3 \leq N \leq 8$**.** The results are obtained using accumulated payoffs.

A

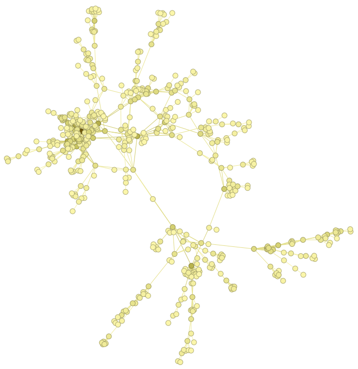

Disease

$\langle k \rangle = 4.60$		$r^\star$	$(b/c)^\star$
ave	PC	5.97	<0
	DB	3.59	4.95
	BD	7.33	<0
accu	PC	5.18	14.60
	DB	3.89	5.88
	BD	6.49	<0

B

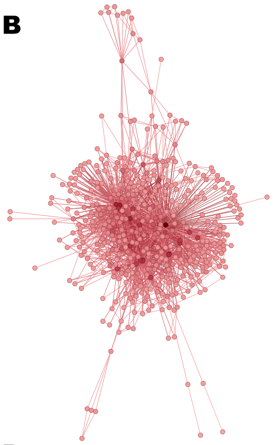

Metabolic

$\langle k \rangle = 8.94$		$r^\star$	$(b/c)^\star$
ave	PC	11.05	<0
	DB	8.02	11.08
	BD	11.86	<0
accu	PC	6.74	15.21
	DB	6.70	11.47
	BD	9.67	<0

C

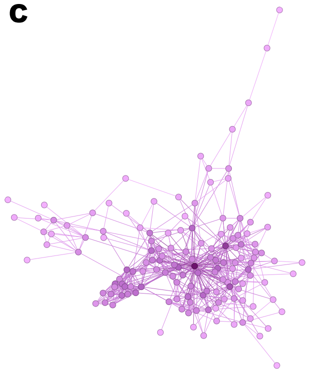

Emails

$\langle k \rangle = 8.71$		$r^\star$	$(b/c)^\star$
ave	PC	11.11	<0
	DB	7.71	10.82
	BD	10.15	<0
accu	PC	10.30	83.59
	DB	8.00	12.57
	BD	9.97	<0

D

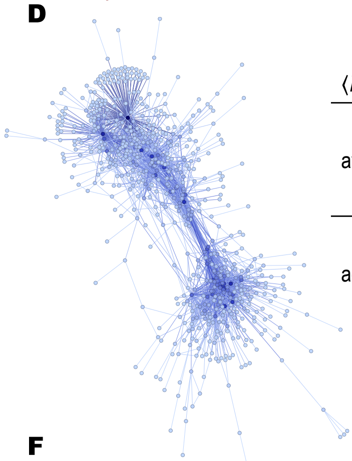

Blogs

$\langle k \rangle = 7.09$		$r^\star$	$(b/c)^\star$
ave	PC	9.20	<0
	DB	6.68	8.16
	BD	10.71	<0
accu	PC	6.04	12.86
	DB	5.76	8.16
	BD	8.33	<0

E

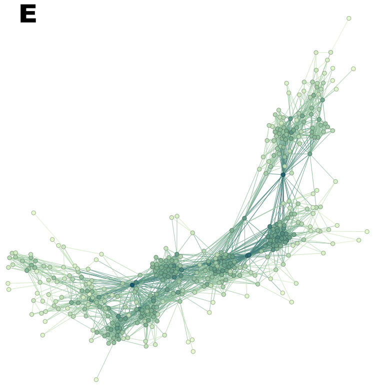

Humans

$\langle k \rangle = 13.49$		$r^\star$	$(b/c)^\star$
ave	PC	15.78	<0
	DB	10.77	15.19
	BD	13.22	<0
accu	PC	16.12	174.01
	DB	11.76	18.13
	BD	14.34	<0

F

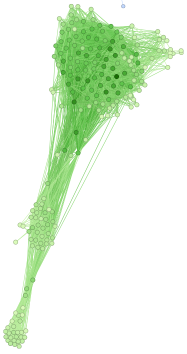

Wild birds

$\langle k \rangle = 45.29$		$r^\star$	$(b/c)^\star$
ave	PC	70.93	<0
	DB	49.54	137.80
	BD	46.97	<0
accu	PC	71.48	<0
	DB	57.00	267.90
	BD	47.00	<0

Figure S6. Supplementary results for more empirical networks. The indicators $(b/c)^\star$ for cooperation in DGs vary depending on model details, whereas $r^\star$ in PGGs remain qualitatively consistent. (**A**) Human disease network of size $N = 516$ (*56*, *87*). (**B**) *C. elegans* metabolic network of size $N = 453$ (*56*, *88*, *89*). (**C**) Email network of size $N = 143$ (*56*). (**D**) Political blog network of size $N = 643$ (*56*, *90*). (**E**) Human contact network of size 410 (*56*, *91*). (**F**) Wild bird network of size $N = 202$ (*56*).

REFERENCES

1. M. A. Nowak, Evolutionary Dynamics: Exploring the Equations of Life (Harvard Univ. Press, 2006).

2. K. Sigmund, *The Calculus of Selfishness* (Princeton Univ. Press, 2010).

3. M. Perc, J. J. Jordan, D. G. Rand, Z. Wang, S. Boccaletti, A. Szolnoki, Statistical physics of human cooperation. *Phys. Rep.* **687**, 1–51 (2017).

4. M. A. Nowak, R. M. May, Evolutionary games and spatial chaos. *Nature* **359**, 826–829 (1992).

5. C. Hauert, M. Doebeli, Spatial structure often inhibits the evolution of cooperation in the snowdrift game. *Nature* **428**, 643–646 (2004).

6. M. Perc, A. Szolnoki, Coevolutionary games—A mini review. *Biosystems* **99**, 109–125 (2010).

7. H. Ohtsuki, C. Hauert, E. Lieberman, M. A. Nowak, A simple rule for the evolution of cooperation on graphs and social networks. *Nature* **441**, 502–505 (2006).

8. P. D. Taylor, T. Day, G. Wild, Evolution of cooperation in a finite homogeneous graph. *Nature* **447**, 469–472 (2007).

9. B. Allen, M. A. Nowak, Games on graphs. *EMS Surv. Math. Sci.* **1**, 113–151 (2014).

10. B. Allen, G. Lippner, Y. T. Chen, B. Fotouhi, N. Momeni, S. T. Yau, M. A. Nowak, Evolutionary dynamics on any population structure. *Nature* **544**, 227–230 (2017).

11. B. Allen, A. McAvoy, A mathematical formalism for natural selection with arbitrary spatial and genetic structure. *J. Math. Biol.* **78**, 1147–1210 (2019).

12. A. McAvoy, B. Allen, Fixation probabilities in evolutionary dynamics under weak selection. *J. Math. Biol.* **82**, 14 (2021).

13. B. Fotouhi, N. Momeni, B. Allen, M. A. Nowak, Conjoining uncooperative societies facilitates evolution of cooperation. *Nat. Hum. Behav.* **2**, 492–499 (2018).

14. A. McAvoy, B. Allen, M. A. Nowak, Social goods dilemmas in heterogeneous societies. *Nat. Hum. Behav.* **4**, 819–831 (2020).

15. C. Hauert, F. Michor, M. A. Nowak, M. Doebeli, Synergy and discounting of cooperation in social dilemmas. *J. Theor. Biol.* **239**, 195–202 (2006).

16. B. Allen, A. R. Khwaja, J. L. Donahue, T. J. Kelly, S. R. Hyacinthe, J. Proulx, C. Lattanzio, Y. A. Dementieva, C. Sample, Nonlinear social evolution and the emergence of collective action. *PNAS Nexus* **3**, pgae131 (2024).

17. J. F. Nash, Equilibrium points in *n*-person games. *Proc. Natl. Acad. Sci. U.S.A.* **36**, 48–49 (1950).

18. M. Perc, J. Gómez-Gardeñes, A. Szolnoki, L. M. Floría, Y. Moreno, Evolutionary dynamics of group interactions on structured populations: A review. *J. R. Soc. Interface* **10**, 20120997 (2013).

19. C. Wang, M. Perc, A. Szolnoki, Evolutionary dynamics of any multiplayer game on regular graphs. *Nat. Commun.* **15**, 5349 (2024).

20. G. Szabó,C. Hauert, Phase transitions and volunteering in spatial public goods games. *Phys. Rev. Lett.* **89**, 118101 (2002).

21. C. Wang, A. Szolnoki, A reversed form of public goods game: Equivalence and difference. *New J. Phys.* **24**, 123030 (2022).

22. C. Wang, C. Huang, Q. Pan, M. He, Modeling the social dilemma of involution on a square lattice. *Chaos Solitons Fractals* **158**, 112092 (2022).

23. G. Hardin, The tragedy of the commons. *Science* **162**, 1243–1248 (1968).

24. D. Semmann, H. J. Krambeck, M. Milinski, Volunteering leads to rock-paper-scissors dynamics in a public goods game. *Nature* **425**, 390–393 (2003).

25. O. P. Hauser, C. Hilbe, K. Chatterjee, M. A. Nowak, Social dilemmas among unequals. *Nature* **572**, 524–527 (2019).

26. L. Shi, I. Romić, Y. Ma, Z. Wang, B. Podobnik, H. E. Stanley, P. Holme, M. Jusup, Freedom of choice adds value to public goods. *Proc. Natl. Acad. Sci. U.S.A.* **117**, 17516–17521 (2020).

27. K. Otten, U. J. Frey, V. Buskens, W. Przepiorka, N. Ellemers, Human cooperation in changing groups in a large-scale public goods game. *Nat. Commun.* **13**, 6399 (2022).

28. F. C. Santos, M. D. Santos, J. M. Pacheco, Social diversity promotes the emergence of cooperation in public goods games. *Nature* **454**, 213–216 (2008).

29. C. Wang, A. Szolnoki, Evolution of cooperation under a generalized death-birth process. *Phys. Rev. E* **107**, 024303 (2023).

30. C. Wang, W. Zhu, A. Szolnoki, The conflict between self-interaction and updating passivity in the evolution of cooperation. *Chaos Solitons Fractals* **173**, 113667 (2023).

31. C. Wang, A. Szolnoki, Inertia in spatial public goods games under weak selection. *Appl. Math Comput.* **449**, 127941 (2023).

32. C. Wang, W. Zhu, A. Szolnoki, When greediness and self-confidence meet in a social dilemma. *Phys. A* **625**, 129033 (2023).

33. X. Wang, L. Zhou, A. M. Avoy, A. Li, Imitation dynamics on networks with incomplete information. *Nat. Commun.* **14**, 7453 (2023).

34. Y. Meng, S. P. Cornelius, Y.-Y. Liu, A. Li, Dynamics of collective cooperation under personalised strategy updates. *Nat. Commun.* **15**, 3125 (2024).

35. R. Ibsen-Jensen, K. Chatterjee, M. A. Nowak, Computational complexity of ecological and evolutionary spatial dynamics. *Proc. Natl. Acad. Sci. U.S.A.* **112**, 15636–15641 (2015).

36. B. Allen, J. Gore, M. A. Nowak, Spatial dilemmas of diffusible public goods. *Elife* **2**, e01169 (2013).

37. A. Li, B. Wu, L. Wang, Cooperation with both synergistic and local interactions can be worse than each alone. *Sci. Rep.* **4**, 5536 (2014).

38. A. Li, M. Broom, J. du, L. Wang, Evolutionary dynamics of general group interactions in structured populations. *Phys. Rev. E* **93**, 022407 (2016).

39. Q. Su, L. Wang, H. E. Stanley, Understanding spatial public goods games on three-layer networks. *New J. Phys.* **20**, 103030 (2018).

40. Q. Su, A. Li, L. Wang, H. E. Stanley, Spatial reciprocity in the evolution of cooperation. *Proc. Biol. Sci.* **286**, 20190041 (2019).

41. G. Szabó, C. Tőke, Evolutionary prisoner's dilemma game on a square lattice. *Phys. Rev. E* **58**, 69 (1998), 73.

42. E. Lieberman, C. Hauert, M. A. Nowak, Evolutionary dynamics on graphs. *Nature* **433**, 312–316 (2005).

43. J. F. C. Kingman, The coalescent. *Stoch. Process Their Appl.* **13**, 235–248 (1982).

44. J. H. Wakeley, *Coalescent Theory: An Introduction* (Roberts and Company Publishers, 2009).

45. J. T. Cox, Coalescing random walks and voter model consensus times on the torus in Z^d. *Ann. Probab.* **17**, 1333–1366 (1989).

46. R. Durrett, S. Levin, The importance of being discrete (and spatial) . *Theor. Popul. Biol.* **46**, 363–394 (1994).

47. P. Erdős, A. Rényi, On random graphs 1. *Publ. Math. (Debrecen)* **6**, 290–297 (1959).

48. D. J. Watts, S. H. Strogatz, Collective dynamics of 'small-world' networks. *Nature* **393**, 440–442 (1998).

49. A. Barrat, M. Weigt, On the properties of small-world network models. *Eur. Phys. J. B.* **13**, 547–560 (2000).

50. A. Sheng, Q. Su, L. Wang, J. B. Plotkin, Strategy evolution on higher-order networks. *Nat. Comput. Sci.* **4**, 274–284 (2024).

51.P. L. Krapivsky, S. Redner, F. Leyvraz, Connectivity of growing random networks. *Phys. Rev. Lett.* **85**, 4629–4632 (2000).

52. A.-L. Barabási, R. Albert, Emergence of scaling in random networks. *Science* **286**, 509–512 (1999).

53. F. L. Pinheiro, D. Hartmann, Intermediate levels of network heterogeneity provide the best evolutionary outcomes. *Sci. Rep.* **7**, 15242 (2017).

54. F. C. Santos, J. M. Pacheco, T. Lenaerts, Evolutionary dynamics of social dilemmas in structured heterogeneous populations. *Proc. Natl. Acad. Sci. U.S.A.* **103**, 3490–3494 (2006).

55. W. Maciejewski, F. Fu, C. Hauert, Evolutionary game dynamics in populations with heterogenous structures. *PLoS Comput. Biol.* **10**, e1003567 (2014).

56. R. A. Rossi, N. K. Ahmed, "The network data repository with interactive graph analytics and visualization," in *Proceedings of the AAAI Conference on Artificial Intelligence* (AAAI, 2015).

57. K. E. Read, Cultures of the central highlands, New Guinea. *Southwest. J. Anthropol.* **10**, 1–43 (1954).

58. M. Vickers, S. Chan, *Representing Classroom Social Structure* (Victoria Institute of Secondary Education, 1981).

59. R. E. Ulanowicz, D. L. DeAngelis, "Network analysis of trophic dynamics in South Florida ecosystems" (FY 97: The Florida Bay Ecosystem, USGS, 1998), pp. 20688–20038.

60. C. J. Melián, J. Bascompte, Food web cohesion. *Ecology* **85**, 352–358 (2004).

61. I. I. Levin, D. M. Zonana, B. K. Fosdick, S. J. Song, R. Knight, R. J. Safran, Stress response, gut microbial diversity and sexual signals correlate with social interactions. *Biol. Lett.* **12**, 20160352 (2016).

62. M. S. Granovetter, The strength of weak ties. *Am. J. Sociol.* **78**, 1360–1380 (1973).

63. M. E. Newman, The structure of scientific collaboration networks. *Proc. Natl. Acad. Sci. U.S.A.* **98**, 404–409 (2001).

64. B. Hao, I. A. Kovács, Proper network randomization is key to assessing social balance *Sci. Adv.* **10**, eadj0104 (2024).

65. C. Hauert, S. de Monte, J. Hofbauer, K. Sigmund, Volunteering as red queen mechanism for cooperation in public goods games. *Science* **296**, 1129–1132 (2002).

66. R. Albert, A.-L. Barabási, Statistical mechanics of complex networks. *Rev. Mod. Phys.* **74**, 47 (2002).

67. F. Battiston, E. Amico, A. Barrat, G. Bianconi, G. Ferraz de Arruda, B. Franceschiello, I. Iacopini, S. Kéfi, V. Latora, Y. Moreno, M. M. Murray, T. P. Peixoto, F. Vaccarino, G. Petri, The physics of higher-order interactions in complex systems. *Nat. Phys.* **17**, 1093–1098 (2021).

68. U. Alvarez-Rodriguez, F. Battiston, G. F. de Arruda, Y. Moreno, M. Perc, V. Latora, Evolutionary dynamics of higher-order interactions in social networks. *Nat. Hum. Behav.* **5**, 586–595 (2021).

69. A. Civilini, O. Sadekar, F. Battiston, J. Gómez-Gardeñes, V. Latora, Explosive cooperation in social dilemmas on higher-order networks. *Phys. Rev. Lett.* **132**, 167401 (2024).

70. M. A. Nowak, Five rules for the evolution of cooperation. *Science* **314**, 1560–1563 (2006).

71. D. G. Rand, M. A. Nowak, J. H. Fowler, N. A. Christakis, Static network structure can stabilize human cooperation. *Proc. Natl. Acad. Sci. U.S.A.* **111**, 17093–17098 (2014).

72. F. C. Santos, J. M. Pacheco, T. Lenaerts, Cooperation prevails when individuals adjust their social ties. *PLoS Comput. Biol.* **2**, e140 (2006).

73. D. G. Rand, S. Arbesman, N. A. Christakis, Dynamic social networks promote cooperation in experiments with humans. *Proc. Natl. Acad. Sci. U.S.A.* **108**, 19193–19198 (2011).

74. Q. Su, A. McAvoy, Y. Mori, J. B. Plotkin, Evolution of prosocial behaviours in multilayer populations. *Nat. Hum. Behav.* **6**, 338–348 (2022).

75. Q. Su, A. McAvoy, J. B. Plotkin, Strategy evolution on dynamic networks. *Nat. Comput. Sci.* **3**, 763–776 (2023).

76. M. Archetti, I. Scheuring, Coexistence of cooperation and defection in public goods games. *Evolution* **65**, 1140–1148 (2011).

77. M. Archetti, I. Scheuring, Game theory of public goods in one-shot social dilemmas without assortment. *J. Theor. Biol.* **299**, 9–20 (2012).

78. J. Pena, Group-size diversity in public goods games. *Evolution* **66**, 623–636 (2012).

79. J. Peña, G. Nöldeke, L. Lehmann, Evolutionary dynamics of collective action in spatially structured populations. *J. Theor. Biol.* **382**, 122–136 (2015).

80. B. Wu, A. Traulsen, C. S. Gokhale, Dynamic properties of evolutionary multi-player games in finite populations. *Games* **4**, 182–199 (2013).

81. A. McAvoy, C. Hauert, Structure coefficients and strategy selection in multiplayer games. *J. Math. Biol.* **72**, 203–238 (2016).

82. P. Clifford, A. Sudbury, A model for spatial conflict. *Biometrika* **60**, 581–588 (1973).

83. J. Gore, H. Youk, A. van Oudenaarden, Snowdrift game dynamics and facultative cheating in yeast. *Nature* **459**, 253–256 (2009).

84. M. A. Nowak, C. E. Tarnita, E. O. Wilson, The evolution of eusociality. *Nature* **466**, 1057–1062 (2010).

85. B. Allen, C. E. Tarnita, Measures of success in a class of evolutionary models with fixed population size and structure. *J. Math. Biol.* **68**, 109–143 (2014).

86. J. Van Cleve, Social evolution and genetic interactions in the short and long term. *Theor. Popul. Biol.* **103**, 2–26 (2015).

87. K.-I. Goh, M. E. Cusick, D. Valle, B. Childs, M. Vidal, A.-L. Barabási, The human disease network. *Proc. Natl. Acad. Sci. U.S.A.* **104**, 8685–8690 (2007).

88. H. Jeong, B. Tombor, R. Albert, Z. N. Oltvai, A. L. Barabási, The large-scale organization of metabolic networks. *Nature* **407**, 651–654 (2000).

89. J. Duch, A. Arenas, Community detection in complex networks using extremal optimization. *Phys. Rev. E* **72**, 027104 (2005).

90. L. A. Adamic, N. Glance, "The political blogosphere and the 2004 U.S. election: Divided they blog," in *Proceedings of the 3rd International Workshop on Link Discovery* (Association for Computing Machinery, 2005), pp. 36–43.

91. SocioPatterns, Infectious contact network, http://sociopatterns.org/datasets.
